# Porphyrins as Promising Photocatalysts for Red-Light-Induced Functionalizations of Biomolecules

**DOI:** 10.1021/acsorginorgau.2c00025

**Published:** 2022-07-15

**Authors:** Katarzyna Rybicka-Jasińska, Tomasz Wdowik, Klaudia Łuczak, Aleksandra J. Wierzba, Olga Drapała, Dorota Gryko

**Affiliations:** Institute of Organic Chemistry, Polish Academy of Sciences, Kasprzaka 44/52, Warsaw 01-224, Poland

**Keywords:** photochemistry, radicals, porphyins, photoredox catalysis, red light, biomolecules

## Abstract

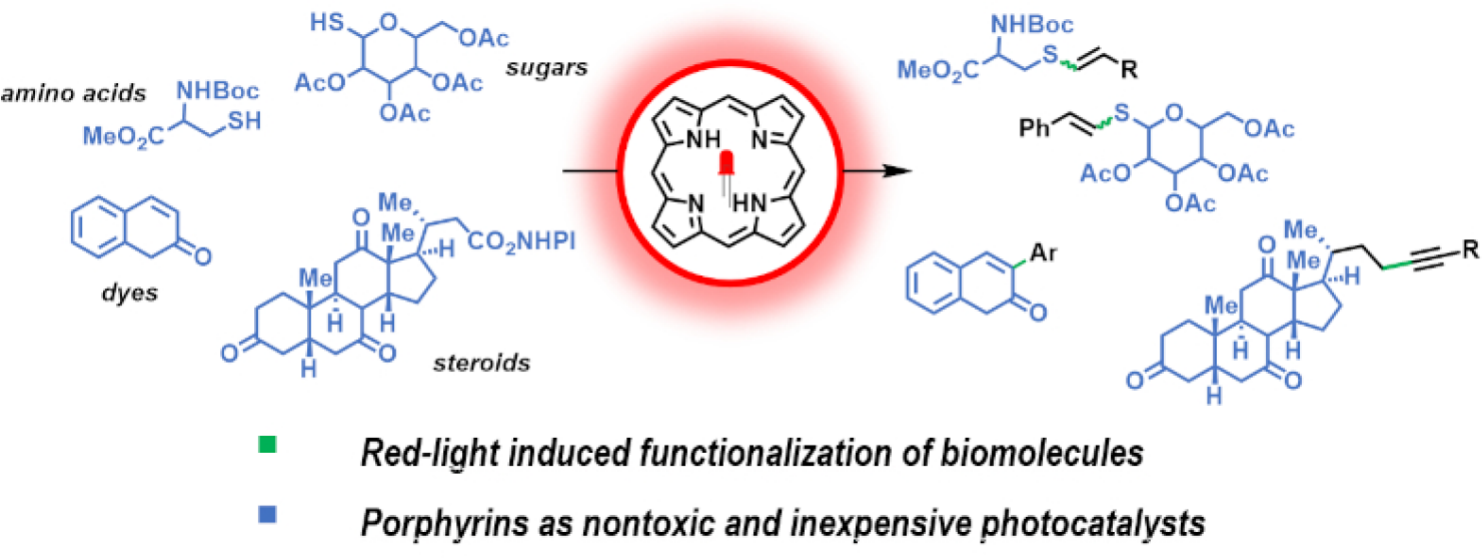

Red-light enables deeper material penetration, which is important for biological applications and has consequences for chemical synthesis. Therefore, the search for new photocatalysts that absorb in this region is crucial. Despite the undeniable utility of porphyrins in blue- and green-light-induced energy- and electron-transfer processes, they are also perfectly suited for red-light applications. Herein, we describe free-base porphyrins as photoredox catalysts for red-light-induced organic transformations. They can act as both photooxidants and photoreductants and can accomplish the synthesis of biaryls once merged with Pd-catalysis. The developed methodology holds promise for broader applications, as the heme-based protoporphyrin is used as a photocatalyst and reactions can be realized in aqueous conditions.

Porphyrinoids are a class of naturally occurring organic dyes that play key roles in the most crucial processes in life (oxygen and electron transport, photosynthesis) due to their versatile photophysical properties.^1^ Given their nontoxicity, solubility in both polar and nonpolar solvents, and either commercial availability or straightforward synthesis,^2,3^ they are perfectly suited for biological applications. In this context, they are used mainly as sensitizers in photodynamic therapy and artificial photosynthesis.^4,5^

Recently, photoredox catalysis has begun to influence molecular biology and medicinal science due to the mild conditions required to generate highly reactive intermediates (e.g., radicals), allowing new and selective functionalizations of biomolecules.^6−8^ Along this line, the use of porphyrins that can transfer either energy (photosensitization) or electrons (photoredox catalysis) under light irradiation seems highly advantageous. Porphyrins already have marked importance in organic synthesis as photosensitizers for singlet oxygen generation and as photoredox catalysts in C–C bond-forming reactions.^9−13^ Because their electronic absorption exhibits the characteristic Soret band at 420 nm with a high molar extinction coefficients (10^5^ M^–1^ cm^–1^, Figure 1),^14^ they have been mainly utilized in blue-light-induced transformations. These molecules do, however, absorb red-light (four Q bands at 518, 553, 592, and 648 nm with molar extinction coefficients of the order of 10^4^ M^–1^ cm^–1^), which has the advantages of low energy, fewer health risks,^15^ and deeper penetration of various media.^16^ Consequently, they have been widely studied as photosensitizers in photodynamic therapy; however, they are only occasionally used as photocatalysts in red-light-induced processes.^13^

**Figure 1:**
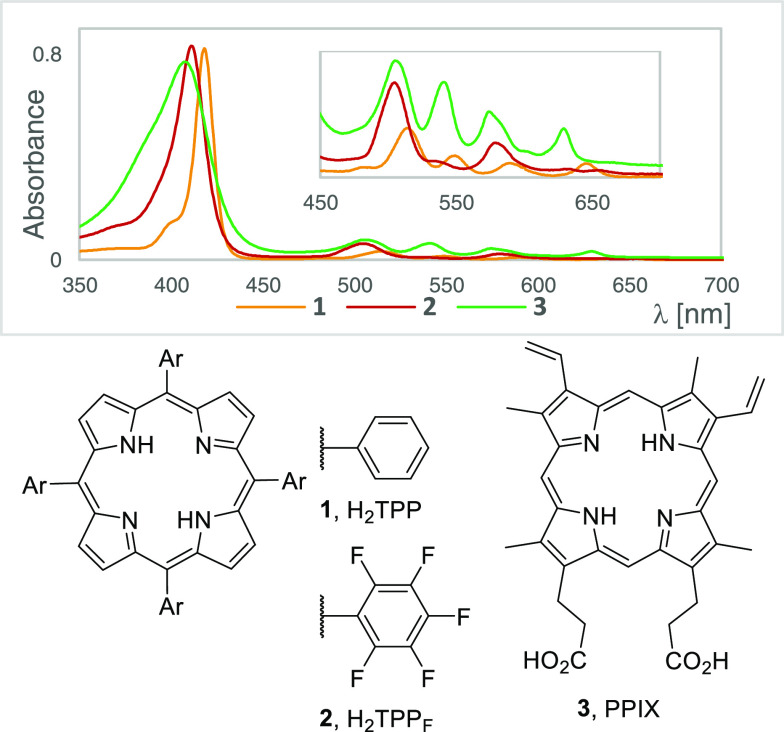
UV–vis spectra and structures of commonly used porphyrins.

Along this line, subphtalocyanines and phtalocyanines proved effective in the perfluoroalkylation of alkenes and alkynes,^17−19^ the cyanation of tertiary amines,^19^ and the photoreductive dehalogenation of α-bromo ketones.^20^ The advantage of using porphyrinoids as photoredox catalysts was recently demonstrated by the MacMillan group, who developed a proximity labeling platform based on a red-light-excited Sn(IV) chlorin e6 that enabled the generation of aminyl radicals both in vitro and in cellulo.^21^ However, to the best of our knowledge, simple free-base *meso*-substituted porphyrins or naturally occurring, nontoxic protoporphyrin IX (PPIX) have remained unexplored in red-light-driven photoredox processes.

In fact, there are only a few photocatalysts that have proved effective in catalyzing the C–C bond-forming reaction under red-light irradiation. Rovis et al. developed Os(II)-based photoredox catalysts that displayed significant S_0_–T_1_ excitation in the deep red (DR) and NIR regions (660–800 nm).^22^ This type of catalyst efficiently catalyzes alkene trifluoromethylation, oxidations, [2 + 2] cycloadditions, and polymerizations. Os complexes are effective, but they have the disadvantages of high toxicity and high cost. Devoid of this problem is the red-light-absorbing helical carbenium ion reported by the Gianetti group, which catalyzes various photochemical reactions that proceed via both oxidative and reductive quenching.^23^ Additionally, cyanine-based NIR organic photoredox catalysts work in both catalytic cycles.^24^ As valuable as these catalysts are, their use in biological systems is rather limited; consequently, photocatalysts that are suitable for biological applications remain to be defined. In this context, porphyrins and especially heme-based PPIX seem highly suitable. As a part of our interest in developing efficient synthetic tools for chemical biology and our ongoing work on porphyrin-type photoredox catalysts, we envisioned that exploring these dyes in red-light transformations might be of importance in biological applications.

## Oxidative Quenching

**Scheme 1:**
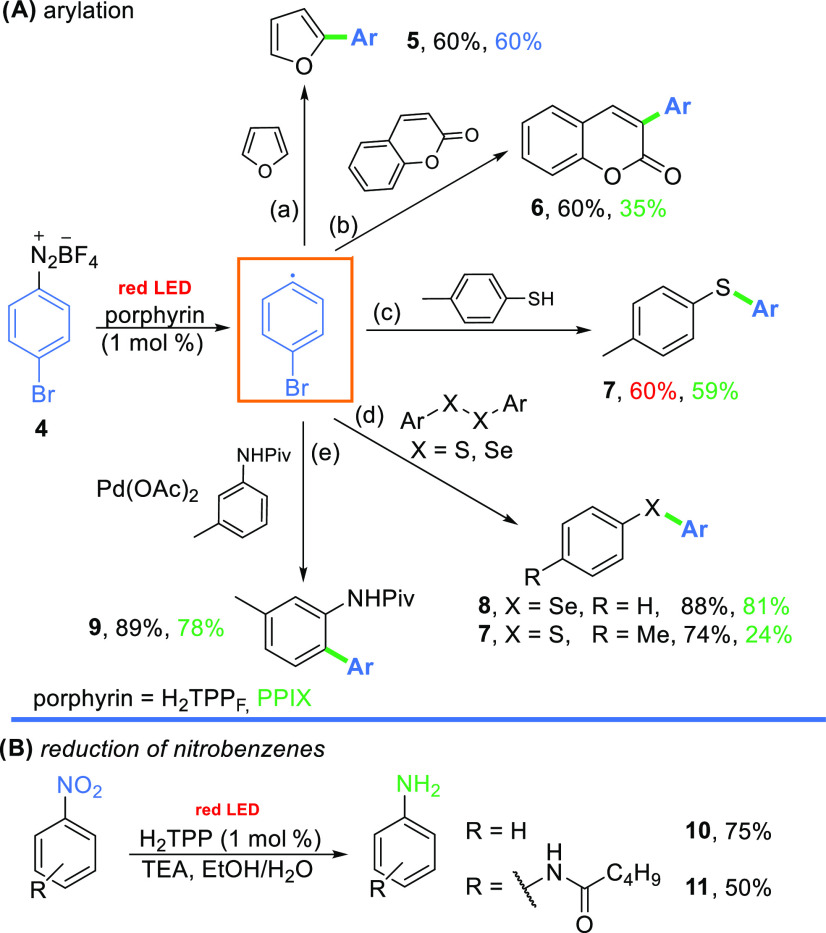
Red-Light-Induced Reactions via Oxidative Quenching (A) Reaction conditions for the red-light-induced (LED, 640 nm) arylation are as follows: (a) diazonium salt (**4**, 0.25 mmol), furan (10 equiv), DMSO (2 mL), and porphyrin (**2**, 1 mol %) for 16 h; (b) diazonium salt (**4**, 0.25 mmol), coumarin (5 equiv), DMSO (2 mL), and porphyrin (1 mol %), for 24 h.; (c) diazonium salt (**4**, 0.25 mmol), thiol (1.1 equiv), DMSO (2 mL), and porphyrin (1 mol %) for 6 h; (d) diazonium salt (**4**, 0.25 mmol), ArXXAr (2 equiv), DMSO (2 mL), and porphyrin (1 mol %) for 6 h; and (e) diazonium salt (**4**, 0.25 mmol), pivalamid (1.1 equiv), Pd(OAc)_2_ (10 mol %), MeOH (1 mL), and porphyrin (1 mol %) for 16 h. (B) Reaction conditions for the red-light-induced (LED, 640 nm) reduction of nitrobenzenes are as follows: nitrobenzene (0.2 mmol), TEA (6 equiv), EtOH (3 mL), H_2_O (2 mL), and porphyrin (1 mol %) for 24 h.

It is well-documented that porphyrins can act as photoreductants in blue-light-induced transformations, for example, in the C–H arylation of heteroarenes with aryldiazonium salts.^25^ This process involves the generation of aryl radicals through single-electron transfer (SET) from the excited porphyrin to diazonium salts and the subsequent addition of the radical to the heteroarene.^25^ To assess the effectiveness of porphyrins in catalyzing processes under red-light irradiation, the model reaction of furan with diazonium salt **4** in the presence of H_2_TPP_F_ (**2**), which effectively catalyzed this reaction under blue-light irradiation, was performed (Scheme 1).^25^ The reaction furnished the desired product **5** in a 60% yield (Scheme 1A). This result confirms that less-energetic red light is indeed sufficient for the generation of radicals from diazonium salts. Of importance is the fact that synthetic porphyrin **2** can be replaced with less expensive and heme-based PPIX (**3**, 60%). To fully access the photoreductive power of free-base porphyrins (H_2_TPP_F_**2** and PPIX **3**), we tested them in other transformations, namely, the arylation of coumarins,^25^ thiols,^26^ diselenides,^27^ and disulfides^27^ with diazonium salts. Red-light-induced arylations proceed with decent yields even without the fine-tuning of the reaction conditions.

When merged within palladium catalysis, porphyrins efficiently catalyzed the synthesis of biaryls. The reactions gave the desired compounds in high yields regardless of the porphyrin used (89 and 78%).

This study represents the first application of free-base porphyrins in a dual photoredox–metal catalytic system. Importantly, the metalation of the catalysts during the process, which could alter the catalytic properties of the porphyrins, was not observed.

The red-light-induced oxidative quenching pathway is not limited to reactions with aryldiazonium salts, as it also enables the formation of anilines from nitrobenzenes.^28^ The reaction proved to be efficient without any further optimization, producing aniline **10** and *N*-(4-nitrophenyl)-2-propylpentanamide **11** (Scheme 1B).

## Reductive Quenching

**Scheme 2:**
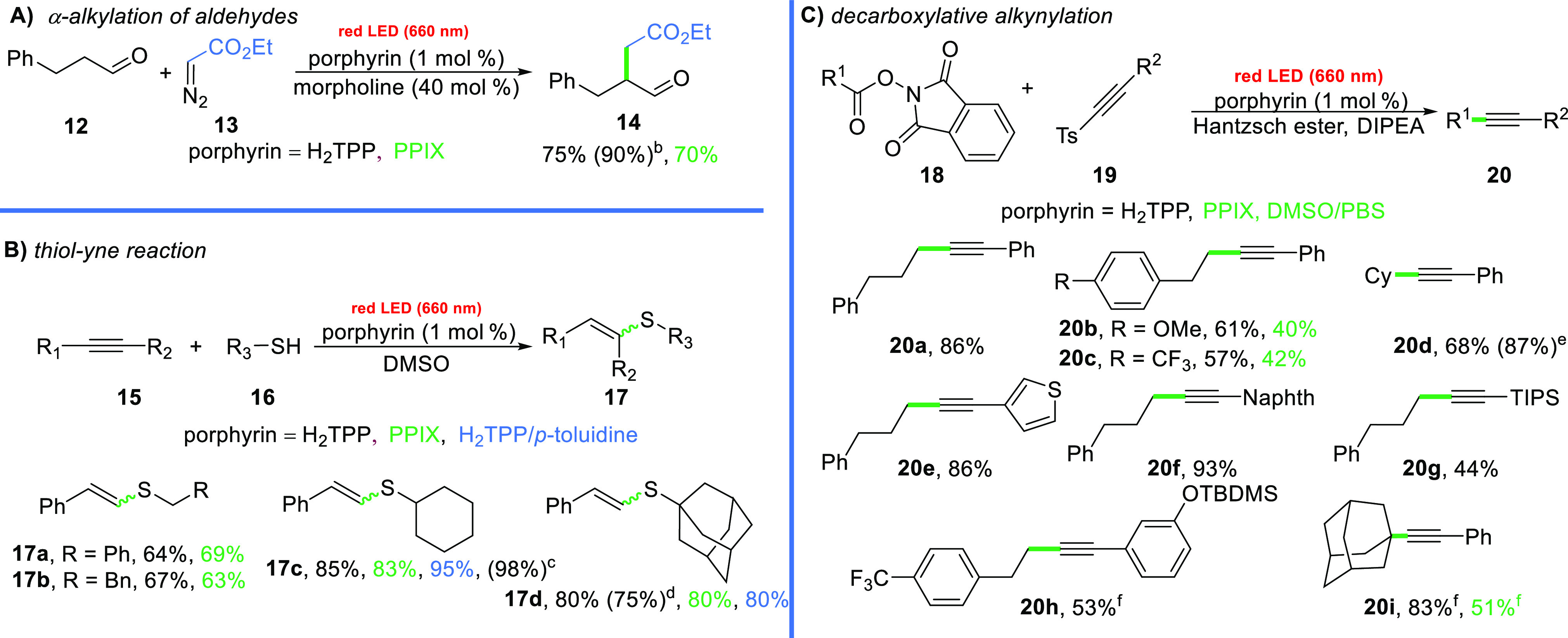
Red-Light-Induced Reaction via Reductive Quenching Reaction conditions are as follows: (A) aldehyde (0.1 mmol, 1 equiv), diazo compound (0.1 mmol, 1 equiv) and H_2_TPP or PPIX (1 mol %) in DMSO/buffer (pH 4) (9:1, 2 mL) under red LED irradiation for 8 h; (B) thiol (0.1 mmol, 1 equiv), alkyne (0.2 mmol, 2 equiv), and porphyrin catalyst (1 mol %) in DMSO (1 mL) under red LED irradiation (660 nm) for 1–8 h; and (C) ester (0.2 mmol, 1 equiv), alkynyl *p*-tolylsulfone (0.2 mmol, 1 equiv), H_2_TPP or PPIX (1 mol %), Hantzsch ester (0.3 mmol, 1.5 equiv), and DIPEA (0.4 mmol, 2 equiv) in acetone or DMSO/PBS (4:1) (2 mL) under red irradiation for 1 h. Blue LED irradiation was used. Blue LED irradiation and Ru(bpy)Cl_2_ were used (see ref (20)). The reaction was performed on a 1 mmol scale. Blue LED irradiation and Ru(bpy)Cl_2_ were used (see ref (22)). 1.1 equiv of the corresponding alkynyl *p*-tolylsulfone was used.

In the next step, the ability of free-base porphyrins to act as photo-oxidants in red-light-induced transformations was evaluated (Scheme 2). Gratifyingly, red-light was as effective as blue-light in inducing the model reaction of 3-phenylpropanal (**12**) with ethyl diazoacetate (**13**), giving product **14** in a 75% yield (Scheme 2A).^29^

With this proof, we turned our attention to reactions suitable for the functionalization of biomolecules. In this context, the cysteine moiety is often regarded as the first choice due to its low natural abundance in peptides and proteins and the high nucleophilicity of the thiol group, and several conjugation methods based on photoredox-based technologies have recently been developed.^30−35^ Such transformations under red-light irradiation have the potential to become practical tools in bio-orthogonal chemistry for even in vivo experiments. With this in mind, we evaluated the activity of porphyrins in photocatalytic additions of thiols to alkenes and alkynes that involved the thiyl radical as the key reactive intermediate.^30−35^ The thiol–yne model reaction of cyclohexanethiol (**16c**) with phenylacetylene (**15a**) in the presence of 1 mol % H_2_TPP (**1**) under red LED irradiation gave the desired product **17c** in a 45% yield (Scheme 2B). After a short optimization, the reaction yield eventually increased to 83% (see the SI). The use of *p*-toluidine as a cocatalyst has a beneficial effect (95% for cyclohexanethiol). With the optimized conditions in hand, we tested the performances of other substrates. Primary thiols, such as 2-phenylethanethiol **16b** and benzyl mercaptan **16a**, reacted efficiently to afford the thiolated products in good yields (67 and 63%, respectively, for the H_2_TPP-catalyzed transformation). Higher yields were obtained for secondary and tertiary thiols, such as cyclohexyl **16c** and adamantane mercaptan **16d**, respectively. Since red-light penetrates deeper, as expected, the scalability of the reaction did not cause any problems (1 mmol scale for adamantane mercaptan, 75% yield).

Furthermore, one of the most advanced photochemical tools developed so far is based on a decarboxylative strategy. It involves the generation of carbon-centered radicals via SET followed by CO_2_ extrusion.^36−38^ Since this approach has already been utilized in transformations of biomolecules,^39^ we wondered whether free-base porphyrins would be able to promote such transformations under red-light irradiation. To this end, the reaction of *N*-hydroxyphthalimide esters (NHPI) with alkynyl *p*-tolylsulfones in the presence of H_2_TPP (**1**) was irradiated with red light. The corresponding internal alkyne **20a** was formed in a 40% yield. By optimizing the reaction parameters, involving different solvents, irradiation powers, and photocatalysts, we found that the reaction could be successfully performed using H_2_TPP in acetone with the addition of DIPEA and a Hantzsch ester as the reductants (Scheme 2C, see the SI for the details). Importantly, the reactivity of the system was retained when conditions resembling a more biological environment, specifically PPIX (**3**) as the photocatalyst and a mixture of DMSO and phosphate-buffered saline (PBS) as the solvent, were used. Under these conditions, variety of *N*-hydroxyphthalimide esters **19** and alkynyl *p*-tolylsulfones **18** were compatible with the red-light-induced decarboxylative coupling.

## Functionalization of Biologically Relevant Molecules

**Figure 2:**
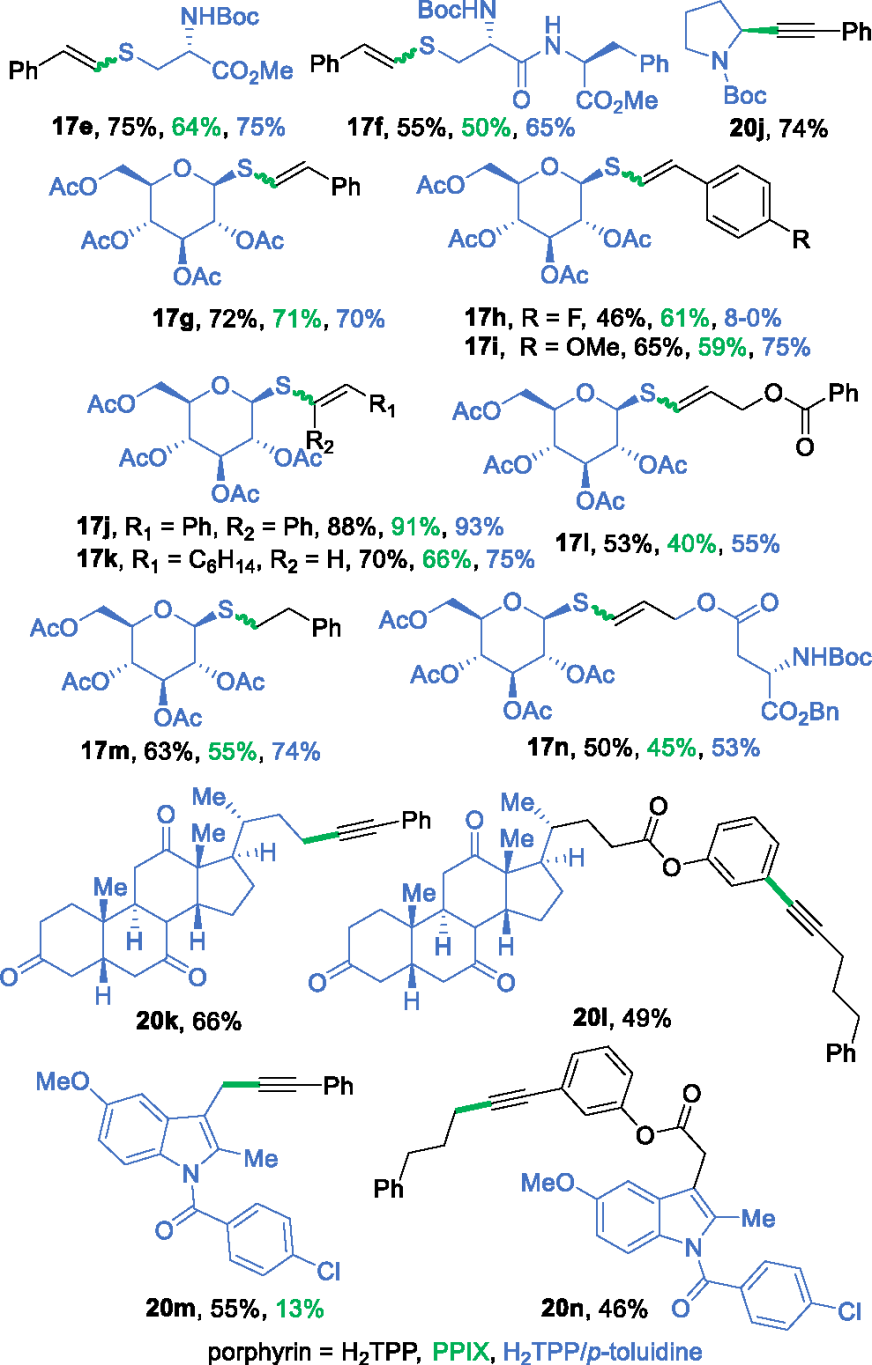
Functionalization of biologically relevant molecules.

The efficacy of porphyrins in red-light-induced C–X bond-forming reactions was further confirmed with biologically relevant molecules (Figure 2). Cysteine **16e** and dipeptide **16f** reacted smoothly in the thiol–yne reaction (75 and 55% yields, respectively, for the H_2_TPP (**1**) catalyzed transformation). Additionally, the addition of 1-thio-α-d-glucose tetraacetate to either phenylacetylene **15a** or styrene **15m** produced high yields (72 or 74%, respectively, in the presence of H_2_TPP). Various phenylacetylenes can be used as starting materials in this transformation. Furthermore, the developed decarboxylative alkynylation protocol is suitable for late-stage functionalizations of complex biomolecules; derivatives of deoxycholic acid and indomethacin furnished the corresponding products **20k**–**20n** in good yields.

In summary, porphyrins are well-known photoredox catalysts under blue- and green-light irradiation. However, due to their versatile photophysical properties, they also promote photoinduced electron transfer processes when exposed to red -light irradiation. They act as efficient photooxidants (e.g., in the alkylation of carbonyl compounds, the thiol–yne reaction, and reductive decarboxylation) and photoreductants (e.g., in the arylation of heteroarenes, selenylation, thiolation, and the reduction of nitro compounds). These bioinspired photocatalysts exhibit features superior to those of other catalysts that work under red-light irradiation, since they are truly nontoxic and can be applied in biological systems. Thus, we believe that free-base porphyrins are valuable additions to the red-light photocatalyst library and that this study will lead to more practical biosynthetic applications.

## Supporting Information Available

Optimization details, experimental procedures, and characterization data for all new compounds (PDF)
