# Supplementary material for: Porphyrins as Promising Photocatalysts for Red-Light-Induced Functionalizations of Biomolecules
Source: ACS Org Inorg Au. 2022 Jul 15;2(5):422–6. doi: 10.1021/acsorginorgau.2c00025 (PMC9955257; doi:10.1021/acsorginorgau.2c00025)
Supplement: Supplementary file 1 — gg2c00025_si_001.pdf [file gg2c00025_si_001.pdf]

Supporting Information

Porphyrins as Promising Photocatalysts for Red-Light Induced Functionalizations of Biomolecules

Katarzyna Rybicka-Jasińska,* Tomasz Wdowik, Klaudia Łuczak,
Aleksandra J. Wierzba, Olga Drapała, Dorota Gryko*

Institute of Organic Chemistry Polish Academy of Sciences, Kasprzaka 01-224 Warsaw, Poland

e-mail: katarzyna.rybickajasinska@icho.edu.pl
dorota.gryko@icho.edu.pl

Table of Contents

1. General Information 3

2. Photoreactor Setups 4

3. Porphyrins as photoreductants in red-light catalysis – oxidative quenching catalytic cycles .5

3.1 Arylation reactions 5

3.2 Photocatalytic hydrogenation of nitrobenzenes 8

4. Porphyrins as photooxidants red-light catalysis – reductive quenching catalytic cycles.......10

4.1 α-Functionalization of aldehydes with diazo esters 10

4.2. Photocatalytic thiol-ene reaction 10

4.3. Reductive decarboxylative C(sp^3)–C(sp) bond coupling reaction 20

5. NMR SPECTRA 31

5.1. *N*-(4-aminophenyl)-2-propylpentanamide (**11**) 311

5.2. ((*1s,3s*)-adamantan-1-yl)(styryl)sulfane (**17d**) (two diastereoisomers) 32

5.3. methyl *N*-(*tert*-butoxycarbonyl)-*S*-styryl-*L*-cysteinyl-*L*-phenylalaninate (**17f**).......................333

5.4. (2*R*,3*R*,4*S*,5*R*,6*S*)-2-(acetoxymethyl)-6-((4-fluorostyryl)thio)tetrahydro-2*H*-pyran-3,4,5-triyl triacetate (**17h**) (two diastereoisomers)..344

5.5. (2*R*,3*R*,4*S*,5*R*,6*S*)-2-(acetoxymethyl)-6-((4-methoxystyryl)thio)tetrahydro-2*H*-pyran-3,4,5-triyl triacetate (**17i**) (two diastereoisomers)...36

5.6. (2*R*,3*R*,4*S*,5*R*,6*S*)-2-(acetoxymethyl)-6-((1,2-diphenylvinyl)thio)tetrahydro-2*H*-pyran-3,4,5-triyl triacetate (**17j**) (two diastereoisomers)...37

5.7. (2*R*,3*R*,4*S*,5*R*,6*S*)-2-(acetoxymethyl)-6-(oct-1-en-1-ylthio)tetrahydro-2*H*-pyran-3,4,5-triyl triacetate (**17k**) (two diastereoisomers)..388

5.8. (2*R*,3*R*,4*S*,5*R*,6*S*)-2-(acetoxymethyl)-6-((3-(benzoyloxy)prop-1-en-1-yl)thio)tetrahydro-2*H*-pyran-3,4,5-triyl triacetate (**17l**) (two diastereoisomers) 39

5.9. 1-benzyl 4-(3-(((2*S*,3*R*,4*S*,5*R*,6*R*)-3,4,5-triacetoxy-6-(acetoxymethyl)tetrahydro-2*H*-pyran-2-yl)thio)allyl) (*tert*-butoxycarbonyl)-*L*-aspartate (**17n**) (two diastereoisomers)..............................40

5.10. 1-(4-phenylbut-3-yn-1-yl)-4-(trifluoromethyl)benzene (**20c**) 41

5.11. 1-(5-phenylpent-1-yn-1-yl)naphthalene (**20f**) 43

5.12. *tert*-butyldimethyl(3-(4-(4-(trifluoromethyl)phenyl)but-1-yn-1-yl)phenoxy)silane (**20h**).......44

5.13. 3-(5-phenylpent-1-yn-1-yl)phenyl (4*R*)-4-((5*S*,9*S*,10*S*,13*R*,14*R*,17*R*)-10,13-dimethyl-3,7,12-trioxohexadecahydro-1*H*-cyclopenta[a]phenanthren-17-yl)pentanoate (**20l**)...............................46

5.14. 3-(5-phenylpent-1-yn-1-yl)phenyl 2-(1-(4-chlorobenzoyl)-5-methoxy-2-methyl-1*H*-indol-3-yl)acetate (**20n**) 47

1. General Information

Materials

All solvents and commercially available reagents were purchased from Sigma-Aldrich, TCI, or Acros Organics as reagent grade and were used without further purification, unless otherwise stated. Porphyrins were purchased from PorphyChem. Dry solvents were taken from Solvent Purification System (SPS) or purchased from Sigma Aldrich. All deuterated solvents used were purchased from Eurisotop.

General Procedures

All the photochemical reactions were performed in 10 mL glassy vials sealed with aluminum caps containing a rubber septum. Reactions were monitored by thin layer chromatography (TLC), using 0.20 mm Merck silica plates (60F-254) and visualized using UV-light, potassium permanganate, cerium molybdate, or anisaldehyde stain, with heat as a developing agent. Chromatography columns were performed on Merck silica gel 60 (230-400 mesh). GC yields were calibrated with dodecane as an internal standard. Isolated yields refer to spectroscopically (^{1}H NMR) homogeneous materials.

Instrumentation

NMR spectra were recorded at ambient temperature (unless otherwise stated) on Bruker 400 or 500 MHz and Varian 500 or 600 MHz. Chemical shifts are reported in ppm relative to the tetramethyl silane signal or solvent peak (TMS: 0 ppm for ^{1}H and ^{13}C, $CHCl_3$: 7.26 ppm for ^{1}H and 77.00 ppm for ^{13}C). Multiplicities are given as: singlet (s), doublet (d), triplet (t), quartet (q), pentet (p), multiplet (m), broad singlet (brs).

LR and HRMS Low-resolution mass spectra (LRMS) were recorded on an Applied Biosystems API 365 mass spectrometer using electrospray ionization (ESI) technique. High-resolution mass spectra (HRMS) were recorded on Waters SYNAPT G2-S HDMS instrument using electron ionization (EI), electrospray ionization (ESI), or atmospheric-pressure chemical ionization (APCI) with time of flight detector (TOF).

Elemental analyses (C, H, N) were performed using a PERKIN-ELMER 240 Elemental Analyzer.

GC-MS analyses were performed using Shimadzu GCMS-QP2010 SE gas chromatograph with FID detector and Zebron ZB 5MSi column.

HPLC analyses were performed using KNAUER, Eurospher II 100-10 Si, 250 x 4 mm column with precolumn, using UV-Vis detection (wavelength: 270 nm) at room temperature. Flow rate: 1mL/min, elution: hexane/ethyl acetate.

2. Photoreactor Setups

Light-mediated reactions were carried out in UOSlab Miniphoto photoreactor. Red (maximum at 660 nm) light was supplied to each reaction vial with the use of 7 LUMINUS LED units (of overall 25 W intensity when 100% power applied). The ambient temperature of LED block was maintained by cooling with Huber MiniChiller 300.

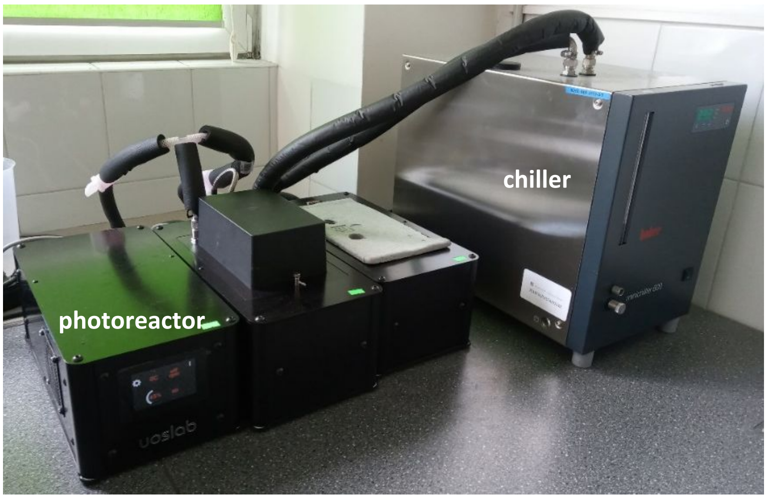

Figure S1. Standard photoreactor setup with chiller.

3. Porphyrins as photoreductants in red-light catalysis – oxidative quenching catalytic cycles

3.1 Arylation reactions

General procedure for arylation of furan:[1]

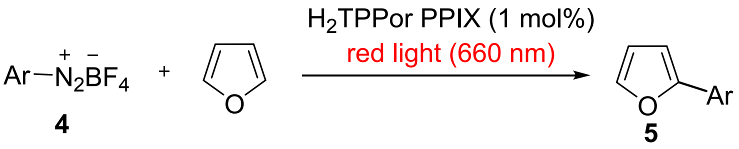

A porphyrin (1 mol%, a) H_2TPP_F (**2**) or b) PPIX (**3**)) and a diazonium salt (0.2 mmol, 1 equiv.) were placed in a 10 mL vial with a septum, dissolved in dry DMSO (1 mL), degassed followed by the addition of furan (10 equiv.). The reaction mixture was stirred under light irradiation (red light, 100% power, 15 °C) for 16 h under argon atmosphere. The light was turned off, the reaction mixture was diluted with AcOEt, and washed with water. The aqueous phase was extracted with AcOEt three times. The combined organic phases were washed with water and brine, dried over Na_2SO_4, filtered, and concentrated. The crude product was purified by flash chromatography using silica gel (hexane/AcOEt).

2-(4-bromophenyl)furan (5)

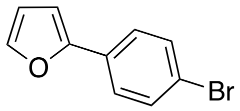

The crude product was purified by column chromatography using silica gel (hexane/AcOEt) to afford 26 mg of compound **5** as white solid, yield = **60%** for H_2TPP_F and **60%** for PPIX, 16 h. Analytical data for compound **5** are in agreement with the literature data.[2]
^{1}H NMR (400 MHz, $CDCl_3$) δ = 7.54-7.46 (m, 5H), 6.65-6.64 (dd, J = 3.4 Hz, J = 0.8 Hz, 1H), 6.47-6.46 (dd, J = 3.4 Hz, J = 1.8 Hz, 1H) ppm.
^{13}C{H} NMR (100 MHz, $CDCl_3$) δ = 153.0, 142.4, 131.8, 129.8, 125.3, 124.1, 111.8, 105.5 ppm.

General procedure for arylation of coumarine:[1]

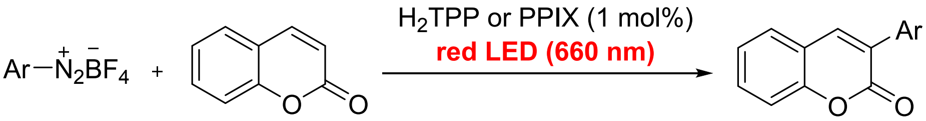

A photocatalyst (1 mol%, a) H_2TPP_F (**2**) or b) PPIX (**3**)), diazonium salt (0.2 mmol, 1 equiv.) coumarine (5 equiv.) were placed in a 10 mL vial with a septum, dissolved in dry DMSO (2 mL) and degassed for 10 min. The reaction mixture was stirred under light irradiation (red light, 100% power, 15 °C) for 17 h under argon atmosphere. The light was turned off, the reaction mixture was diluted with AcOEt, and washed with water. The aqueous phase was extracted with AcOEt three times. The combined organic phases were washed with water and brine, dried over Na_2SO_4, filtered, and concentrated. The crude product was purified by flash chromatography using silica gel (hexane/AcOEt).

---

[1] Modified procedure from: Rybicka-Jasińska, K.; König, B.; Gryko, D. *Eur. J. Org. Chem.,* **2016**, 2104−2107.
[2] Hari, D. P.; Schroll, P.; König, B. *J. Am. Chem. Soc.* **2012**, *134*, 2958−2961.

3-(4-bromophenyl)-2H-chromen-2-one (6)

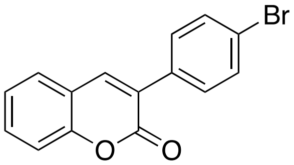

The crude product was purified by column chromatography using silica gel (hexane/AcOEt) to afford 36 mg of compound **6** as white solid, yield = **60%** for H_2TPP_F and **35%** (20 mg) for PPIX, 17 h. Analytical data for compound **6** are in agreement with the literature data.[3]

^{1}H NMR (400 MHz, $CDCl_3$) δ = 7.81 (s, 1H), 7.61-7.52 (m, 6H), 7.39-7.36 (m, 1H), 7.32-2.28 (m, 1H) ppm.

^{13}C{H} NMR (100 MHz, $CDCl_3$) δ = 160.2, 153.6, 139.9, 133.6, 131.7, 131.7, 130.1, 127.9, 127.2, 124.6, 123.2, 119.5, 116.5 ppm.

General procedure for arylation of thiol:[4]

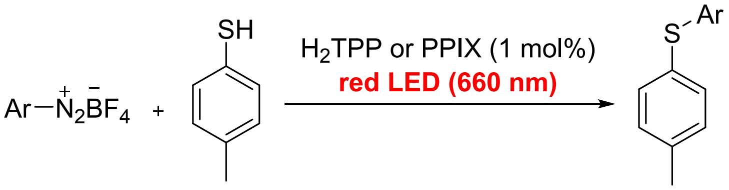

A photocatalyst (1 mol%, a) H_2TPP_F (**2**) or b) PPIX (**3**)), diazonium salt (0.2 mmol, 1 equiv.) were placed in a 10 mL vial with a septum, dissolved in dry DMSO (4 mL), degassed followed by the addition of thiol (1.1 equiv.). The reaction mixture was stirred under light irradiation (red light, 100% power, 15 °C) for 6 h under argon atmosphere. The light was turned off, the reaction mixture was diluted with AcOEt, and washed with water. The aqueous phase was extracted with AcOEt three times. The combined organic phases were washed with water and brine, dried over Na_2SO_4, filtered, and concentrated. The crude product was purified by flash chromatography using silica gel (hexane/AcOEt).

(4-bromophenyl)(p-tolyl)sulfane (7)

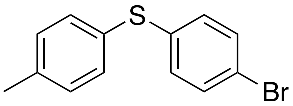

The crude product was purified by column chromatography using silica gel (hexane/AcOEt) to afford 41 mg of compound **7** as white solid, yield = **60%** for H_2TPP_F and **59%** (40 mg) for PPIX, 6 h. Analytical data for compound **7** are in agreement with the literature data.[4]

^{1}H NMR (500 MHz, $CDCl_3$) δ 7.36 (d, J = 8.5 Hz, 2H), 7.30 (d, J = 8.0 Hz, 2H), 7.14 (d, J = 7.8 Hz, 2H), 7.10 (d, J = 9.5 Hz, 2H), 2.35 (s, 3H) ppm.

^{13}C{H} NMR (126 MHz, $CDCl_3$) δ 138.1, 136.8, 132.7, 132.0, 130.9, 130.4, 130.2, 120.1, 21.13 ppm.

[3] Yuan, J.-W.; Yang, L.-R.; Yin, Q.-Y.; Mao, P.; Qu, L.-B. *RSC Adv.* **2016**, *6*, 35936−35944.

[4] Modified procedure from: Bottecchia, C.; Rubens, M.; Gunnoo, S. B.; Hessel, V.; Madder, A; Noël, T. *Angew. Chem. Int. Ed.*, **2017**, *56*, 12702−12707.

General procedure for the synthesis of substituted-(phenyl)selene or substituted-(phenyl)sulfane:[5]

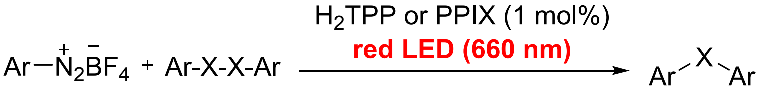

A photocatalyst (1 mol%, a) H_2TPP_F (**2**) or b) PPIX (**3**)), diazonium salt (**4**, 0.2 mmol, 1 equiv.) were placed in a 10 mL vial with a septum, dissolved in dry DMSO (2 mL), degassed followed by the addition of diphenyl diselenide (2 equiv.) or diphenyl disulfide (2 equiv.). The reaction mixture was stirred under light irradiation (red light, 100% power, 15 °C) for 6 h under argon atmosphere. The light was turned off, the reaction mixture was diluted with AcOEt, and washed with water. The aqueous phase was extracted with AcOEt three times. The combined organic phases were washed with water and brine, dried over Na_2SO_4, filtered, and concentrated. The crude product was purified by flash chromatography using silica gel (hexane/AcOEt).

***(4-bromophenyl)(p-tolyl)sulfane** (7)*

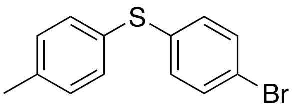

The crude product was purified by column chromatography using silica gel (hexane/AcOEt) to afford 41 mg of compound **7** as white solid, yield = **74%** for H_2TPP_F and **24%** (43 mg) for PPIX, 6 h. Analytical data for compound **7** are in agreement with the literature data.[4]

(4-bromophenyl)(phenyl)selane (8)

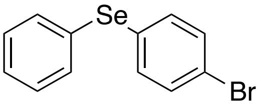

The crude product was purified by column chromatography using silica gel (hexane/AcOEt) to afford 53 mg of compound **8** as colorless oil, yield = **88%** for H_2TPP_F and **81%** (48 mg) for PPIX, 6 h. Analytical data for compound **8** are in agreement with the literature data.[4]
^{1}H NMR (500 MHz, $CDCl_3$) δ = δ 7.50 – 7.42 (m, 2H), 7.40 – 7.32 (m, 2H), 7.32 – 7.24 (m, 5H) ppm.
^{13}C{H} NMR (126 MHz, $CDCl_3$) δ 134.2, 133.3, 132.4, 130.4, 130.4, 129.5, 127.7, 121.5 ppm.

General procedure oxidant- and base-free Porphyrin/Pd catalyzed C–H arylation of anilides:[6]

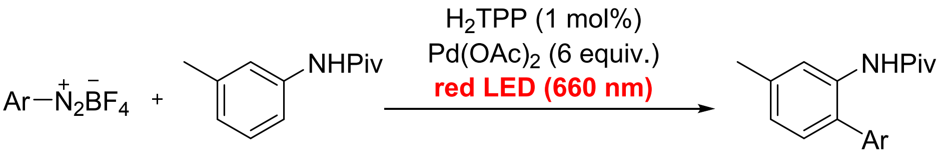

A vial with a septum was charged with a photocatalyst (1 mol%, a) H_2TPP_F (**1**) or b) PPIX (**3**)), pivalamid (0.2 mmol, 1 equiv.), aryldiazonium salt **4** (0.3 mmol), $Pd(OAc)_2$ (10 mol%) and methanol (1 mL) under argon atmosphere. Then the reaction tube was freezed in liquid N_2, degassed by the freeze-pump-thaw procedure (3x), refilled with argon gas. The reaction mixture was stirred under light irradiation (red light, 100% power, 15 °C) for 16 h under argon atmosphere. The light was turned off, the reaction mixture was diluted with AcOEt, and washed with water. The aqueous phase was extracted with AcOEt three times. The combined organic phases were washed with water and brine, dried over

[5] Modified procedure from: Mandal, T.; Das, S.; De Sarkar, S., *Adv. Synth. Catal.*, **2019**, *361*, 3200−3209.

[6] Modified procedure from: Sahoo, M. K.; Midya, S. P.; Landge, V. G.; Balaraman, E. *Green Chem.* **2017**, *19*, 2111−2117.

Na_2SO_4, filtered, and concentrated. The crude product was purified by flash chromatography using silica gel (hexane/AcOEt).

N-(4'-bromo-4-methyl-[1,1'-biphenyl]-2-yl)pivalamide (9)

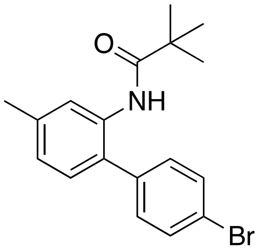

The crude product was purified by column chromatography using silica gel (hexane/AcOEt) to afford 62 mg of compound **9** as white solid, yield = **89%** for H_2TPP_F and **78%** (54 mg) for PPIX, 16 h. Analytical data for compound **9** are in agreement with the literature data.[6]
^{1}H NMR (500 MHz, $CDCl_3$) δ 8.16 – 8.12 (m, 1H), 7.62 – 7.57 (m, 2H), 7.31 (s, 1H), 7.24 – 7.18 (m, 2H), 7.09 (d, J = 7.7 Hz, 1H), 7.02 – 6.94 (m, 1H), 2.39 (s, 3H), 1.13 (s, 9H) ppm.
^{13}C{H} NMR (126 MHz, $CDCl_3$) δ 176.3, 138.9, 137.2, 134.6, 132.1, 131.1, 129.5, 128.5, 125.0, 122.2, 122.0, 39.8, 27.4, 21.4 ppm.

3.2 Photocatalytic hydrogenation of nitrobenzenes[7]

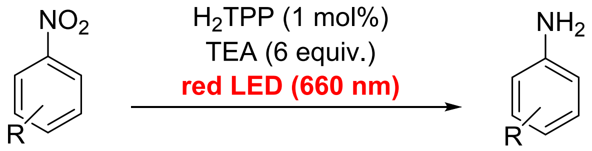

General Procedure:[7] A vial with a septum was charged with a photocatalyst (H_2TPP, 1 mol%), nitrobenzene (0.2 mmol, 1 equiv.), EtOH (3 mL), water (2 mL) and trietanoloamine (6 equiv.). The pH value of the solution was adjusted to 8.50 using concentrated HCl solution. Before irradiation, the sample was deaerated by bubbling argon for 10 min. The reaction mixture was stirred under light irradiation (red light, 100% power, 15 °C) for 24 h under argon atmosphere. The light was turned off, the reaction mixture was diluted with AcOEt, and washed with water. The aqueous phase was extracted with AcOEt three times. The combined organic phases were washed with water and brine, dried over Na_2SO_4, filtered, and concentrated. The crude product was purified by flash chromatography using silica gel (hexane/AcOEt).

Aniline (10)

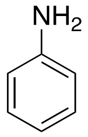

The crude product was purified by column chromatography using silica gel (hexane/AcOEt) to afford 14 mg of compound **10** as yellow oil, yield = **75%** for H_2TPP, 24 h. Analytical data for compound **10** are in agreement with the literature data.[8]
^{1}H NMR (400 MHz, $CDCl_3$) δ 7.24 – 7.16 (m, 2H), 6.81 (tt, J = 7.3, 1.1 Hz, 1H), 6.76 – 6.67 (m, 2H), 3.65 (s, 2H) ppm.
^{13}C{H} NMR (100 MHz, $CDCl_3$) δ 146.5, 129.3, 118.6, 115.2 ppm.

---

[7] Modified procedure from: Yang, X.-J.; Chen, B.; Wu, L.-Q.; Tung, C.-H. *Green Chem.* **2014**, *16*, 1082−1086.

[8] Redwan, I. N.; GrØtli, M. *J. Org. Chem.* **2012**, *77*, 7071−7075.

N-(4-aminophenyl)-2-propylpentanamide (11)

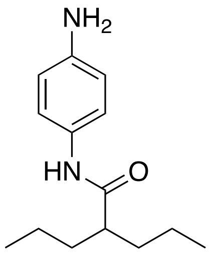

The crude product was purified by column chromatography using silica gel (hexane/AcOEt) to afford 46 mg of compound **11** as white solid, yield = **50%** for H_2TPP, 24 h.

^{1}H NMR (400 MHz, $CDCl_3$) δ 7.34 – 7.21 (m, 2H), 7.03 (s, 1H), 6.72 – 6.57 (m, 2H), 3.68 – 3.50 (m, 2H), 2.14 (m, 1H), 1.68 (m, 3H), 1.55 – 1.22 (m, 7H), 0.91 (t, J = 7.2 Hz, 7H) ppm.

^{13}C{H} NMR (100 MHz, $CDCl_3$) δ 174.1, 143.2, 129.3, 122.0, 115.3, 48.6, 35.4, 20.9, 14.1 ppm.

HRMS (ESI): m/z calcd for $[C_{14}H_{22}N_2O_5 + H]^+$: 235.1810 $[M{+}H]^+$; found 235.1814.

IR (film, cm^{-1}): 3273, 2955, 1650, 1531, 1254.

Anal. Calcd for $C_{14}H_{22}N_2O$: C, 71.76; H 9.46. Found: C, 71.71; H 9.43.

4. Porphyrins as photooxidants red-light catalysis – reductive quenching catalytic cycles

4.1 α-Functionalization of aldehydes with diazo esters

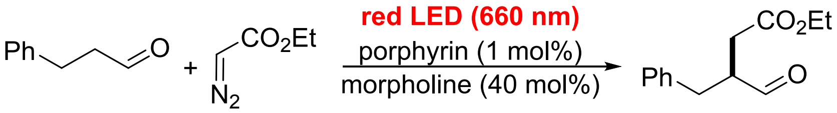

12 **13** **14**

General procedure:[9] A photocatalyst (H_2TPP, 1 mol%) was placed in a 10 mL vial with a septum and dissolved in a mixture of DMSO and buffer PBS = 4 (mixture 1.3:0.3, 1.6 mL) then an aldehyde (0.15 mmol, 1.25 equiv.), piperazine (0.4 equiv., 0.04 mmol) and EDA (0.1 mmol, 1 equiv.) were added. The reaction mixture was stirred under light irradiation (red light, 50% power, 15 °C) for 16 h. The light was turned off, the reaction mixture was diluted with AcOEt, and washed with 1M HCl. The aqueous phase was extracted with AcOEt three times. The combined organic phases were washed with saturated $NaHCO_{3aq}$, brine, dried over Na_2SO_4, filtered, and concentrated. The crude product was purified by flash chromatography using silica gel (hexanes/AcOEt).

Ethyl 3-benzyl-4-oxobutanoate (14)

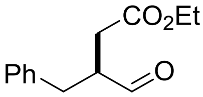

Following the general procedure: compound **14** was obtained from 3-phenylpropanal (**12**, 0.1 mmol) and ethyl diazoacetate (**13**, 0.2 mmol). The crude product was purified by column chromatography using silica gel (hexane/AcOEt) to afford 16 mg of compound **14** as colorless oil, yield = **75%** for H_2TPP and **70%** (14 mg) for PPIX, 16 h). Analytical data for compound **14** are in agreement with the literature data.[9]

^{1}H NMR (500 MHz, $CDCl_3$) δ 9.79 (s, 1H), 7.31 – 7.17 (m, 5H), 4.11 (q, J = 7.1 Hz, 2H), 3.14 – 3.08 (m, 2H), 2.77 – 2.71 (m, 1H), 2.65 (dd, J = 7.6 Hz, 1H), 1.40 (dd, J = 4.8 Hz, 1H), 1.23 (t, J = 7.0 Hz, 3H) ppm.

^{13}C{H} NMR (100 MHz, $CDCl_3$) δ 202.2, 171.6, 137.7, 129.0, 128.6, 126.7, 60.7, 49.2, 34.6, 32.7,14.1 ppm.

4.2. Photocatalytic thiol-ene reaction

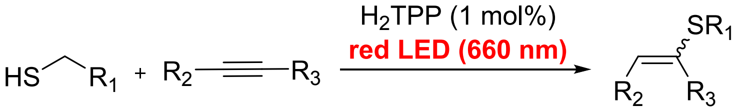

General procedure: A photocatalyst (H_2TPP or PPIX, 1 mol%) was placed in a 10 mLvial with a septum and dissolved in DMSO then thiol (0.1 mmol, 1 equiv.) and alkyne (0.2 mmol, 2 equiv.) were added. The reaction mixture was stirred under light irradiation (red light, 100% power, 15 °C) for specific amount of time. The light was turned off, the reaction mixture was diluted with AcOEt, and washed with water. The aqueous phase was extracted with AcOEt three times. The combined organic phases were washed with water and brine, dried over Na_2SO_4, filtered, and concentrated. The crude product was purified by flash chromatography using silica gel (hexane/AcOEt).

[9] Modified procedure from: Rybicka-Jasińska, K.; Shan, W.; Zawada, K.; Kadish, K. M.; Gryko, D. *J. Am. Chem. Soc.* 2016, *138*, 15451−15458.

Optimization studies:

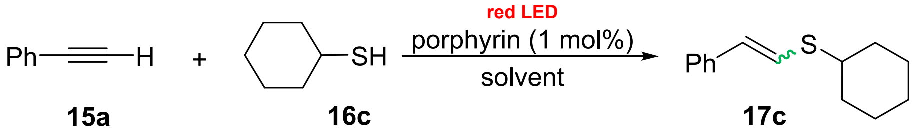

15a **16c** **17c**

Table S1. Background reactions.

Entry	Change of Conditions	Yield [%]*
1	-	45
2	degassed with Ar	26
3	no light	11
4	no photocatalyst	13
5	no light, no photocatalyst	<5

Reaction conditions: thiol (**16c**, 0.2 mmol, 1.0 equiv.), alkyne (**15a**, 1.0 equiv.), photocatalyst: H_2TPP, MeOH:DCE (v/v:2:1, 1 mL), air atmosphere, 16 h, LED_{red} (3 W). *GC yields

Table S2. Optimization of the substrates' ratio.

Entry	thiol [1, equiv.] : alkyne [2, equiv.]	Yield [%]
1	1.0 : 1.0	46
2	1.0 : 1.2	56
3	1.0 : 1.5	59
4	1.0 : 2.0	66
5	1.2 : 1.0	60
6	1.5 : 1.0	49
7	2.0 : 1.0	48

Reaction conditions: thiol (**16c**, xx equiv.), alkyne (**15a**, xx equiv.), photocatalyst: H_2TPP (1 mol%), MeOH:DCE (v/v:2:1, 1 mL), air atmosphere, 16 h, LED_{red} (3 W). *GC yields

Table S3. Optimization of a solvent.

Entry	Solvent	Yield [%]
1	MeOH	44
2	DCE	42
3	DCM	59
4	THF	71
5	DMF	56
6	EtOH	59
7	DMSO	69

Reaction conditions: thiol (**16c**, 0.2 mmol, 1.0 equiv.), alkyne (**15a**, 2.0 equiv.), photocatalyst: H_2TPP (1 mol%), solvent (1 mL), air atmosphere, 16 h, LED_{red} (3 W). *GC yields

Table S4. Optimization of time vs LED power.

Entry	time	LED power	Yield [%]*
1	16 h	3 W	71
2	1 h	25 W	76
**3**	1 h	25 W	85**

Reaction conditions: thiol (**1**, 0.2 mmol, 1.0 equiv.), alkyne (**2**, 2.0 equiv.), photocatalyst: H_2TPP (1 mol%), THF (1 mL), air atmosphere, xx h, LED_{red} (x W). *GC yields ** DMSO instead of THF was used

Table S5. Optimization of photocatalyst.

Entry	Porphyrin	Yield [%]*
1	H_2TPP	85
**2**	H_2TP(*p*-OMe)PP	77
**3**	H_2TP(*p*-OH)PP	81
2	PPIX	83
**3**	Zn-PPIX	77

Reaction conditions: thiol (**16c**, 0.2 mmol, 1.0 equiv.), alkyne (**15a**, 2.0 equiv.), photocatalyst: porphyrin (1 mol%), DMSO (1 mL), air atmosphere, 1 h, LED_{red} (40W). *GC yields

Table S6. Influence of co-catalyst.

Entry	co-catalyst	Additive [equiv.]	Yield [%]*
1	*p*-toluidine	0.5	<95
2	*p*-toluidine	0.4	94
3	*p*-toluidine	0.2	75
2	*p*-toluidine	0.5	<95**

Reaction conditions: thiol (**16c**, 0.2 mmol, 1.0 equiv.), alkyne (**15a**, 1.0 equiv.), photocatalyst: PPIX (1 mol%), DMSO (1 mL), air atmosphere, 1 h, LED_{red} (25 W). *GC yields ** H_2TPP was used as photocatalyst.

Scope and limitations studies:

Benzyl(styryl)sulfane (17a) *(two diastereoisomers)*

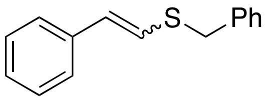

Following the general procedure, compound **17a** was obtained from phenylacetylene (0.2 mmol) and benzylthiol (0.1 mmol). The crude product was purified by column chromatography to using silica gel (hexane/AcOEt) afford 15 mg of compound **17a** as a mixture of two diastereoisomers (*Z*/*E* = 66:34 for H_2TPP) (yellowish oil, yield = **64%** for H_2TPP, yield = **69%** (16 mg) for PPIX, 3 h). Analytical data for compound **17a** are in agreement with the literature data.[10]

^{1}H NMR (500 MHz, $CDCl_3$) δ 7.47 – 7.43 (m, 1H + *E*), 7.41 – 7.31 (m, 6H + *E*), 7.26 (m, 2H + *E*), 7.22 – 7.13 (m, 1H + *E*), 6.71 (d, *J* = 15.5 Hz, 1H), 6.52 (d, *J* = 15.5 Hz, 1H), 6.41 (d, *J* = 10.9 Hz, 1H), 6.24 (d, *J* = 10.9 Hz, 1H), 4.00 (s, 2H), 3.99 (s, 2H) ppm.

^{13}C{H} NMR (126 MHz, $CDCl_3$) δ 137.4, 137.2, 136.9, 136.9, 128.9, 128.8, 128.7, 128.7, 128.7, 128.6, 128.2, 128.0, 127.4, 127.3, 127.0, 126.7, 126.0, 125.9, 125.6, 124.4, 39.5, 37.4 ppm.

Phenethyl(styryl)sulfane (17b) *(two diastereoisomers)*

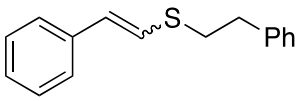

Following the general procedure, compound **17b** was obtained from phenylacetylene (0.2 mmol) and 2-phenylethanethiol (0.1 mmol). The crude product was purified by column chromatography using silica gel (hexane/AcOEt) to afford 16 mg of compound **17b** as a mixture of two diastereoisomers (*Z*/*E* = 63:37 for H_2TPP) (yellowish oil, yield = **67%** for H_2TPP, yield = **63%** (15 mg) for PPIX, 3 h). Analytical data for compound **17b** are in agreement with the literature data.[11]

---

[10] Castoldi, L.; Di Tommaso, E. M.; Gräfen, B.; Olofsson, B.; Reittti, M. *Angew. Chem. Int. Ed.* **2020**, *59*, 15512−15516.

[11] Ranjit, S.; Duan, Z.; Zhang, P.; Liu, X. *Org. Lett.* **2010**, *12*, 4134−4136.

^{1}H NMR (500 MHz, $CDCl_3$) δ 7.50 – 7.44 (m, 1H + *E*), 7.39 – 7.27 (m, 9H), 7.26 – 7.16 (m, 9H), 6.69 (d, *J* = 15.5 Hz, 1H), 6.49 (d, *J* = 15.6 Hz, 1H), 6.46 (d, *J* = 10.8 Hz, 1H), 6.24 (d, *J* = 10.8 Hz, 1H), 3.12 – 2.87 (m, 4H + *E*) ppm.
^{13}C{H} NMR (126 MHz, $CDCl_3$) δ 140.0, 139.8, 137.0, 136.9, 128.6, 128.6, 128.6, 128.6, 128.5, 128.5, 128.2, 127.4, 127.0, 126.9, 126.7, 126.5, 126.5, 126.4, 125.9, 125.5, 124.7, 40.2, 37.1, 36.8, 35.9, 35.7, 34.0 ppm.

***Cyclohexyl(styryl)sulfane** (17c) (two diastereoisomers)*

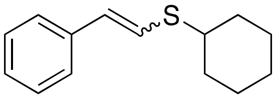

Following the general procedure, compound **17c** was obtained from phenylacetylene (0.2 mmol) and cyclohexanethiol (0.1 mmol). The crude product was purified by column chromatography using silica gel (hexane/AcOEt) to afford 19 mg of compound **17c** as a mixture of two diastereoisomers (*Z*/*E* = 76:24 for H_2TPP) (yellowish oil, yield = **85%** for H_2TPP, yield = **83%** (18 mg) for PPIX, yield **95%** (21 mg) for PPIX/*p*-toluidine, 1 h). Analytical data for compound **17c** are in agreement with the literature data.[12]
^{1}H NMR (500 MHz, $CDCl_3$) δ 7.53 – 7.44 (m, 2H + *E*), 7.34 (t, *J* = 7.8 Hz, 2H + *E*), 7.32 – 7.27 (m, 1H + E), 7.23 – 7.14 (m, 1H), 6.75 (d, *J* = 16.0 Hz, 1H), 6.57 (d, *J* = 15.5 Hz, 1H), 6.42 (d, *J* = 11.0 Hz, 1H), 6.33 (d, *J* = 11.0 Hz, 1H), 2.98 (m, 1H), 2.88 (m, 1H), 2.07 (m, 2H + *E*), 1.80 (m, 2H + *E*), 1.64 (m, 1H + *E*), 1.57 – 1.23 (m, 5H + *E*).
^{13}C{H} NMR (126 MHz, $CDCl_3$) δ 137.2, 137.2, 128.7, 128.7, 128.6, 128.2, 126.9, 126.5, 125.9, 125.6, 125.0, 124.0, 47.8, 45.3, 33.7, 33.6, 26.0, 26.0, 25.7, 25.6 ppm.

***((1s,3s)-adamantan-1-yl)(styryl)sulfane** (17d) (two diastereoisomers)*

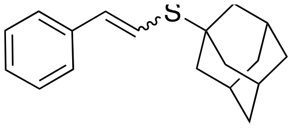

Following the general procedure, compound **17d** was obtained from phenylacetylene (0.2 mmol) and 1-adamantanethiol (0.1 mmol). The crude product was purified by column chromatography using silica gel (hexane/AcOEt) to afford 22 mg of compound **17d** as a mixture of two diastereoisomers (*Z*/*E* = 86:14 for H_2TPP) (white solid, yield = 80**%** (202mg, **75% for 1 mmol scale**) for H_2TPP, yield = **80%** (22 mg) for PPIX, 3 h).
^{1}H NMR (500 MHz, $CDCl_3$) δ 7.53 – 7.45 (m, 2H + *E*), 7.37 – 7.29 (m, 2H + *E*), 7.18 (t, *J* = 7.4 Hz, 1H + *E*), 6.92 (d, *J* = 12.8 Hz, 1H), 6.70 (d, *J* = 15.5 Hz, 1H), 6.50 (d, *J* = 11.2 Hz, 1H), 6.46 (d, *J* = 11.2 Hz, 1H), 2.0.9 (m, 3H + *E*), 1.98 (brs, 6H + *E*), 1.95 (brs, 6H), 1.72 (m, 6H + *E*) ppm.
^{13}C{H} NMR (126 MHz, $CDCl_3$) δ 137.3, 131.9, 128.7, 128.6, 128.1, 127.1, 126.4, 125.8, 125.2, 121.2, 120.2, 46.6, 43.7, 43.3, 36.2, 36.2, 29.8, 29.8 ppm.
HRMS (EI): *m*/*z* calcd for $C_{18}H_{22}S$: 270.1442 [*M*]; found 270.1446.
Anal. Calcd for $C_{18}H_{22}S$: C, 79.94; H, 8.20; S, 11.86. Found: C, 79.95; H, 8.05; S, 12.02. **IR** (film, cm^{-1}): 3020, 2907, 1590, 1442.

***methyl N-(tert-butoxycarbonyl)-S-styryl-L-cysteinate** (17e) (two diastereoisomers)*

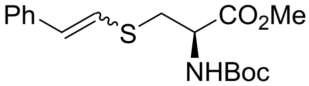

Following the general procedure, compound **17e** was obtained from phenylacetylene (0.2 mmol) and *N*-Boc-*L*-cysteine methyl ester (0.1 mmol). The crude product was purified by column chromatography

[12] Ananikov, V. P.; Orlov, N. V.; Beletskaya, I. P.; Khrustalev, V. N.; Antipin, M. Y.; Timofeeva, T. V. *J. Am. Chem. Soc.* **2007**, *129*, 23, 7252−7253.

using silica gel (hexane/AcOEt) to afford 25 mg of compound **17e** as a mixture of two diastereoisomers (*Z*/*E* = 62:38 for H_2TPP) (yellowish oil, yield = **75%** for H_2TPP, yield = **64%** (21 mg) for PPIX, 5 h). Analytical data for compound **17e** are in agreement with the literature data.[13]
^{1}H NMR (500 MHz, $CDCl_3$) δ 7.44 (m, 2H), 7.34 (m, 3H), 7.28 (m, 3H), 7.21 (m, 2H), 6.62 (d, *J* = 15.5 Hz, 1H), 5.58 (d, *J* = 15.5 Hz, 1H), 6.42 (d, *J* = 11.0 Hz, 1H), 6.15 (d, *J* = 11.0 Hz, 1H), 5.41 (brs, 1H + *E*), 4.03 (brs, 1H + E), 3.29 – 3.22 (m, 2H + *E*) 3.75 (s, 3H), 3.70 (s, 3H), 1.44 (s, 9H), 1.42 (s, 9H) ppm.
^{13}C{H} NMR (126 MHz, $CDCl_3$) δ 170.9, 170.8, 154.9, 154.9, 136.5, 136.5, 129.6, 129.6, 128.6, 128.6, 128.2, 127.3, 126.8, 126.6, 126.3, 125.7, 123.9, 80.2, 80.2, 53.7, 53.5, 52.6, 38.3, 35.7, 28.2, 28.2 ppm.
HRMS (ESI): *m/z* calcd for $C_{17}H_{23}NO_4SNa$: 360.1246 $[M+Na]^+$; found 360.1245.

methyl N-(tert-butoxycarbonyl)-S-styryl-L-cysteinyl-L-phenylalaninate (17f)

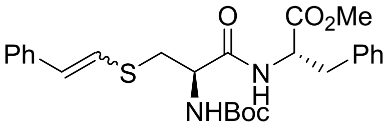

Following the general procedure, compound **17f** was obtained from phenylacetyelene (0.2 mmol) and methyl (*tert*-butoxycarbonyl)cysteinylphenylalaninate[14] (0.1 mmol). The crude product was purified by column chromatography using silica gel (hexane/AcOEt) to afford 27 mg of compound **17f** (white oil, yield = **55%** for H_2TPP, 8 h).
^{1}H NMR (500 MHz, $CDCl_3$) δ 7.43 – 7.38 (m, 2H), 7.36 – 7.31 (m, 2H), 7.31 – 7.26 (m, 2H), 7.24 – 7.15 (m, 4H), 6.71 (d, *J* = 7.2 Hz, 1H), 6.34 (d, *J* = 10.7 Hz, 1H), 6.00 (d, *J* = 10.8 Hz, 1H), 4.90 (s, 1H), 4.83 (dt, *J* = 7.3, 4.7 Hz, 1H), 4.40 (s, 1H), 3.71 (s, 3H), 3.23 (d, *J* = 4.6 Hz, 2H), 3.06 (q, *J* = 6.9, 6.1 Hz, 2H), 1.38 (s, 9H) ppm.
^{13}C{H} NMR (151 MHz, $CDCl_3$) δ 171.0, 170.0, 155.3, 136.4, 129.3, 129.2, 128.73, 128.7, 128.5, 128.5, 128.2, 127.0, 127.0, 126.7, 126.3, 125.7, 80.3, 55.6, 52.7, 52.7, 38.0, 37.8, 28.2 ppm.
HRMS (ESI): *m/z* calcd for $C_{26}H_{32}N_2O_5SNa$: 507.1930 $[M+Na]^+$; found 507.1932.
Anal. Calcd for $C_{26}H_{32}N_2O_5S$: C, 64.4; H, 6.66; S, 6.62; N, 5.78. Found: C, 64.37; H, 6.70; S, 6.53; N, 5.72.
IR (film, cm^{-1}): 3305, 2978, 1744, 1662, 1522, 1249, 1169, 699.

(2R,3R,4S,5R,6S)-2-(acetoxymethyl)-6-(styrylthio)tetrahydro-2H-pyran-3,4,5-triyl triacetate (17g)
(two diastereoisomers)

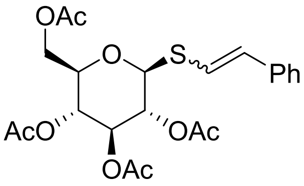

Following the general procedure, compound **17g** was obtained from acetylene (0.2 mmol) and 1-thio-β-D-glucose tetraacetate (0.1 mmol). The crude product was purified by column chromatography using silica gel (hexane/AcOEt) to afford 34 mg of compound **17g** as a mixture of two diastereoisomers (*Z*/*E* = 66:33 for H_2TPP) (yellowish oily foam, yield = **72%** for H_2TPP, yield = **71%** (33 mg) for PPIX, 6 h). Analytical data for compound **17g** are in agreement with the literature data.[15]

---

[13] Di Giuseppe, A.; Castarlenas, R.; Perez-Torrente, J.; Crusianelli, M.; Polo, V.; Sancho, R.; Lahoz, F. J.; Oro, L. A. *J. Am. Chem. Soc.* 2012, *134*, 8171−8183.

[14] Synthesised according to the literature Gutierrez, A.; Marrzo, I.; Cativiela, C.; Laguna, A.; Gimeno, M. C. *Chem.- Eur. J. Chem.* **2015**, *21*, 11088−11095. Analytical data for the compound are in agreement with the literature data.

[15] Brachet, E.; Brion, J.-D.; Alami, M.; Messaoudi, S. *Adv. Synth. Catal.* **2013**, *355*, 2627−2636.

^{1}**H NMR** (500 MHz, $CDCl_3$) δ 7.42 – 7.29 (m, 3H, *Z* + *E*), 7.29 – 7.18 (m, 2H, (*Z* + *E*)), 6.75 (s, 2H), 6.61 (d, *J* = 10.8 Hz, 1H), 6.35 (d, *J* = 10.8 Hz, 1H), 5.24 (td, *J* = 9.3, 4.0 Hz, 1H + *E*), 5.18 – 5.05 (m, 2H + *E*), 4.64 (d, *J* = 9.9 Hz, 1H + *E*), 4.27 (ddd, *J* = 12.9, 8.1, 4.9 Hz, 1H + E), 4.19 – 4.12 (m, 1H + *E*), 3.77 (ddd, *J* = 10.0, 4.8, 2.3 Hz, 1H), 2.10 (s, 3H), 2.08 (s, 3H), 2.06 (s, 3H), 2.03 (s, 3H), 2.02 (s, 3H + *E*), 2.00 (s, 3H), 1.97 (s, 3H) ppm.
^{13}**C{H} NMR** (126 MHz, $CDCl_3$) δ 170.6, 170.6, 170.1, 169.3, 169.1, 136.1, 134.8, 129.1, 128.7, 128.7, 128.3, 127.9, 127.3, 126.2, 120.2, 118.2, 84.4, 83.6, 76.7, 76.3, 76.2, 73.8, 70.0, 69.9, 68.2, 68.1, 62.1, 62.0, 20.7, 20.7, 20.6, 20.6, 20.5 ppm.
HRMS (ESI): *m/z* calcd for $C_{22}H_{26}O_9SNa$: 489.1195 $[M{+}Na]^+$; found 489.1186.

***(2R,3R,4S,5R,6S)-2-(acetoxymethyl)-6-((4-fluorostyryl)thio)tetrahydro-2H-pyran-3,4,5-triyl triacetate (17h)** (two diastereoisomers)*

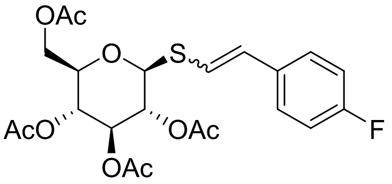

Following the general procedure with a change of stoichiometry: compound **17h** was obtained from 1-ethynyl-4-fluorobenzene (0.2 mmol) and 1-thio-β-D-glucose tetraacetate (0.1 mmol). The crude product was purified by column chromatography using silica gel (hexane/AcOEt) to afford 47 mg of compound **17h** as white oily foam **17h** as a mixture of two diastereoisomers (*Z*/*E* = 75:25), yield = **46%** for H_2TPP, yield = **80%** (77 mg) **(**for H_2TPP/*p*-toluidine, yield = **61%** (59 mg) for PPIX, 16 h).
^{1}**H NMR** (400 MHz, $CDCl_3$) δ 7.38 – 7.32 (m, 2H), 7.32 – 7.26 (m, 2H), 7.09 – 6.94 (m, 2H + *E), 6.72* (d, J = 15.2.5 Hz, 1H), 6.65 (d, *J* = 16.4 Hz, 1H), 6.58 (d, *J* = 10.8 Hz, 1H), 6.32 (d, *J* = 10.8 Hz, 1H), 5.24 (m, 1H + *E*), 5.12 (m, 2H + *E*), 4.64 (d, *J* = 10.8 Hz, 1H), 4.62 (d, *J* = 9.6 Hz, 1H), 4.27 (m, 1H + *E*), 4.15 (m, 1H + *E*), 3.77 (m, 1H + *E*), 2.08 (s, 3H + *E*), 2.06 (s, 3H), 2.04 (s, 3H), 2.02 (s, 3H + *E*), 2.00 (s, 3H +*E*), 1.97 (s, 3H) ppm,
^{13}**C{H} NMR** (126 MHz, $CDCl_3$) δ 170.6, 170.5, 170.1, 169.3, 169.3, 169.2, 169.1, 134.0, 132.5, 132.5, 132.3, 132.2, 128.1, 127.9, 127.8, 119.8, 119.8, 117.7, 117.7, 115.8, 115.5, 115.3, 115.1, 84.2, 83.5, 76.3, 76.2, 73.8, 69.9, 68.2, 68.1, 68.1, 62.1, 62.0, 20.7, 20.7, 20.6, 20.5, 20.5 ppm.
^{19}**F NMR** (470 MHz, $CDCl_3$) δ –113.5 (m), –113. 8 (m).
HRMS (ESI): *m/z* calculated for $C_{22}H_{25}O_8NaSF$: 507.1095, $[M{+}Na]^+$; found = 507.1101.
Anal. Calcd for $C_{22}H_{25}FO_9S$: C, 54.54; H, 5.20. Found: C, 54.46; H, 5.13.
IR (film, cm^{-1}): 2952, 1753, 1225, 1162, 1041.

***(2R,3R,4S,5R,6S)-2-(acetoxymethyl)-6-((4-methoxystyryl)thio)tetrahydro-2H-pyran-3,4,5-triyl triacetate (17i)** (two diastereoisomers)*

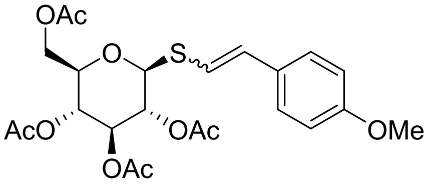

Following the general procedure with a change of stoichiometry: compound **17i** was obtained from *p*-methoxyphenylacetlyene (0.2 mmol) and 1-thio-β-D-glucose tetraacetate (0.1 mmol). The crude product was purified by column chromatography using silica gel (hexane/AcOEt) to afford 36 mg of compound **17i** as white oily foam **17i** as a mixture of two diastereoisomers (*Z*/*E* = 66:33), yield = **65%** for H_2TPP, yield = **75%** (41 mg) for H_2TPP/*p*-toluidine, 6 h).
^{1}**H NMR** (500 MHz, $CDCl_3$) δ 7.38 – 7.32 (m, 2H), 7.32 – 7.24 (m, 2H), 6.91 – 6.83 (m, 2H + *E*), 6.74 (d, *J* = 15.0 Hz, 1H), 6.59 (d, *J* = 15.5 Hz, 1H), 6. 57 (d, *J* =10.5 Hz, 1H), 6.22 (d, *J* = 10.7 Hz, 1H), 5.28 – 5.20 (m, 1H + *E*), 5.14 (m, 2H), 4.64 (d, *J* = 10.0 Hz, 1H), 4.61 (d, *J* = 9.9 Hz, 1H), 4.27 (td, *J* =

12.0, 4.8 Hz, 1H + *E*), 4.22 – 4.11 (m, 1H + *E*), 3.81 (s, 3H + *E*), 3.78 (m, 1H + *E*), 2.09 (s, 3H), 2.07 (s, 3H), 2.05 (s, 3H), 2.03 (s, *J* = 2.4 Hz, 3H + *E*), 2.00 (s, 3H + *E*), 1.98 (s, 3H) ppm.
^{13}C{H} NMR (126 MHz, $CDCl_3$) δ 170.6, 1706, 170.1, 169.3, 169.3, 169.2, 169.1, 159.6, 158.7, 135.8, 130.1, 129.1, 128.9, 128.8, 127.6, 117.6, 115.0, 114.1, 113.7, 84.4, 83.8, 76.8, 76.2, 76.1, 73.9, 73.8, 70.0, 69.9, 68.2, 68.1, 62.1, 62.0, 55.3, 55.2, 20.7, 20.7, 20.6, 20.5, 20.5 ppm.
HRMS (ESI): *m/z* calcd for $C_{23}H_{28}O_{10}SNa$: 519.1301 [*M+Na*]$^+$; found 519.1299
Anal. Calcd for $C_{23}H_{28}O_{10}S$: C, 55.64; H, 5.68. Found: C, 55.35; H, 5.73.
IR (film, cm^{-1}): 2955, 1754, 1606, 1510, 1369, 1227, 1175, 1038.

(2R,3R,4S,5R,6S)-2-(acetoxymethyl)-6-((1,2-diphenylvinyl)thio)tetrahydro-2H-pyran-3,4,5-triyl triacetate (17j) *(two diastereoisomers)*

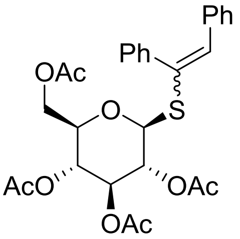

Following the general procedure: compound **17j** was obtained from diphenylacethylene (0.4 mmol) and 1-thio-β-D-glucose tetraacetate (0.2 mmol). The crude product was purified by column chromatography using silica gel (hexane/AcOEt) to afford 96 mg of compound **17j** as a white oily foam **17j** as a mixture of two diastereoisomers (*Z*/*E* = 50:50), yield = **88%** for H_2TPP, yield = **93%** (101 mg) for H_2TPP/*p*-toluidine, yield = **91 %** (99 mg) for PPIX 16 h).
^{1}H NMR (500 MHz, $CDCl_3$ *Z*/*E* 1:1 mixture) δ 7.66 – 7.55 (m, 4H), 7.45 – 7.32 (m, 11H), 7.32 – 7.25 (m, 1H), 7.15 – 7.05 (m, 2H), 7.00 (s, 1H), 6.98 – 6.92 (m, 2H), 6.90 (s, 1H), 5.11 – 4.94 (m, 6H), 4.30 (d, *J* = 4.3 Hz, 1H), 4.28 (d, *J* = 4.0 Hz, 1H), 4.17 (dd, *J* = 12.3, 5.3 Hz, 1H), 4.10 (dd, *J* = 12.3, 4.8 Hz, 1H), 4.01 (dd, *J* = 12.3, 2.2 Hz, 1H), 3.95 (dd, *J* = 12.3, 2.4 Hz, 1H), 3.29 (m, 1H), 3.16 – 3.09 (m, 1H), 2.10 (s, 3H), 2.09 (s, 3H), 2.06 (s, 3H), 2.00 (s, 3H), 1.99 (s, 3H), 1.97 (s, 3H), 1.96 (s, 3H), 1.91 (s, 3H) ppm.
^{13}C{H} NMR (126 MHz, $CDCl_3$) δ 170.5, 170.2, 169.3, 169.3, 169.2, 140.5, 137.1, 136.2, 136.0, 134.2, 134.1, 134.0, 131.7, 129.7, 129.7, 129.1, 128.7, 128.6, 128.5, 128.3, 128.3, 128.1, 128.1, 127.7, 127.2, 83.9, 82.9, 76.8, 75.7, 75.7, 73.9, 73.9, 70.2, 69.8, 68.3, 68.1, 62.1, 61.8, 20.7, 20.7, 20.5, 20.5, 20.5, 20.5, 20.4 ppm.
HRMS (ESI): *m/z* calculated for $C_{28}H_{30}O_9SNa$: 565.1508 [M+Na]$^+$; found 565.1517.
IR (film, cm^{-1}): 3056, 1754, 1224, 1040.
HPLC: >95% purity

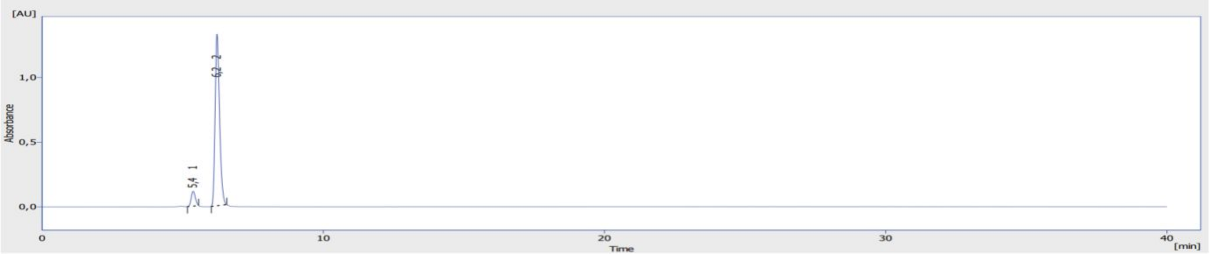

	Reten. Time [min]	Area [mAU.s]	Height [mAU]	Area [%]	Height [%]	W05 [min]	PDA Peak Purity	Compound Name	PDA Best Match Name	PDA Best Match
1	5,367	1145,968	114,627	7,3	8,0	0,18	561			
2	6,217	14574,606	1324,173	92,7	92,0	0,18	835			
	Total	15720,574	1438,799	100,0	100,0					

(2R,3R,4S,5R,6S)-2-(acetoxymethyl)-6-(oct-1-en-1-ylthio)tetrahydro-2H-pyran-3,4,5-triyl triacetate (17k) *(two diastereoisomers)*

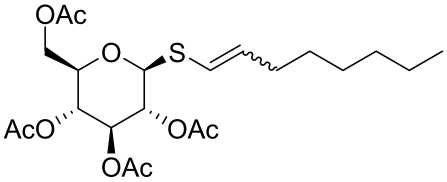

Following the general procedure: compound **17k** was obtained from oct-1-yne (0.4 mmol) and 1-thio-β-D-glucose tetraacetate (0.2 mmol). The crude product was purified by column chromatography using silica gel (hexane/AcOEt) to afford 63 mg of compound **17k** as a white oily foam **17k** as a mixture of two diastereoisomers (*Z/E* 50:50) yield = **65%** for H_2TPP, yield = **75%** (72 mg) for H_2TPP/*p*-toluidine, 12 h, yield = **66%** (64 mg) for PPIX, 16 h).

^{1}H NMR (500 MHz, $CDCl_3$) δ 6.05 (d, *J* = 9.5 Hz, 1H), *5.99 (m, 1H),* 5.94 (m, 1H), *5.80 (m, 1H)*, 5.22 (t, *J* = 9.4 Hz, 2H (*two diastereoisomers*)), 5.13 – 5.03 (m, 4H (*two diastereoisomers*)), 4.52 (d, *J* = 10 Hz, 1H), *4.85 (d, J = 10 Hz, 1H)*, 4.29 – 4.23 (m, 2H (*two diastereoisomers*)), 4.15 (dd, *J* = 5.6, 2.4 Hz, 1H), *4.13 (dd, J = 5.6, 2.3 Hz, 1H),* 3.73 (m, 2H (*two diastereoisomers*)), 2.14 – 2.07 (m, 3H), 2.08 (s, 6H), 2.05 (s, 6H (*two diastereoisomers*)), 2.02 (s, 6H (*two diastereoisomers*)), 2.00 (s, 6H (*two diastereoisomers*)), 1.43 – 1.34 (m, 4H (*two diastereoisomers*)), 1.34 – 1.21 (m, 13H (*two diastereoisomers*)), 0.88 (m, 6H (*two diastereoisomers*)) ppm.

^{13}C{H} NMR (126 MHz, $CDCl_3$) 170.6, 170.2, 169.4, 169.3, 169.1, 169.1, 140.2, 134.7, 118.0, 116.2, 83.8, 83.5, 76.8, 76.1, 76.0, 74.0, 73.9, 70.1, 69.8, 68.3, 68.2, 62.2, 62.1, 33.2, 31.6, 31.6, 29.2, 28.9, 28.8, 28.7, 28.7, 22.6, 20.7, 20.7, 20.6, 20.6, 20.6, 14.0 ppm.

HRMS (ESI): *m/z* calculated for $C_{22}H_{34}O_9SNa$: 497.1821 $[M+Na]^+$; found 497.1823

Anal. Calcd for $C_{22}H_{34}O_9S$: C, 55.68; H, 7.22. Found: C, 55.78; H, 7.38.

IR (film, cm^{-1}): 2928, 1757, 1368, 1227, 1041.

(2R,3R,4S,5R,6S)-2-(acetoxymethyl)-6-((3-(benzoyloxy)prop-1-en-1-yl)thio)tetrahydro-2H-pyran-3,4,5-triyl triacetate (17l) *(two diastereoisomers)*

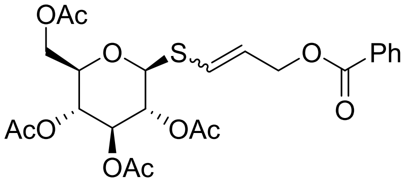

Following the general procedure: compound **17l** was obtained from propargyl benzoate (0.4 mmol) and 1-thio-β-D-glucose tetraacetate (0.2 mmol). The crude product was purified by column chromatography using silica gel (hexane/AcOEt) to afford 52 mg of compound **17l** as a white oily foam **17l** as a mixture of two diastereoisomers (*Z/E* = 50:50), yield = **53%** for H_2TPP, yield = **55%** (53 mg) for H_2TPP/*p*-toluidine, yield = **40%** (39 mg) for PPIX, 16 h).

^{1} H NMR (500 MHz, $CDCl_3$) δ 8.09 – 7.99 (m, 2H + *E*), 7.59 – 7.53 (m, 1H + *E*), 7.44 (m, 2H + *E*), 6.48 (m, 1H+ *E*), 6.09 – 6.02 (m, 1H + *E*), 5.24 (td, *J* = 9.4, 3.0 Hz, 1H + *E*), 5.14 – 5.05 (m, 2H + *E*), 4.90 (dd, *J* = 6.4, 1.3 Hz, 1H + *E*), 4.84 (dt, *J* = 6.4, 1.1 Hz, 1H + *E*), 4.62 (dd, *J* = 19.1, 10.0 Hz, 1H + *E*), 4.26 (m, 1H + *E*), 4.15 (dt, *J* = 12.4, 2.2 Hz, 1H + *E*), 3.77 (m, 1H + *E*), 2.05 (s, 3H + *E*), 2.04 (s, 3H + *E*), 2.03 (s, 3H + *E*), 2.00 (s, 3H + *E*) ppm.

^{13}C{H} NMR (126 MHz, $CDCl_3$) δ 170.1, 170.0, 169.3, 169.1, 169.1, 166.2, 166.1, 133.0, 132.9, 129.9, 129.7, 129.6, 129.6, 128.4, 128.3, 127.6, 127.2, 124.7, 123.6, 83.4, 83.1, 76.2, 76.1, 73.7, 73.7, 69.9, 69.8, 68.1, 68.1, 64.6, 61.9, 61.9, 61.5, 20.6, 20.6, 20.5, 20.5, 20.5, 20.5 ppm

HRMS (ESI): *m/z* calculated for $C_{24}H_{28}O_{11}SNa$: 547.1250 $[M+Na]^+$; found 547.1251

Anal. Calcd for $C_{24}H_{28}O_{11}S$: C, 54.96; H, 5.38. Found: C, 55.20; H, 5.42.

IR (film, cm^{-1}): 2951, 1755, 1720, 1371, 1270, 1041.

(2R,3R,4S,5R,6S)-2-(acetoxymethyl)-6-(phenethylthio)tetrahydro-2H-pyran-3,4,5-triyl triacetate (17m)

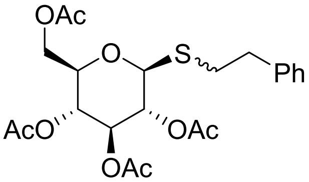

Following the general procedure with a change of stoichiometry: compound **17m** was obtained from styrene (0.1 mmol) and 1-thio-β-D-glucose tetraacetate (0.2 mmol). The crude product was purified by column chromatography using silica gel (hexane/AcOEt) to afford 29 mg of compound **17m** as yellowish oily foam, yield = **63%** for H_2TPP, yield = **74%** (34 mg) for PPIX/p-toluidine, 6 h). Analytical data for compound **17m** are in agreement with the literature data.[16]

^{1}H NMR (500 MHz, $CDCl_3$) δ 7.29 (m, 2H), 7.25 – 7.17 (m, 3H), 5.20 (t, *J* = 9.3 Hz, 1H), 5.05 (dt, *J* = 17.2, 9.7 Hz, 2H), 4.45 (d, *J* = 10.0 Hz, 1H), 4.24 (dd, *J* = 12.4, 5.1 Hz, 1H), 4.14 (dd, *J* = 12.4, 2.5 Hz, 1H), 3.67 (m, 1H), 3.01 – 2.87 (m, 4H), 2.06 (s, 3H), 2.04 (s, 3H), 2.02 (s, 3H), 2.00 (s, 3H) ppm.

^{13}C{H} NMR (126 MHz, $CDCl_3$) δ 170.6, 170.1, 169.3, 169.3, 140.0, 128.5, 128.5, 126.5, 83.45, 75.9, 73.8, 69.8, 68.3, 62.1, 36.2, 31.2, 20.7, 20.6, 20.6, 20.5 ppm.

1-benzyl 4-(3-(((2S,3R,4S,5R,6R)-3,4,5-triacetoxy-6-(acetoxymethyl)tetrahydro-2H-pyran-2-yl)thio)allyl) (tert-butoxycarbonyl)-L-aspartate (17n) *(two diastereoisomers)*

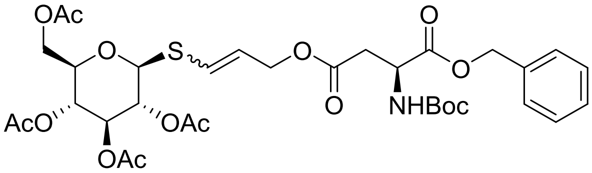

Following the general procedure: compound **17n** was obtained from 1-benzyl 4-(prop-2-yn-1-yl) (*tert*-butoxycarbonyl)aspartate (0.4 mmol) and 1-thio-β-D-glucose tetraacetate (0.2 mmol). The crude product was purified by column chromatography using silica gel (hexane/AcOEt) to afford 71 mg of compound **17n** as a white oily foam **17n** as a mixture of two diastereoisomers (*Z/E* = 50:50), yield = **50%** for H_2TPP, yield = **53%** (75 mg) for H_2TPP/*p*-toluidine, yield = **45%** (64 mg) for PPIX,16 h).

^{1}H NMR (500 MHz, $CDCl_3$) δ 7.35 (q, *J* = 7.0, 6.6 Hz, 5H + *E* (5H)), 6.41 – 6.35 (m, 1H + *E* (1H)), 5.84 (m, 1H + *E* (1H)), 5.47 (m, 1H + *E* (1H)), 5.22 (m, 1H + *E* (1H)), 5.18 – 5.03 (m, 5H + *E* (5H)), 4.72 – 4.51 (m, 4H + *E* (4H)), 4.26 (m, 1H + *E* (1H)), 4.14 (m, 1H + *E* (1H)), 3.74 (m, 1H + *E* (1H)), 3.05 (m, 1H), 2.87 (m, 1H + *E*(1H)), 2.08 (s, 3H), 2.07 (s, 3H), 2.05 (s, 3H), 2.04 (s, 3H), 2.03 (s, 3H), 2.02 (s, 3H), 2.00 (s, 3H), 1.99 (s, 3H), 1.43 (s, 9H + *E* (9H)) ppm.

^{13}C{H} NMR (126 MHz, $CDCl_3$) δ 170.6, 170.6, 170.1, 169.3, 169.1, 169.1, 155.3, 135.4, 128.6, 128.6, 128.4, 128.4, 128.3, 128.3, 126.9, 126.4, 125.5, 123.7, 83.4, 83.0, 80.2, 76.7, 76.3, 76.1, 73.8, 73.7, 69.9, 69.8, 68.1, 68.0, 66.8, 66.8, 65.3, 62.1, 61.9, 61.9, 50.1, 36.8, 28.3, 20.7, 20.6, 20.5, 20.5, 20.5 ppm.

HRMS (ESI): *m/z* calculated for $C_{23}H_{44}O_{15}SN$: 748.2251 $[M+Na]^+$; found 748.2244.

IR (film, cm^{-1}): 3378, 2978, 1754, 1499, 1367, 1227, 1042.

HPLC: >95% purity

[16] Limnios, D.; Kokotos, G. C. *Adv. Synth. Catal.* **2017**, *359*, 323−328.

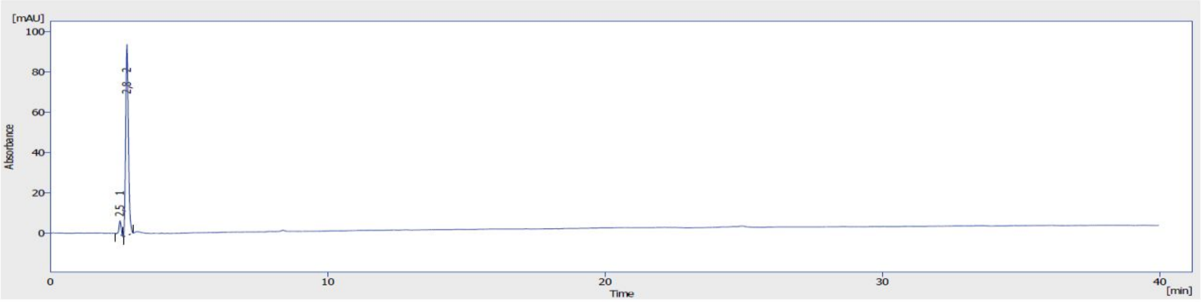

	Reten. Time [min]	Area [mAU.s]	Height [mAU]	Area [%]	Height [%]	W05 [min]	PDA Peak Purity	Compound Name	PDA Best Match Name	PDA Best Match
1	2,517	37,810	7,022	5,5	6,9	0,10	982			
2	2,767	651,252	94,872	94,5	93,1	0,13	660			
	Total	689,062	101,894	100,0	100,0					

4.3. Reductive decarboxylative C(sp^3)–C(sp) bond coupling reaction

Alkynyl *p*-tolylsulfones **19** were prepared as previously reported.[17]

General procedure for the synthesis of *N*-hydroxyphthalimide esters

A carboxylic acid (2.5 mmol, 1 equiv), *N*-hydroxyphthalimide (4.15 mmol, 1.66 equiv), 4-dimethylaminopyridine (DMAP, 0.125 mmol, 0.05 equiv), *N*,*N'*-dicyclohexylcarbodiimide (3.75 mmol, 1.5 equiv), and dry THF (for esters **18A-F**) or dry DCM (for esters **18G-H**) (c=0.2 M) were placed under argon in a round-bottom flask equipped with a stirring bar. The resulting mixture was stirred at room temperature overnight. The mixture was then filtered, and the precipitate was rinsed with cold diethyl ether. The filtrate was concentrated *in vacuo* and the crude product was purified via column chromatography on silica (elution: ethyl acetate/hexane 0-30%).
Analytical data for the previously reported esters **18A**,[18] **18B**,[19] **18C**,[20] **18D**,[21] **18E**,[22] **18F**,[18] **18G**,[23] and **18H**[23] are in agreement with the literature data.

---

[17] Ociepa, M.; Turkowska, J.; Gryko, D. *ACS Catal.* 2018, *8*, 11362−11367

[18] Huihui, K. M. M.; Caputo, J. A.; Melchor, Z.; Olivares, A. M.; Spiewak, A. M.; Johnson, K. A.; DiBenedetto, T. A.; Kim, S.; Ackerman, L. K. G.; Weix, D. J. *J. Am. Chem. Soc.* 2016, *138*, 5016−5019

[19] Zhang, Y. L.; Yang, L.; Wu, J.; Zhu, C.; Wang, P. *Org. Lett.* 2020, *22*, 7768−7772

[20] Dai, J.-J., Teng, X.-X., Fang, W., Xu, J., Xu, H.-J. *Chin. Chem. Lett.* 2022, *33*, 1555–1558

[21] Schwarz, J.; König, B. *Green Chem.* 2016, *18*, 4743-4749

[22] Pratsch, G.; Lackner, G. L.; Overman, L. E. *J. Org. Chem.* 2015, *80*, 6025−6036.

[23] Qin, T.; Malins, L. R.; Edwards, J. T.; Merchant, R. R.; Novak, A. J. E.; Zhong, J. Z.; Mills, R. B.; Yan, M.; Yuan, C.; Eastgate, M. D.; Baran, P. S. *Angew. Chem., Int. Ed.* 2017, *56*, 260−265

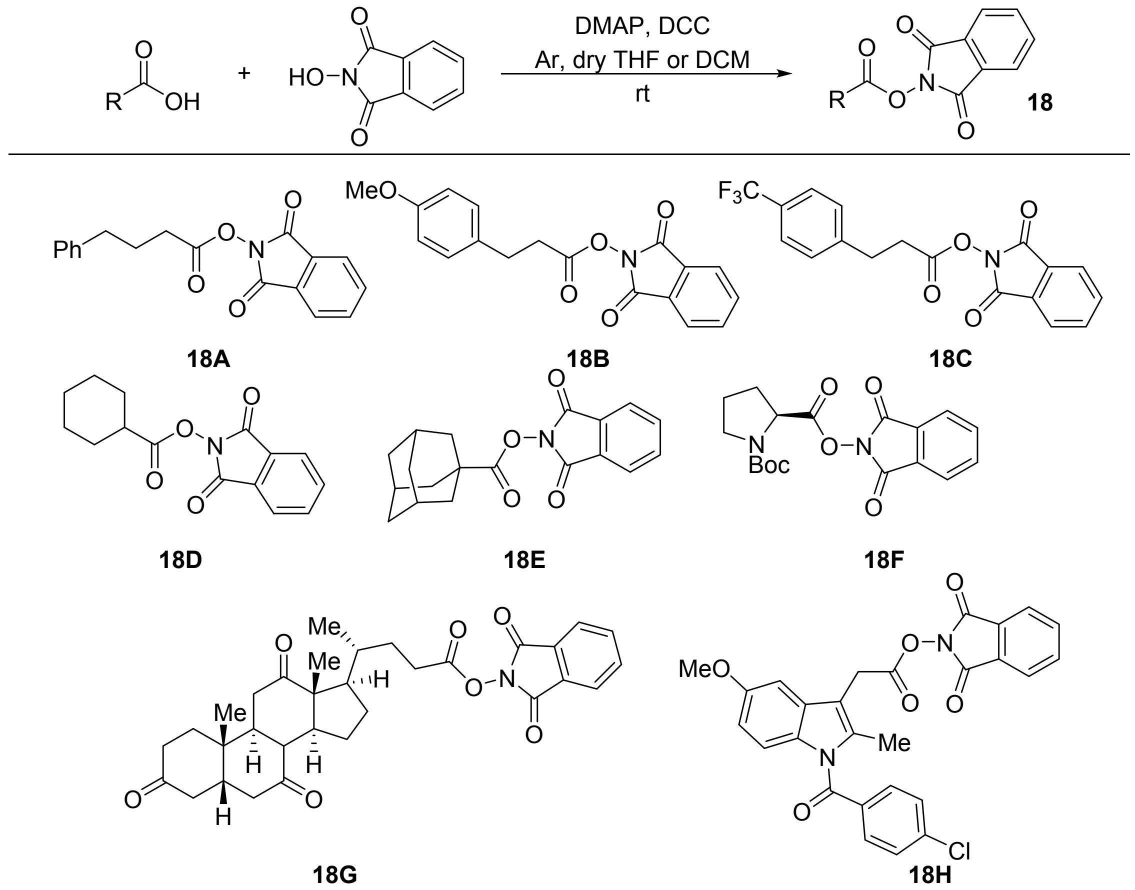

4.3. Reductive decarboxylative C(Sp^3)–C(Sp) bond coupling reaction

Optimization studies:

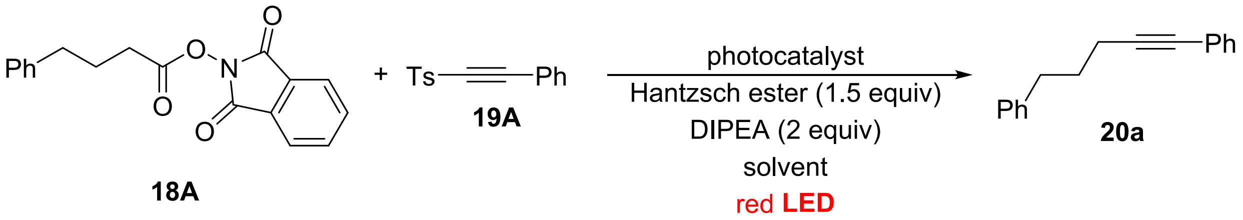

Table S7. Control experiments in acetonitrile.

Entry	Change of Conditions	GC yield [%]
1	–	40
2	no photocatalyst	15
3	no Hantzsch ester	19
4	no DIPEA	3
5	no light	1

Standard reaction conditions: ester (**18A**, 0.1 mmol, 1.0 equiv), alkynyl *p*-tolylsulfone (**19A**, 0.15 mmol, 1.5 equiv), photocatalyst: H_2TPP (2 mol%), Hantzsch ester (0.15 mmol, 1.5 equiv), DIPEA (0.2 mmol, 2 equiv), MeCN (c = 0.1 M), 18 h

Table S8. Control experiments in acetone.

Entry	Change of Conditions	GC yield [%]

1	–	70
2	no light	4
3	no photocatalyst	13
4	no photocatalyst, no Hantzsch ester	0
5	no photocatalyst, 40℃	5

Standard reaction conditions: ester (**18A**, 0.1 mmol, 1.0 equiv.), alkynyl *p*-tolylsulfone (**19A**, 0.15 mmol, 1.5 equiv), photocatalyst: H_2TPP (2 mol%), Hantzsch ester (0.15 mmol, 1.5 equiv), DIPEA (0.2 mmol, 2 equiv), acetone (c = 0.1 M), 18 h

Table S9. Solvent screening.

Entry	Solvent (c = 0.1 M)	GC yield [%]
1	MeCN	40
2	THF	29
3	AcOEt	21
4	DMSO	55
5	DMF	60 (58[a])
6	DMF (suchy)	59
7	DCE	70
8	Aceton	70
9	Aceton/H_2O (4:1 v/v)	66
10	H_2O	25
11	H_2O/DCE (1:1 v/v)	58
12	H_2O/Aceton (1:1 v/v)	47
13	H_2O/DMF (1:1 v/v)	9

Reaction conditions: ester (**18A**, 0.1 mmol, 1.0 equiv), alkynyl *p*-tolylsulfone (**19A**, 0.15 mmol, 1.5 equiv), photocatalyst: H_2TPP (2 mol%), Hantzsch ester (0.15 mmol, 1.5 equiv), DIPEA (0.2 mmol, 2 equiv), solvent (c = 0.1 M), 18 h. [a] Yield of isolated product

Table S10. Photocatalyst screening.

Entry	Porphyrin	GC yield [%]
1	H_2TPP (1 mol%)	66[a]
2	H_2TPP (2 mol%)	70
3	H_2TPP (2 mol%)	60[a]
4	H_2TPP (4 mol%)	57
5	H_2T(4-OMeP)P (2 mol%)	56
6	H_2T(4-CF_3P)P (2 mol%)	11

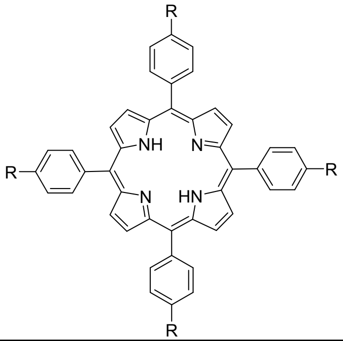

R	
H	H_2TPP
OMe	H_2T(4-OMeP)P
CF_3	H_2T(4-CF_3P)P

Reaction conditions: ester (**18A**, 0.1 mmol, 1.0 equiv), alkynyl *p*-tolylsulfone (**19A**, 0.15 mmol, 1.5 equiv), photocatalyst, Hantzsch ester (0.15 mmol, 1.5 equiv), DIPEA (0.2 mmol, 2 equiv), acetone (c = 0.1 M), 18 h. [a] Reaction performed on 0.2 mmol scale.

Table S11. Additional optimization.

Entry	18A:19A molar ratio	LED power [%]	Time [h]	Photocatalyst loading [mol%]	GC yield [%]
1	1.0:1.5	25	18	2	70
2	1.5:1.0	25	18	2	77
3	1.5:1.0	100	1	2	82[a]
4	1.5:1.0	100	1	1	83[a]
5	1.0:1.0	100	1	2	86 (82[a])
6	1.7:1.0	100	1	2	86 (85[a])
7	2.0:1.0	100	1	1	83 (82[a])

Reaction conditions: ester (**18A**, 0.1-0.2 mmol), alkynyl *p*-tolylsulfone (**19A**, 0.1-0.15 mmol), photocatalyst: H_2TPP, Hantzsch ester (0.15 mmol, 1.5 equiv), DIPEA (0.2 mmol, 2 equiv), acetone (c = 0.1 M). [a] Reaction performed on 0.2 mmol scale.

Table S12. Biocompatible conditions screening.

Entry	Solvent	Photocatalyst loading [mol%]	18A:19A molar ratio	GC yield [%]
1	DMSO	1	1 : 1	65 (54[a])
2	DMF	1	1 : 1	44
3	EtOH	1	1 : 1	58
4	Aceton	1	1 : 1	66
5	MeCN	1	1 : 1	65
6	DMSO	1	2 : 1	61
7	DMSO	2	1 : 1	45
8	DMSO : H_2O (4:1)	1	1 : 1	67
9	DMSO : H_2O (9:1)	1	1 : 1	66
10	DMSO : H_2O (4:1)	1	1 : 1	55
11	DMSO : H_2O (4:1)	1	1 : 1, c = 0.4 M	58
12	DMSO : PBS (4:1)	1	1 : 1	62

Reaction conditions: ester (**18A**, 0.2-0.4 mmol), alkynyl *p*-tolylsulfone (**19A**, 0.2 mmol), photocatalyst: PPIX, Hantzsch ester (0.3 mmol, 1.5 equiv), DIPEA (0.4 mmol, 2 equiv), solvent (c = 0.1 M), 1h. [a] Yield of isolated product.

Scope and limitations studies:

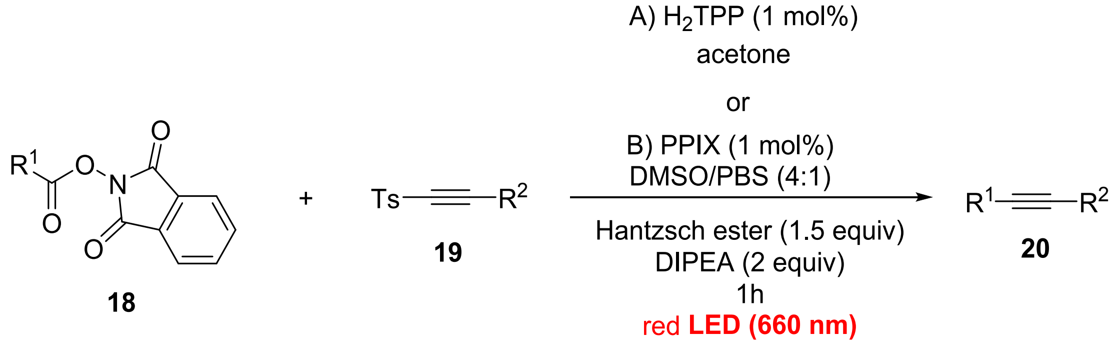

General procedure A: *N*-hydroxyphthalimide ester (0.2 mmol, 1.0 equiv), alkynyl *p*-tolylsulfone (0.2 mmol, 1.0 equiv), Hantzsch ester (76.0 mg, 0.3 mmol, 1.5 equiv) and H_2TPP (1.2 mg, 1 mol%). were placed in a 10 mL vial with a septum equipped with a stirring bar. Next acetone (2 mL) and DIPEA (0.4 mmol, 70 µL, 2.0 equiv) were added. The reaction mixture was stirred under red light irradiation (660 nm, 100% power of the photoreactor) for 1 hour. After that time the mixture was concentrated using rotary evaporator. The crude product was purified by flash chromatography using silica gel (hexane/AcOEt).

General procedure B: *N*-hydroxyphthalimide ester (0.2 mmol, 1.0 equiv), alkynyl *p*-tolylsulfone (0.2 mmol, 1.0 equiv), Hantzsch ester (76.0 mg, 0.3 mmol, 1.5 equiv.) and PPIX (1.1 mg, 1 mol%). were placed in a 10 mL vial with a septum equipped with a stirring bar. Next DMSO (1.6 mL), phosphate-buffered saline solution (0.4 mL) and DIPEA (0.4 mmol, 70 µL, 2.0 equiv) were added. The reaction mixture was stirred under red light irradiation (660 nm, 100% power of the photoreactor) for 1 hour. After that time the mixture was concentrated using rotary evaporator. The crude product was purified by flash chromatography using silica gel (hexane/AcOEt).

pent-1-yne-1,5-diyldibenzene (20a)

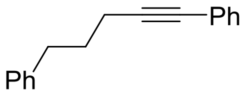

Following the general procedure A, compound **20a** was obtained from 1,3-dioxoisoindolin-2-yl 4-phenylbutanoate (0.2 mmol) and 1-methyl-4-((phenylethynyl)sulfonyl)benzene (0.2 mmol). The crude product was purified by column chromatography using silica gel (hexane/AcOEt) to afford 38 mg (**86%** yield) of product **20a** as a colorless oil. Analytical data for compound **20a** are in agreement with the literature data.[24]

^{1}H NMR (400 MHz, $CDCl_3$) δ 7.44 – 7.37 (m, 2H), 7.32 – 7.24 (m, 5H), 7.28 – 7.13 (m, 3H), 2.78 (t, *J* = 7.6 Hz, 2H), 2.41 (t, *J* = 7.0 Hz, 2H), 1.92 (p, *J* = 7.1 Hz, 2H) ppm.

^{13}C{H} NMR (101 MHz, $CDCl_3$) δ 141.6, 131.5, 128.5, 128.3, 128.2, 127.5, 125.9, 124.0, 89.8, 81.2, 34.8, 30.3, 18.8 ppm.

1-methoxy-4-(4-phenylbut-3-yn-1-yl)benzene (20b)

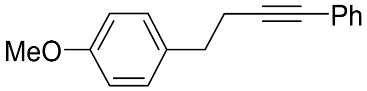

Following the general procedure A, compound **20b** was obtained from 1,3-dioxoisoindolin-2-yl 3-(4-methoxyphenyl)propanoate (0.2 mmol) and 1-methyl-4-((phenylethynyl)sulfonyl)benzene (0.2 mmol).

[24] Ohmiya, H.; Yang, M. Y.; Yamauchi, Y.; Ohtsuka, Y.; Sawamura, M. *Org. Lett.* **2010**, *12*, 1796– 1799

The crude product was purified by column chromatography to afford 29 mg (**61%** yield) of compound **20b** as an oil. Following the general procedure B provided 19 mg (**40%** yield) of the product. Analytical data for compound **20b** are in agreement with the literature data.[25]
^{1}H NMR (500 MHz, $CDCl_3$) δ 7.40 – 7.33 (m, 2H), 7.31 – 7.22 (m, 3H), 7.19 (d, J = 8.7 Hz, 2H), 6.85 (d, J = 8.7 Hz, 2H), 3.79 (s, 3H), 2.86 (t, J = 7.5 Hz, 2H), 2.65 (t, J = 7.5 Hz, 2H) ppm.
^{13}C{H} NMR (126 MHz, $CDCl_3$) δ 158.1, 132.9, 131.5, 129.5, 128.2, 127.6, 123.9, 113.8, 89.6, 81.3, 55.3, 34.3, 22.0 ppm.

1-(4-phenylbut-3-yn-1-yl)-4-(trifluoromethyl)benzene (20c)

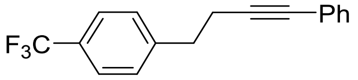

Following the general procedure A, compound **20c** was obtained from 1,3-dioxoisoindolin-2-yl 3-(4-(trifluoromethyl)phenyl)propanoate 1 (0.2 mmol) and 1-methyl-4-((phenylethynyl)sulfonyl)benzene (0.2 mmol). The crude product was purified by column chromatography using silica gel (hexane/AcOEt) to afford 32 mg (**57%** yield) of the product as an off-white solid. Following the general procedure B provided 23 mg (**42%** yield) of the product.
^{1}H NMR (500 MHz, $CDCl_3$) δ 7.57 (d, J = 7.9 Hz, 2H), 7.39 (d, J = 7.9 Hz, 2H), 7.37 – 7.31 (m, 2H), 7.29 – 7.25 (m, 3H), 2.97 (t, J = 7.3 Hz, 2H), 2.72 (t, J = 7.3 Hz, 2H) ppm.
^{13}C{H} NMR (126 MHz, $CDCl_3$) δ 144.6, 131.5, 128.9, 128. 7 (q, J_{C-F} = 32.4 Hz), 128.2, 127.8, 125.3 (q, J_{C-F} = 3.8 Hz), 124.3 (q, J_{C-F} = 271.7 Hz), 123.6, 88.6, 81.8, 34.8, 21.3 ppm.
^{19}F NMR (376 MHz, $CDCl_3$) δ –62.4 ppm.
IR (film, cm^{-1}): 2941, 2925, 1331, 1160, 1111, 1069, 759, 692.
HRMS (EI): m/z calcd for $[C_{17}H_{13}F_3]^+$: 274.0969; found: 274.0966.
Anal. Calcd for $C_{17}H_{13}F_3$: C, 74.44; H, 4.78. Found: C, 74.24; H, 4.87.

(cyclohexylethynyl)benzene (20d)

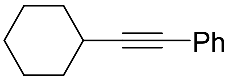

Following the general procedure A, compound **20d** was obtained from 1,3-dioxoisoindolin-2-yl cyclohexanecarboxylate (0.2 mmol) and 1-methyl-4-((phenylethynyl)sulfonyl)benzene (0.2 mmol). The crude product was purified by column chromatography using silica gel (hexane/AcOEt) to afford 25 mg (**68%** yield) of compound **20d** as a colorless oil.
Analytical data for compound **20d** are in agreement with the literature data.[29]
^{1}H NMR (400 MHz, $CDCl_3$) δ 7.42 – 7.35 (m, 2H), 7.31 – 7.20 (m, 3H), 2.64 – 2.53 (m, 1H), 1.93 – 1.83 (m, 2H), 1.82 – 1.70 (m, 2H), 1.60 – 1.47 (m, 3H), 1.43 – 1.28 (m, 3H) ppm.
^{13}C{H} NMR (101 MHz, $CDCl_3$) δ 131.6, 128.1, 127.4, 124.2, 94.4, 80.5, 32.7, 29.7, 25.9, 24.9 ppm.

3-(5-phenylpent-1-yn-1-yl)thiophene (20e)

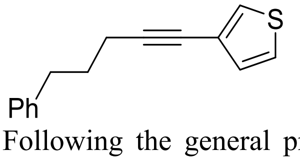

Following the general procedure A, compound **20e** was obtained from 1,3-dioxoisoindolin-2-yl 4-phenylbutanoate (0.2 mmol) and 3-(tosylethynyl)thiophene (0.2 mmol). The crude product was purified by column chromatography using silica gel (hexane/AcOEt) to afford 39 mg (**86%** yield) of compound **20e** as a waxy solid.
Analytical data for compound **20e** are in agreement with the literature data.[26]

---

[25] a) Zhang, X.; Larock, R. C. *J. Am. Chem. Soc.* **2005**, *127*, 12230−12231; b) Ma, S; Wang, L., *J. Org. Chem*, **1998**, *63*, 3497–3498

[26] Lv, L.; Yu, L.; Qiu, Z.; Li, C. *Angew. Chem., Int. Ed.* **2020**, *59*, 6466−6472.

^{1}H NMR (400 MHz, $CDCl_3$) δ 7.35 (dd, *J* = 3.0, 1.1 Hz, 1H), 7.32 – 7.16 (m, 6H), 7.08 (dd, *J* = 5.0, 1.2 Hz, 1H), 2.78 (t, *J* = 7.6 Hz, 2H), 2.40 (t, *J* = 7.0 Hz, 2H), 1.98 – 1.86 (m, 2H) ppm.
^{13}C{H} NMR (101 MHz, $CDCl_3$) δ 141.6, 130.0, 128.5, 128.4, 127.6, 125.9, 125.0, 122.9, 89.3, 76.1, 34.9, 30.3, 18.8 ppm.

1-(5-phenylpent-1-yn-1-yl)naphthalene (20f)

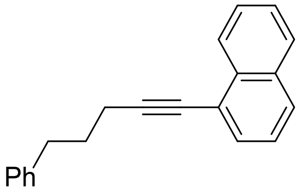

Following the general procedure A, compound **20f** was obtained from 1,3-dioxoisoindolin-2-yl 4-phenylbutanoate (0.2 mmol) and 1-(tosylethynyl)naphthalene (0.2 mmol). The crude product was purified by column chromatography using silica gel (hexane/AcOEt) to afford 50 mg (**93%** yield) of compound **20f** as a colorless oil.
^{1}H NMR (500 MHz, $CDCl_3$) δ 8.36 (d, *J* = 8.3 Hz, 1H), 7.83 (d, *J* = 8.2 Hz, 1H), 7.78 (d, *J* = 8.2 Hz, 1H), 7.63 (dd, *J* = 7.1, 1.2 Hz, 1H), 7.55 (ddd, *J* = 8.3, 6.8, 1.4 Hz, 1H), 7.49 (ddd, *J* = 8.1, 6.8, 1.3 Hz, 1H), 7.40 (dd, *J* = 8.3, 7.1 Hz, 1H), 7.34 – 7.28 (m, 2H), 7.27 – 7.24 (m, 2H), 7.24 – 7.18 (m, 1H), 2.88 (t, *J* = 7.6 Hz, 2H), 2.58 (t, *J* = 7.0 Hz, 2H), 2.03 (p, *J* = 7.1 Hz, 2H) ppm.
^{13}C{H} NMR (126 MHz, $CDCl_3$) δ 141.6, 133.5, 133.2, 130.0, 128.6, 128.4, 128.2, 128.0, 126.5, 126.3, 126.2, 125.9, 125.2, 121.7, 94.9, 79.2, 35.0, 30.5, 19.2 ppm.
IR (film, cm^{-1}): 2937, 799, 773, 699.
HRMS (EI): *m/z* calcd for $[C_{21}H_{18}]^+$: 270.1409; found: 270.1405.
GC: 98% purity

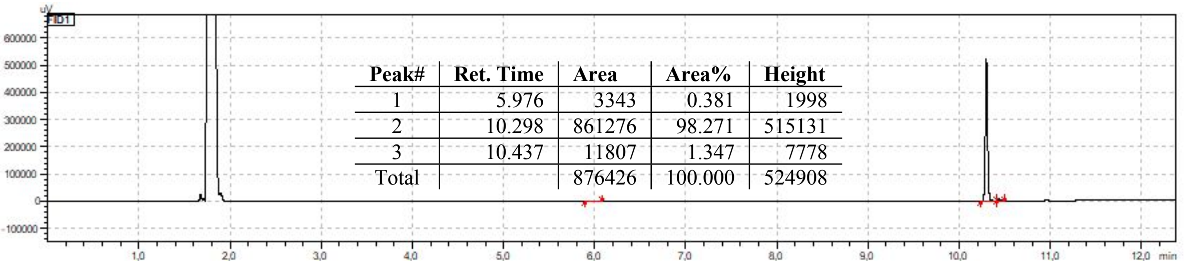

Peak#	Ret. Time	Area	Area%	Height
1	5.976	3343	0.381	1998
2	10.298	861276	98.271	515131
3	10.437	11807	1.347	7778
Total		876426	100.000	524908

triisopropyl(5-phenylpent-1-yn-1-yl)silane (20g)

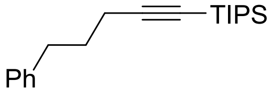

Following the general procedure A, compound **20g** was obtained from 1,3-dioxoisoindolin-2-yl 4-phenylbutanoate (0.2 mmol) and triisopropyl(tosylethynyl)silane (0.2 mmol). The crude product was purified by column chromatography using silica gel (hexane/AcOEt) to afford 27 mg (**44%** yield) of compound **20g** as a colorless oil.
Analytical data for compound **20g** are in agreement with the literature data.[27]
^{1}H NMR (400 MHz, $CDCl_3$) δ 7.32 – 7.23 (m, 2H), 7.22 – 7.15 (m, 3H), 2.76 (t, *J* = 7.6 Hz, 2H), 2.27 (t, *J* = 6.9 Hz, 2H), 1.84 (p, *J* = 6.9 Hz, 2H), 1.13 – 1.03 (m, 21H) ppm.
^{13}C{H} NMR (101 MHz, $CDCl_3$) δ 141.8, 128.6, 128.3, 125.9, 108.7, 80.8, 34.7, 30.7, 19.3, 18.7, 11.3 ppm.

tert-butyldimethyl(3-(4-(4-(trifluoromethyl)phenyl)but-1-yn-1-yl)phenoxy)silane (20h)

[27] Huang, L.; Olivares, A. M.; Weix, D. J. *Angew. Chem., Int. Ed.* **2017**, *56*, 11901−11905

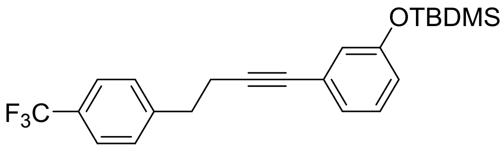

Following the general procedure A, compound **20h** was obtained from 1,3-dioxoisoindolin-2-yl 3-(4-(trifluoromethyl)phenyl)propanoate (0.18 mmol) and *tert*-butyldimethyl(3-(tosylethynyl)phenoxy)silane (0.2 mmol). The crude product was purified by column chromatography using silica gel (hexane/AcOEt) to afford 40 mg (**53%** yield) of compound **20h** as a colorless oil.

^{1}H NMR (500 MHz, $CDCl_3$) δ 7.57 (d, J = 7.9 Hz, 2H), 7.39 (d, J = 7.9 Hz, 2H), 7.13 (t, J = 7.9 Hz, 1H), 6.97 – 6.92 (m, 1H), 6.85 – 6.80 (m, 1H), 6.77 (ddd, J = 8.2, 2.5, 1.1 Hz, 1H), 2.97 (t, J = 7.3 Hz, 2H), 2.71 (t, J = 7.3 Hz, 2H), 0.98 (s, 9H), 0.19 (s, 6H).

^{13}C{H} NMR (126 MHz, $CDCl_3$) δ 155.4, 144.6, 129.3, 128.9, 128.7 (q, J_{C-F} = 32.2 Hz), 125.3 (q, J_{C-F} = 3.8 Hz), 124.6, 124.5, 124.3 (q, J_{C-F} = 271.7 Hz), 123.1, 120.1, 88.4, 81.7, 34.8, 25.6, 21.3, 18.2, –4.5.

^{19}F NMR (470 MHz, $CDCl_3$) δ –62.4 ppm.

IR (film, cm^{-1}): 2955, 2931, 1326, 1166, 1126, 839.

HRMS (APCI): m/z calcd for $C_{23}H_{28}OF_3Si$ $[M+H]^+$: 405.1862; found: 405.1868.

Anal. Calcd for $C_{23}H_{27}F_3OSi$: C, 68.29; H, 6.73. Found: C, 68.40; H, 6.76.

(3r,5r,7r)-1-(phenylethynyl)adamantane (20i)

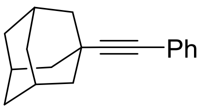

Following the general procedure A, compound **20i** was obtained from 1,3-dioxoisoindolin-2-yl bicyclo[2.2.2]octane-1-carboxylate (0.18 mmol) and 1-methyl-4-((phenylethynyl)sulfonyl)benzene (0.2 mmol). The crude product was purified by column chromatography using silica gel (hexane/AcOEt) to afford 36 mg (**83%** yield) of compound **20i** as a white solid. Following the general procedure B (using 0.18 mmol of **18E** and 0.2 mmol of **19A**) provided 22 mg (51% yield) of the product.

Analytical data for compound **20i** are in agreement with the literature data.[28]

^{1}H NMR (500 MHz, $CDCl_3$) δ 7.40 – 7.35 (m, 2H), 7.28 – 7.22 (m, 3H), 1.99 (s, 3H), 1.96 (s, 6H), 1.72 (s, 6H) ppm.

^{13}C{H} NMR (101 MHz, $CDCl_3$) δ 131.6, 128.1, 127.3, 124.1, 98.4, 79.4, 42.9, 36.4, 30.1, 28.1 ppm.

tert-butyl (S)-2-(phenylethynyl)pyrrolidine-1-carboxylate (20j)

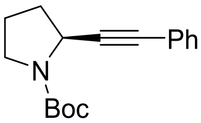

Following the general procedure A, compound **20j** was obtained from 1-(*tert*-butyl) 2-(1,3-dioxoisoindolin-2-yl) (*S*)-pyrrolidine-1,2-dicarboxylate (0.2 mmol) and 1-methyl-4-((phenylethynyl)sulfonyl)benzene (0.2 mmol). The crude product was purified by column chromatography using silica gel (hexane/AcOEt) to afford 40 mg (74% yield) of compound **20j** as a white solid.

Analytical data for compound **20j** are in agreement with the literature data.[29]

^{1}H NMR (400 MHz, $CDCl_3$) δ 7.45 – 7.34 (m, 2H), 7.34 – 7.23 (m, 3H), 4.87 – 4.53 (m, 1H), 3.62 – 3.25 (m, 2H), 2.16 – 1.84 (m, 4H), 1.50 (s, 9H) ppm.

^{13}C{H} NMR (101 MHz, $CDCl_3$) δ 154.2, 131.6, 128.2, 127.9, 123.3, 89.9, 81.6, 79.6, 48.7, 45.7, 33.8, 28.5, 23.8 ppm.

[28] Zhou, Q.-Q.; Guo, W.; Ding, W.; Wu, X.; Chen, X.; Lu, L.-Q.; Xiao, W.-J *Angew. Chem., Int. Ed.* **2015**, *54*, 11196−11199

(5S,9S,10S,13R,14R,17R)-10,13-dimethyl-17-((R)-6-phenylhex-5-yn-2-yl)dodecahydro-3H-cyclopenta[a]phenanthrene-3,7,12(2H,4H)-trione (20k)

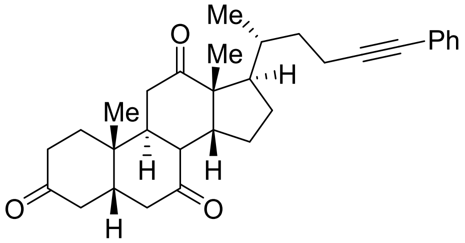

Following the general procedure A, compound **20k** was obtained from 1,3-dioxoisoindolin-2-yl (4*R*)-4-((5*S*,9*S*,10*S*,13*R*,14*S*,17*R*)-10,13-dimethyl-3,7,12-trioxohexadecahydro-1*H*-cyclopenta[a]phenanthren-17-yl)pentanoate (0.2 mmol) and 1-methyl-4-((phenylethynyl)sulfonyl)benzene (0.2 mmol). The crude product was purified by column chromatography using silica gel (hexane/AcOEt) to afford 60 mg (**66%** yield) of compound **20k** as a solid.

Analytical data for compound **20k** are in agreement with the literature data.[29]

^{1}H NMR (400 MHz, $CDCl_3$) δ 7.43 – 7.32 (m, 2H), 7.32 – 7.21 (m, 3H), 2.98 – 2.78 (m, 3H), 2.57 – 1.72 (m, 18H), 1.69 – 1.57 (m, 1H), 1.40 (s, 3H), 1.35 – 1.20 (m, 2H), 1.10 (s, 3H), 0.91 (d, *J* = 6.4 Hz, 3H) ppm.

^{13}C{H} NMR (101 MHz, $CDCl_3$) δ 211.9, 208.9, 208.6, 131.5, 128.2, 127.4, 124.1, 90.5, 80.5, 57.0, 51.8, 49.0, 46.8, 45.7, 45.5, 45.0, 42.8, 38.6, 36.5, 36.0, 35.4, 35.3, 34.5, 27.7, 25.2, 21.9, 18.6, 16.9, 11.9 ppm.

3-(5-phenylpent-1-yn-1-yl)phenyl (4R)-4-((5S,9S,10S,13R,14R,17R)-10,13-dimethyl-3,7,12-trioxohexadecahydro-1H-cyclopenta[a]phenanthren-17-yl)pentanoate (20l)

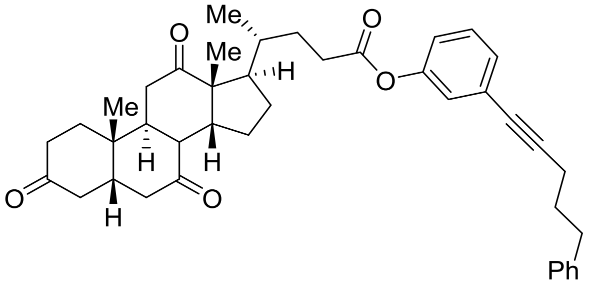

Following the general procedure A, compound **20l** was obtained from 1,3-dioxoisoindolin-2-yl 4-phenylbutanoate (0.2 mmol) and 3-(tosylethynyl)phenyl (4*R*)-4-((5*S*,9*S*,10*S*,13*R*,14*R*,17*R*)-10,13-dimethyl-3,7,12-trioxohexadecahydro-1*H*-cyclopenta[a]phenanthren-17-yl)pentanoate (0.2 mmol). The crude product was purified by column chromatography using silica gel (hexane/AcOEt) to afford 61 mg (**49%** yield) of compound **20l** as a white solid.

^{1}H NMR (500 MHz, $CDCl_3$) δ 7.33 – 7.25 (m, 4H), 7.25 – 7.16 (m, 3H), 7.15 – 7.09 (m, 1H), 7.00 (dt, *J* = 7.6, 2.1 Hz, 1H), 2.95 – 2.81 (m, 3H), 2.78 (t, *J* = 7.6 Hz, 2H), 2.64 (ddd, *J* = 15.9, 9.1, 5.3 Hz, 1H), 2.51 (ddd, *J* = 16.0, 8.7, 7.3 Hz, 1H), 2.41 (t, *J* = 7.0 Hz, 2H), 2.37 – 1.81 (m, 17H), 1.68 – 1.57 (m, 1H), 1.56 – 1.46 (m, 1H), 1.42 – 1.35 (s+m, 3H+2H), 1.09 (s, 3H), 0.92 (d, *J* = 6.6 Hz, 3H) ppm.

^{13}C{H} NMR (126 MHz, $CDCl_3$) δ 211.8, 208.9, 208.6, 172.3, 150.5, 141.5, 129.1, 129.0, 128.5, 128.4, 125.9, 125.3, 124.6, 121.0, 90.8, 80.3, 56.9, 51.8, 49.0, 46.8, 45.7, 45.5, 44.9, 42.8, 38.6, 36.5, 36.0, 35.5, 35.3, 34.8, 31.5, 30.4, 30.2, 27.6, 25.1, 21.9, 18.8, 18.6, 11.8 ppm.

IR (film, cm^{-1}): 2928, 2874, 1759, 1712, 1168, 1153, 1123.

HRMS (ESI): *m/z* calcd for $C_{41}H_{48}O_5Na$ $[M+Na]^+$: 643.3399; found: 643.3406.

HPLC: > 95% purity

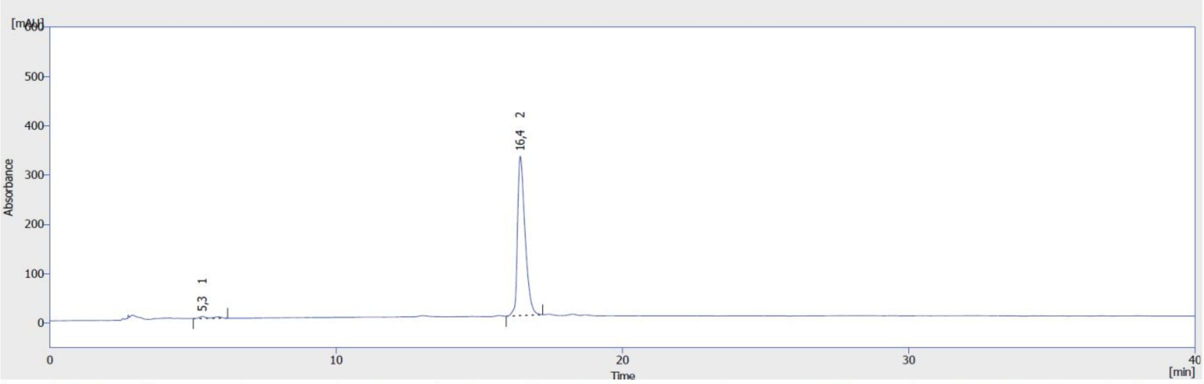

	Reten. Time [min]	Area [mAU.s]	Height [mAU]	Area [%]	Height [%]	W05 [min]	PDA Peak Purity	Compound Name	PDA Best Match Name	PDA Best Match
1	5,333	60,212	2,112	2,0	1,3	0,25	1000			
2	16,433	2966,120	161,751	98,0	98,7	0,30	957			
	Total	3026,332	163,862	100,0	100,0					

(4-chlorophenyl)(5-methoxy-2-methyl-3-(3-phenylprop-2-yn-1-yl)-1H-indol-1-yl)methanone (20m)

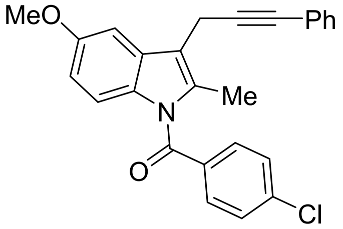

Following the general procedure A, compound **20m** was obtained from 1,3-dioxoisoindolin-2-yl 2-(1-(4-chlorobenzoyl)-5-methoxy-2-methyl-1*H*-indol-3-yl)acetate (0.2 mmol) and 1-methyl-4-((phenylethynyl)sulfonyl)benzene (0.2 mmol). The crude product was purified by column chromatography using silica gel (hexane/AcOEt) to afford 46 mg (55% yield) of compound **20m** as an off-white solid. Following the general procedure B provided 11 mg (13% yield) of the product. Analytical data for compound **20m** are in agreement with the literature data.[29]

^{1}H NMR (400 MHz, $CDCl_3$) δ 7.67 (d, *J* = 8.5 Hz, 2H), 7.46 (d, *J* = 8.5 Hz, 2H), 7.41 – 7.36 (m, 2H), 7.29 – 7.23 (m, 3H), 7.18 (d, *J* = 2.5 Hz, 1H), 6.87 (d, *J* = 9.0 Hz, 1H), 6.68 (dd, *J* = 9.0, 2.6 Hz, 1H), 3.83 (s, 3H), 3.79 (s, 2H), 2.43 (s, 3H) ppm.

^{13}C{H} NMR (101 MHz, $CDCl_3$) δ 168.3, 155.9, 139.2, 134.4, 134.0, 131.6, 131.2, 130.9, 130.4, 129.1, 128.2, 127.8, 123.5, 114.9, 114.7, 111.7, 101.5, 86.7, 81.1, 55.7, 15.0, 13.3 ppm.

3-(5-phenylpent-1-yn-1-yl)phenyl 2-(1-(4-chlorobenzoyl)-5-methoxy-2-methyl-1H-indol-3-yl)acetate (20n)

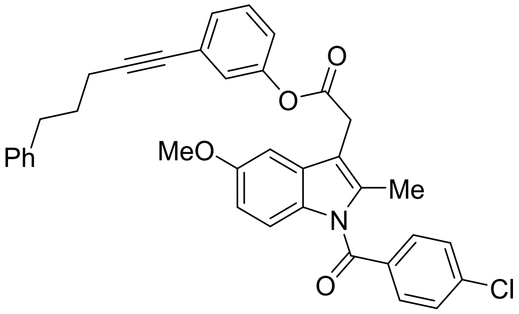

Following the general procedure A, compound **20n** was obtained from 1,3-dioxoisoindolin-2-yl 4-phenylbutanoate (0.2 mmol) and 3-(tosylethynyl)phenyl 2-(1-(4-chlorobenzoyl)-5-methoxy-2-methyl-1*H*-indol-3-yl)acetate (0.2 mmol). The crude product was purified by column chromatography using silica gel (hexane/AcOEt) and subsequent preparative TLC to afford 53 mg (**46%** yield) of compound **20n** as a white solid.

^{1}H NMR (400 MHz, $CDCl_3$) δ 7.67 (d, *J* = 8.5 Hz, 2H), 7.47 (d, *J* = 8.5 Hz, 2H), 7.32 – 7.26 (m, 4H), 7.23 – 7.16 (m, 3H), 7.12 – 7.08 (m, 1H), 7.05 (d, *J* = 2.5 Hz, 1H), 7.02 – 6.96 (m, 1H), 6.90 (d, *J* = 9.0 Hz, 1H), 6.69 (dd, *J* = 9.0, 2.5 Hz, 1H), 3.89 (s, 2H), 3.84 (s, 3H), 2.77 (t, *J* = 7.5 Hz, 2H), 2.45 (s, 3H), 2.40 (t, *J* = 7.0 Hz, 2H), 1.91 (p, *J* = 7.2 Hz, 2H) ppm.

^{13}C{H} NMR (126 MHz, $CDCl_3$) δ 169.1, 168.3, 156.1, 150.4, 141.5, 139.3, 136.2, 133.8, 131.2, 130.9, 130.8, 130.4, 129.2, 129.1, 128.8, 128.5, 128.4, 125.9, 125.4, 124.4, 120.9, 115.0, 111.9, 111.9, 101.2, 91.0, 80.1, 55.7, 34.8, 30.5, 30.1, 18.8, 13.4 ppm.

IR (film, cm^{-1}): 2924, 2852, 1758, 1683, 1477, 1459, 1319, 1152, 1124.

HRMS (ESI): *m/z* calcd for $C_{36}H_{31}NO_4Cl$ $[M+H]^+$: 576.1942; found: 576.1934.

HPLC: >95% purity

[29] Guo, J.; Wang, Y.; Li, Y.; Lu, K.; Liu, S.; Wang, W.; Zhang, Y. *Adv. Synth. Catal.* 2020, *362*, 3898−3904.

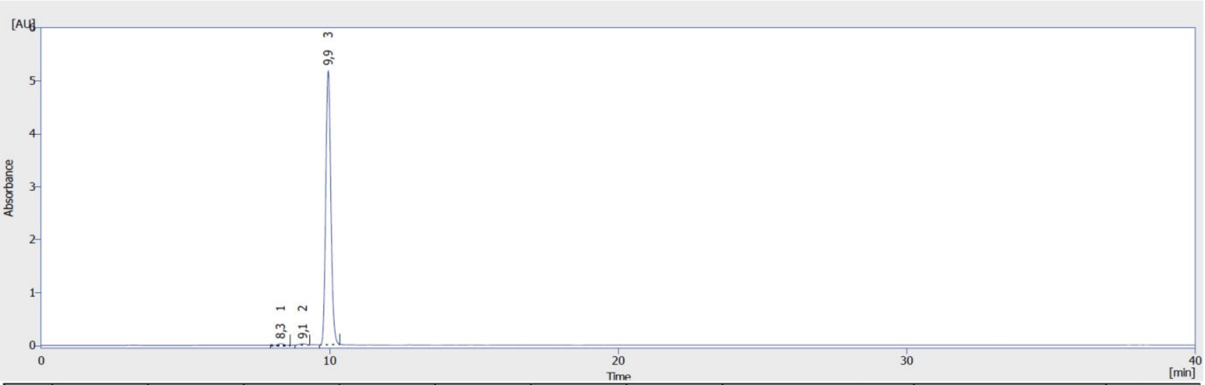

	Reten. Time [min]	Area [mAU.s]	Height [mAU]	Area [%]	Height [%]	W05 [min]	PDA Peak Purity	Compound Name	PDA Best Match Name	PDA Best Match
1	8,300	196,611	15,886	0,6	0,6	0,20	999			
2	9,067	185,480	11,819	0,6	0,5	0,28	998			
3	9,950	31856,420	2589,939	98,8	98,9	0,20	781			
	Total	32238,511	2617,644	100,0	100,0					

5. NMR spectra

5.1. *N*-(4-aminophenyl)-2-propylpentanamide (11)

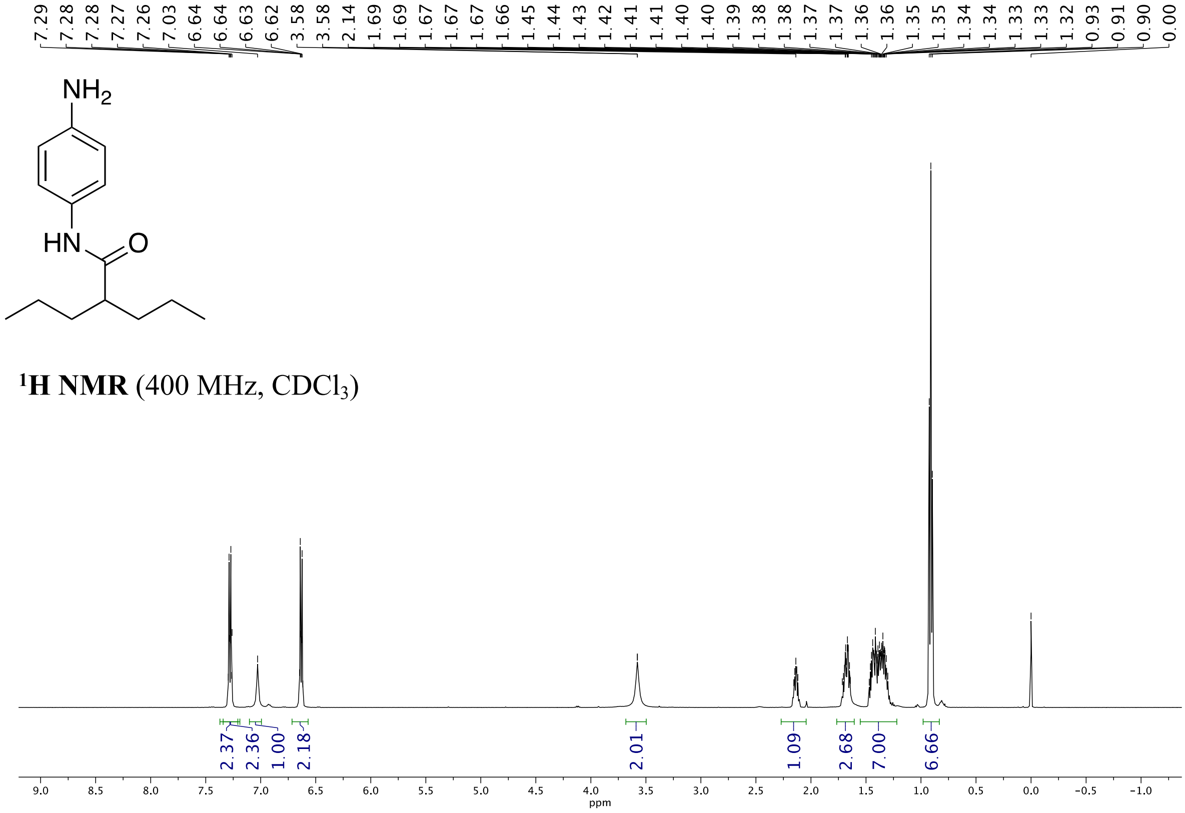

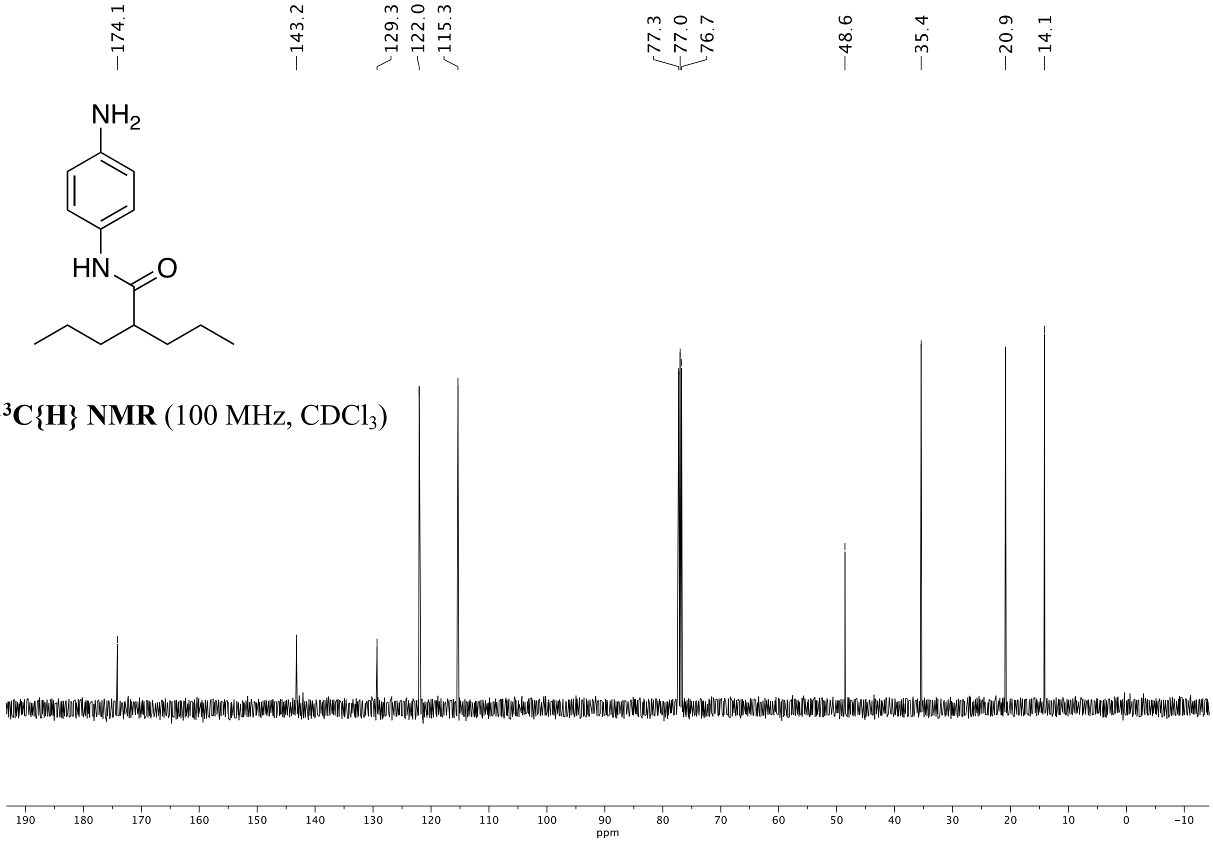

5.2. ((*1s,3s*)-adamantan-1-yl)(styryl)sulfane (17d) (two diastereoisomers)

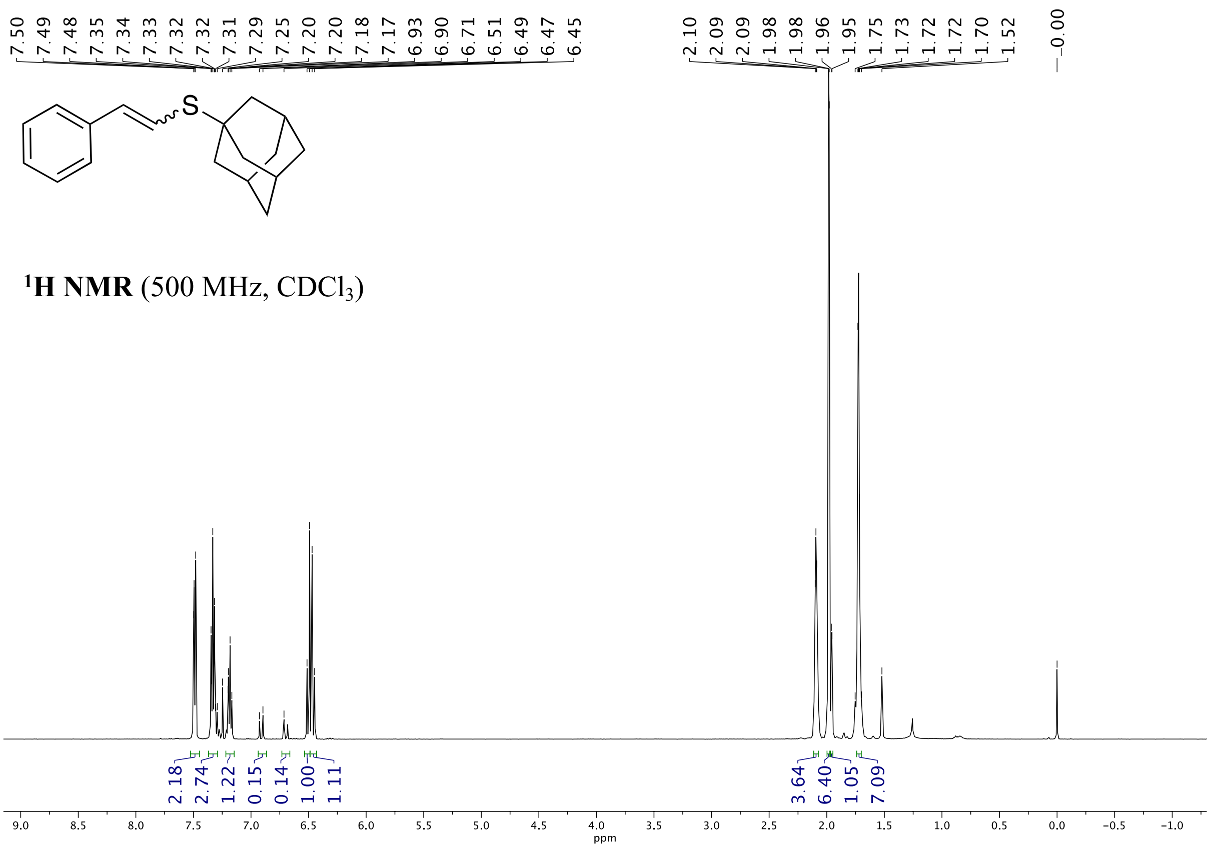

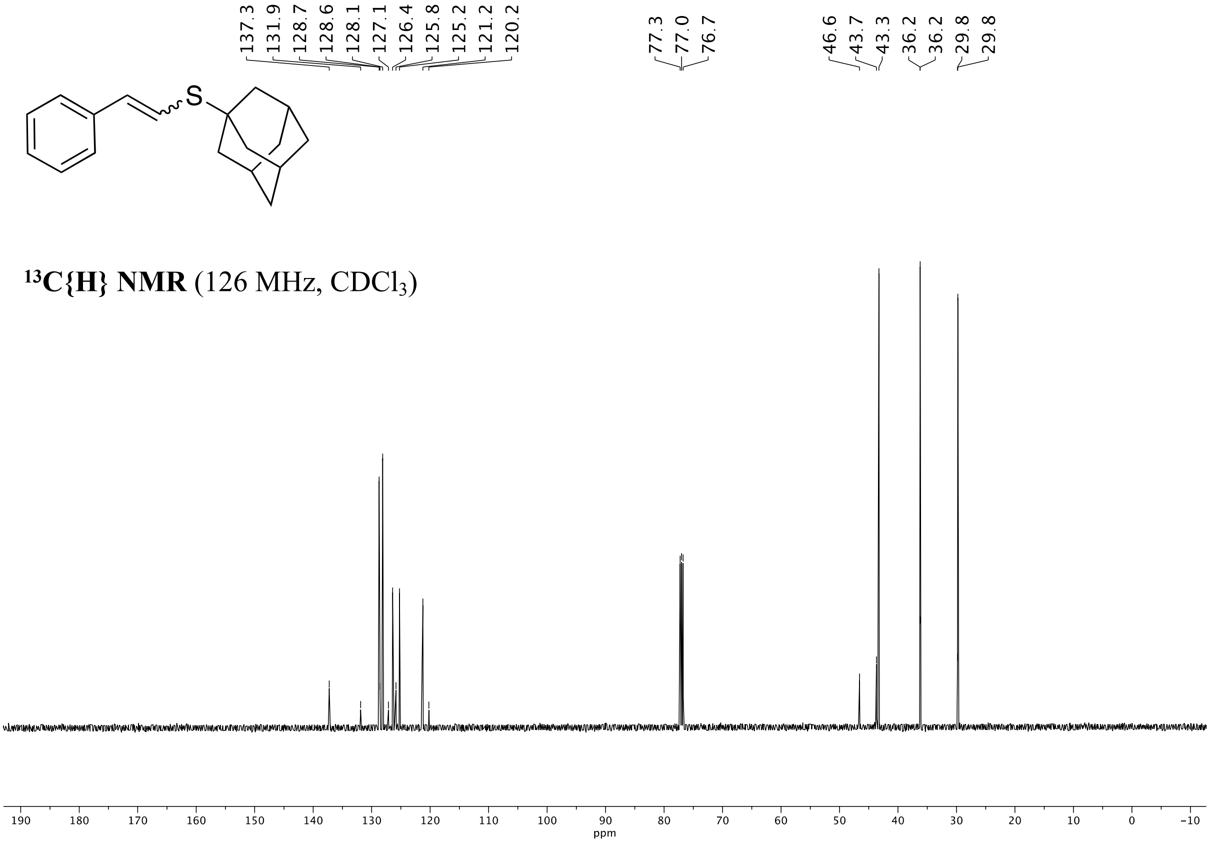

5.3. methyl *N*-(*tert*-butoxycarbonyl)-*S*-styryl-*L*-cysteinyl-*L*-phenylalaninate (17f)

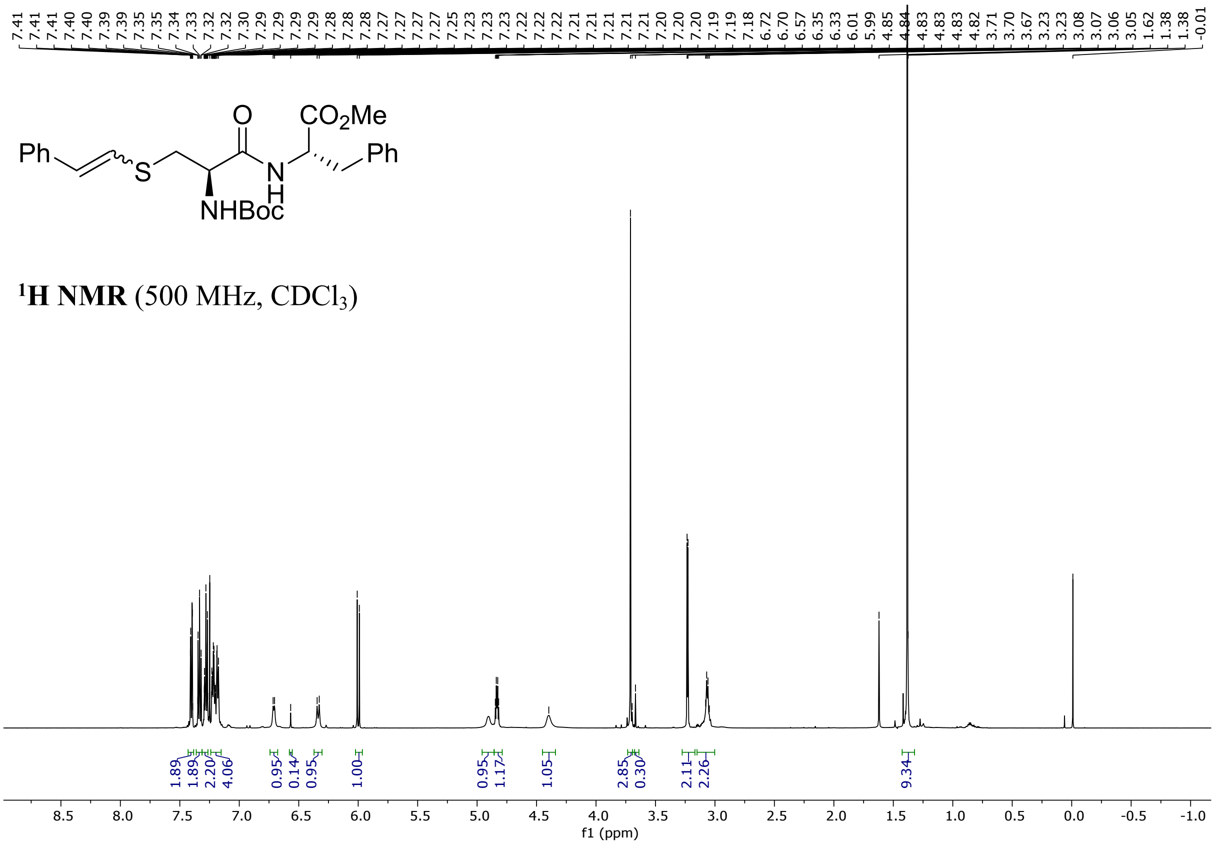

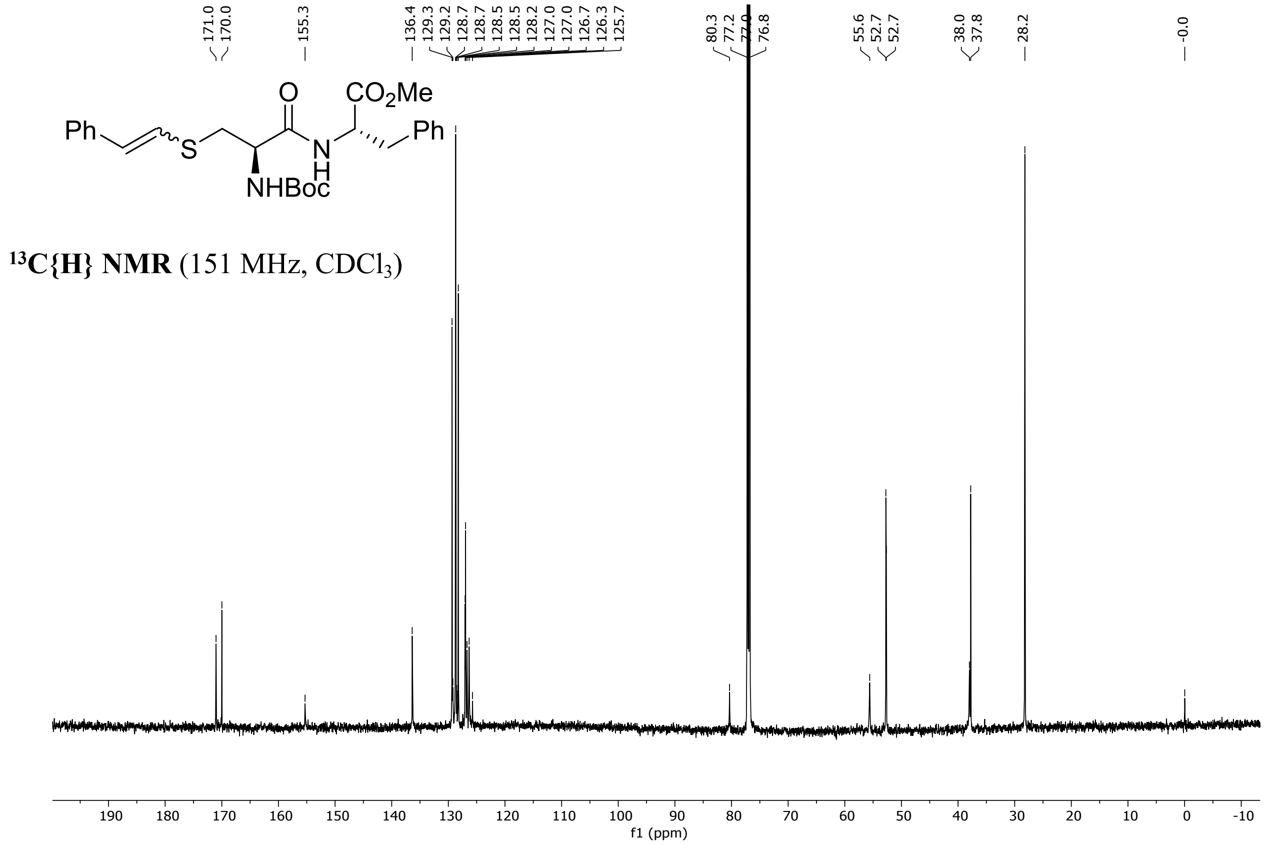

5.4. (2*R*,3*R*,4*S*,5*R*,6*S*)-2-(acetoxymethyl)-6-((4-fluorostyryl)thio)tetrahydro-2*H*-pyran-3,4,5-triyl triacetate (17h) (two diastereoisomers)

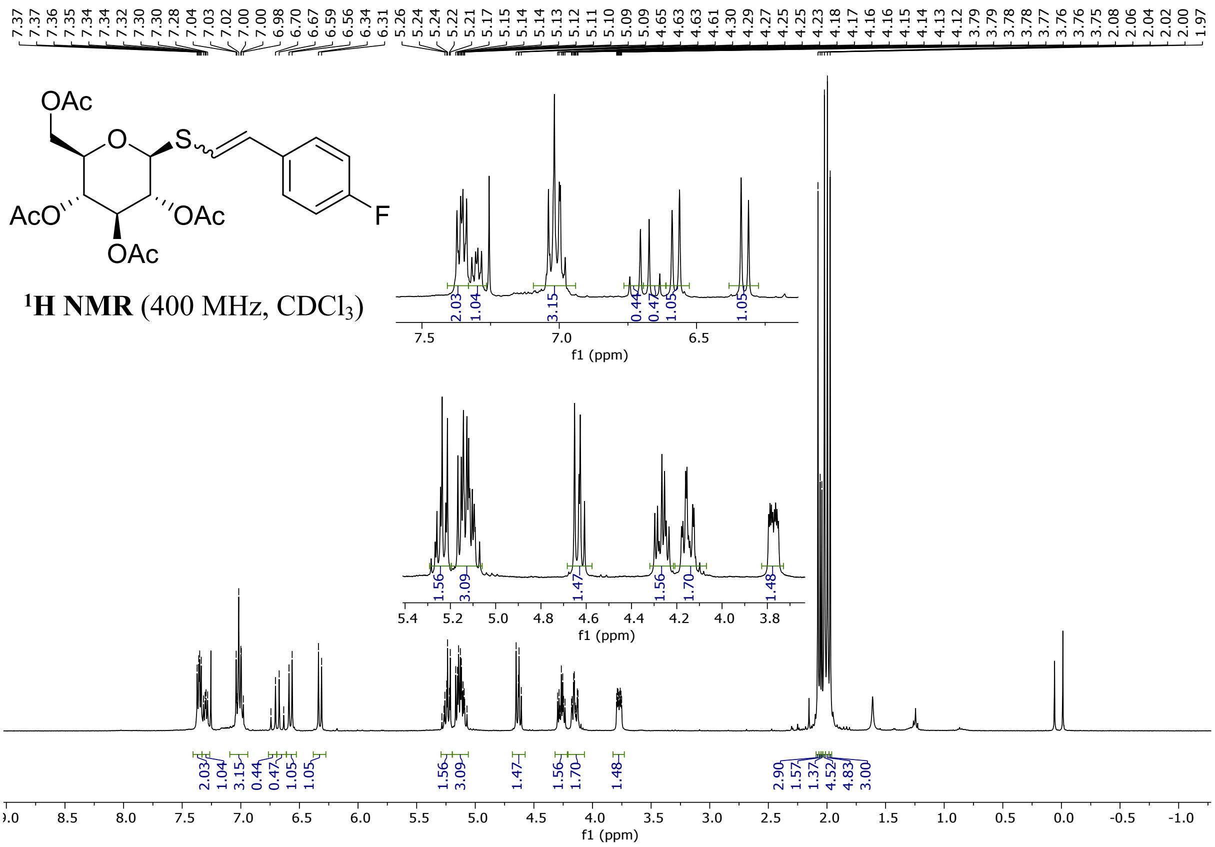

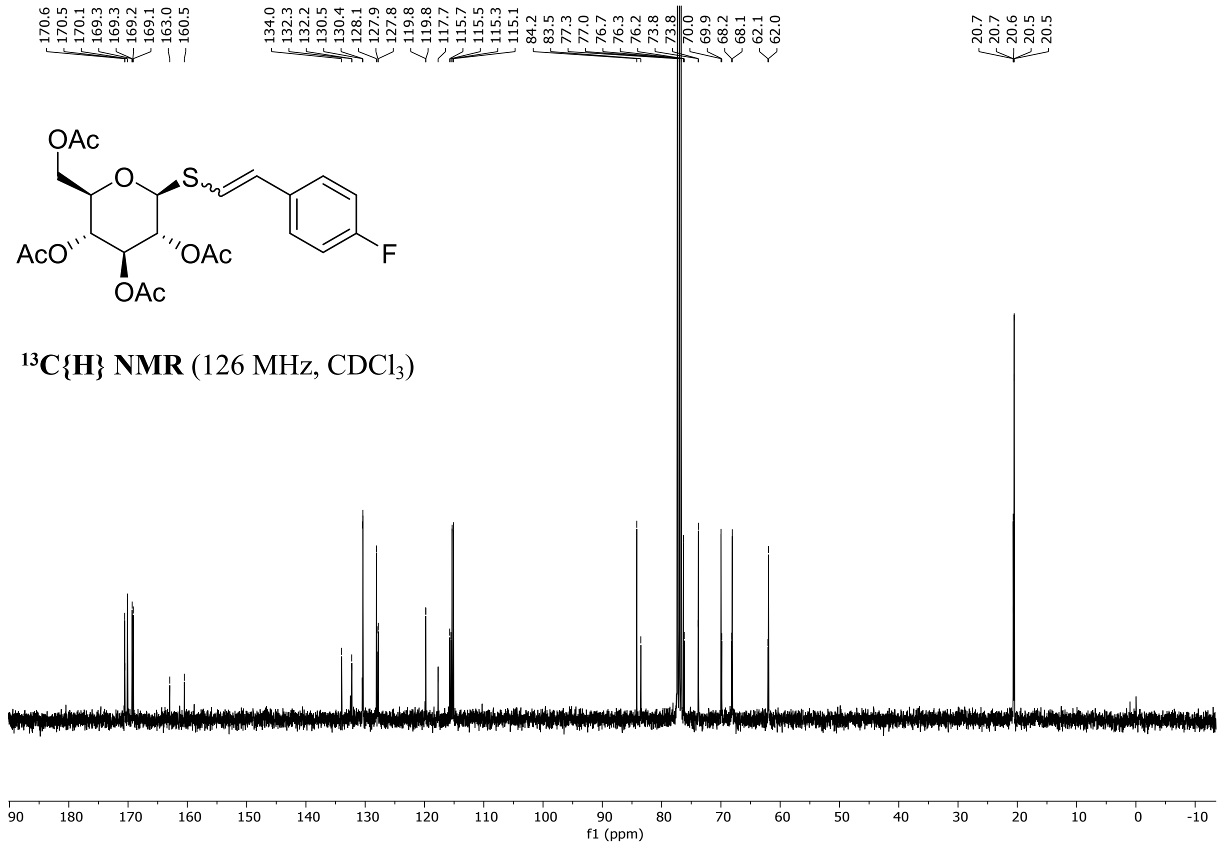
170.6
170.5
170.1
169.3
169.3
169.2
169.1
163.0
160.5
134.0
132.3
132.2
130.5
130.4
128.1
127.9
127.8
119.8
119.8
117.7
115.7
115.5
115.3
115.1
84.2
83.5
77.3
77.0
76.7
76.3
76.2
73.8
73.8
70.0
69.9
68.2
68.1
62.1
62.0
20.7
20.7
20.6
20.5
20.5
OAc
O
S
AcO
OAc
OAc
F
^{13}C{H} NMR (126 MHz, CDCl$_3$)
f1 (ppm)

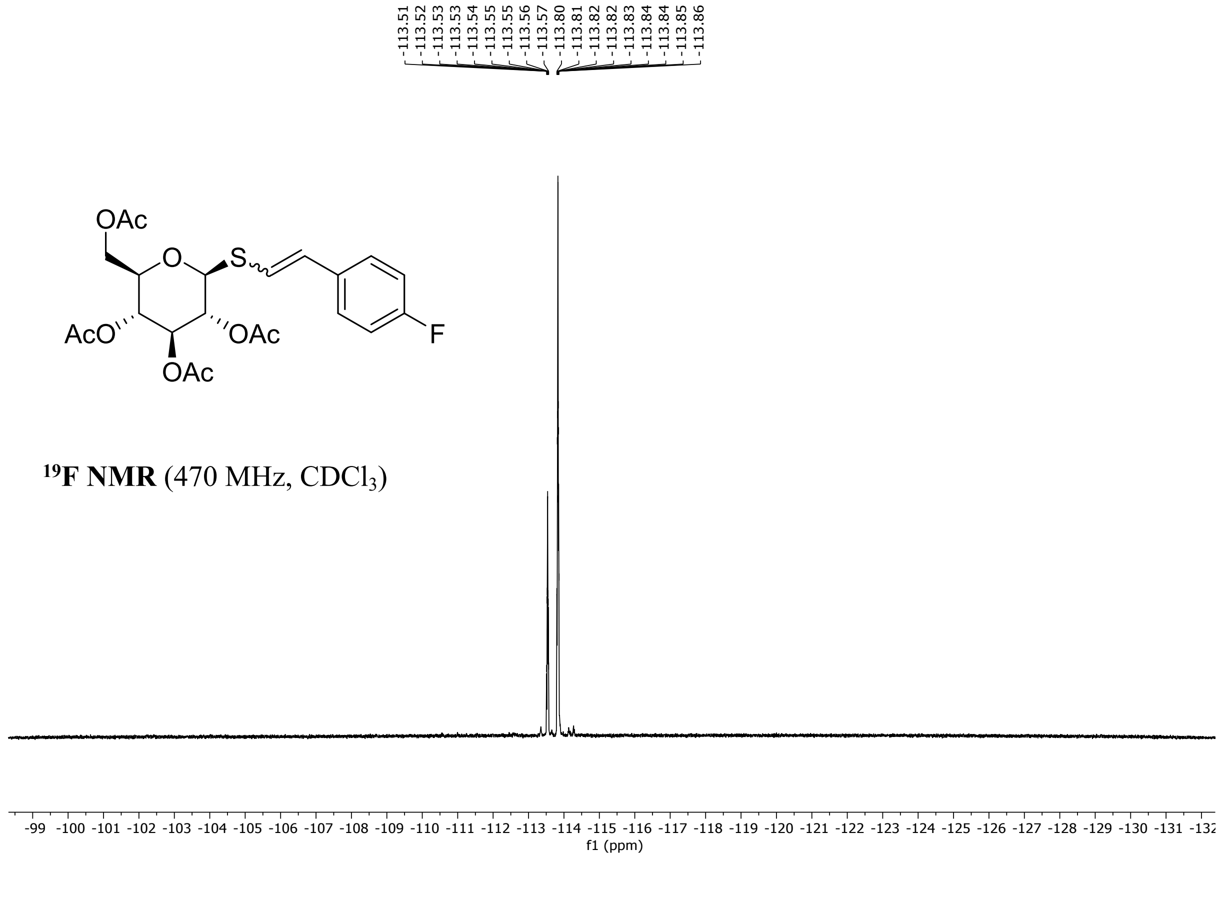
-113.51
-113.52
-113.53
-113.54
-113.55
-113.55
-113.56
-113.57
-113.80
-113.81
-113.81
-113.82
-113.83
-113.84
-113.85
-113.86
OAc
O
S
AcO
OAc
OAc
F
^{19}F NMR (470 MHz, CDCl$_3$)
f1 (ppm)

5.5. (2*R*,3*R*,4*S*,5*R*,6*S*)-2-(acetoxymethyl)-6-((4-methoxystyryl)thio)tetrahydro-2*H*-pyran-3,4,5-triyl triacetate (17i) (two diastereoisomers)

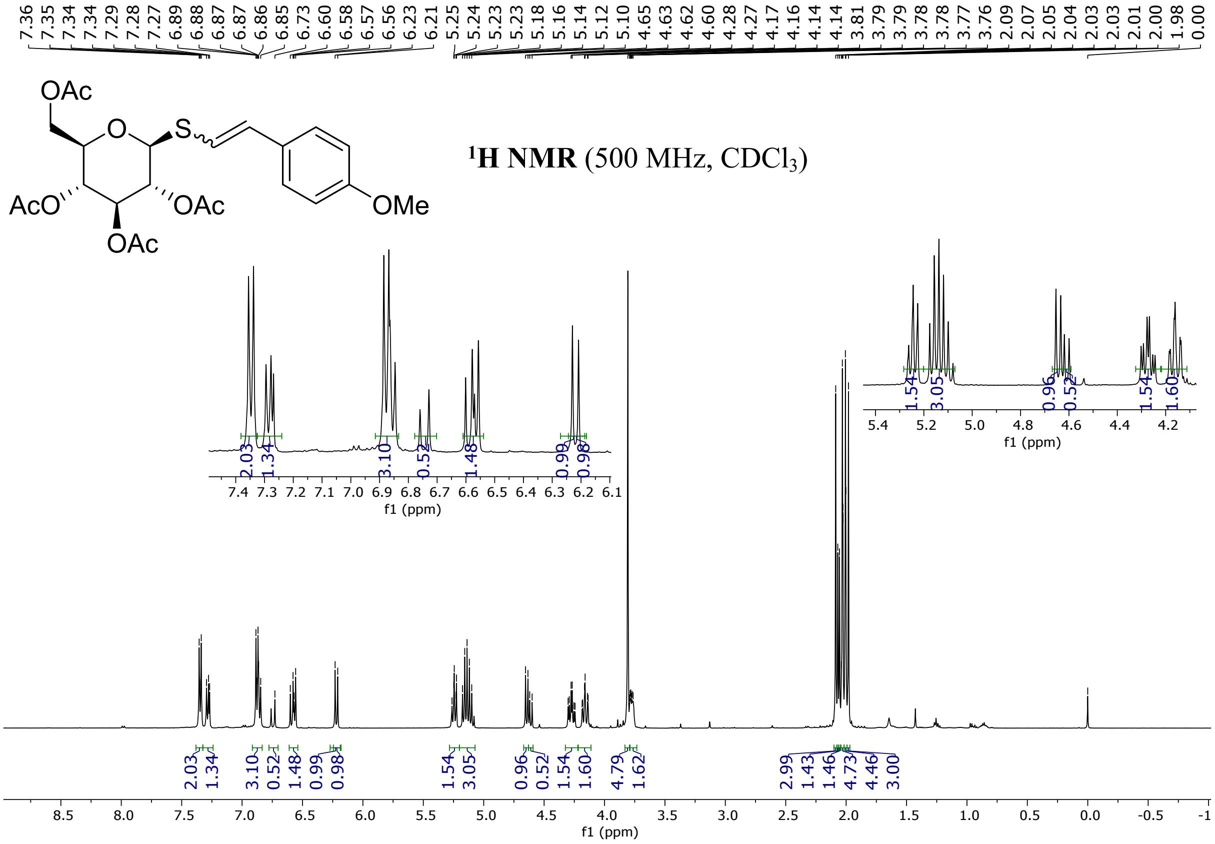

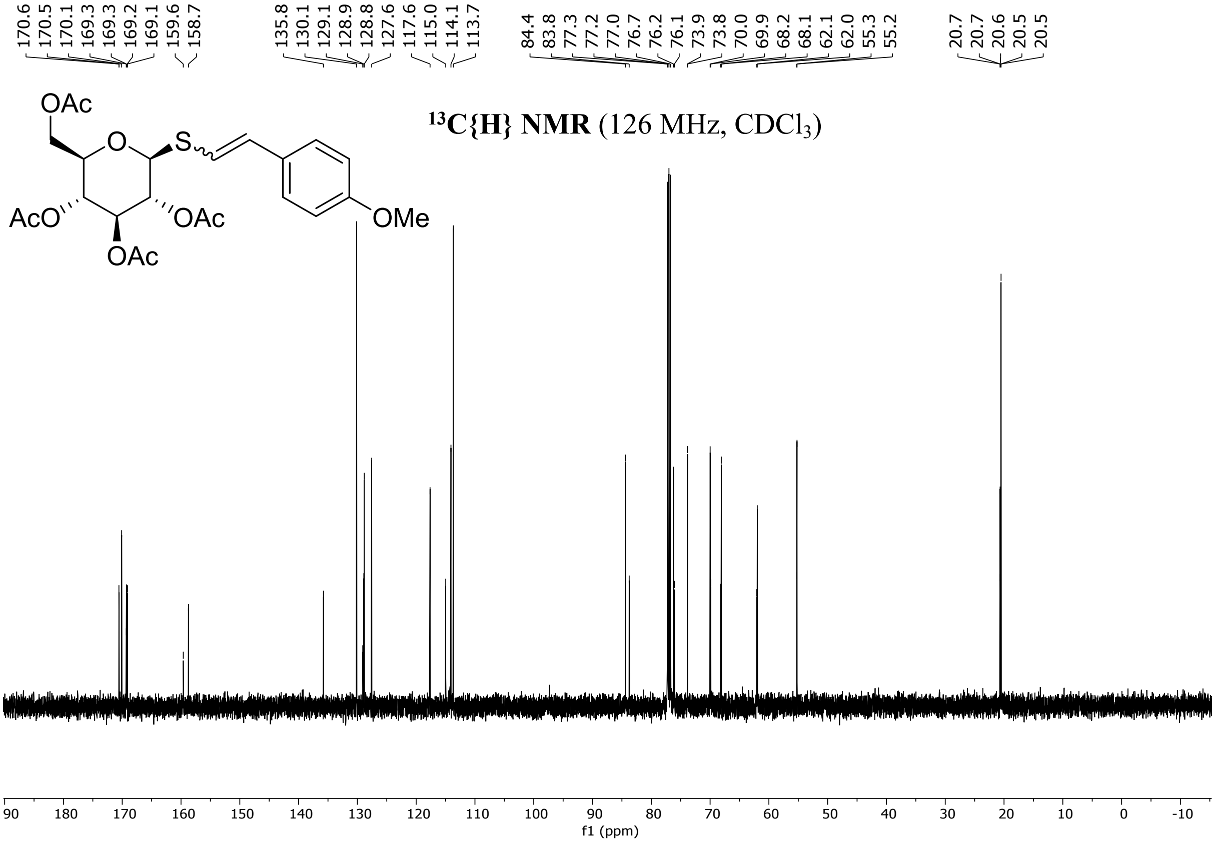

5.6. (2*R*,3*R*,4*S*,5*R*,6*S*)-2-(acetoxymethyl)-6-((1,2-diphenylvinyl)thio)tetrahydro-2*H*-pyran-3,4,5-triyl triacetate (17j) (two diastereoisomers)

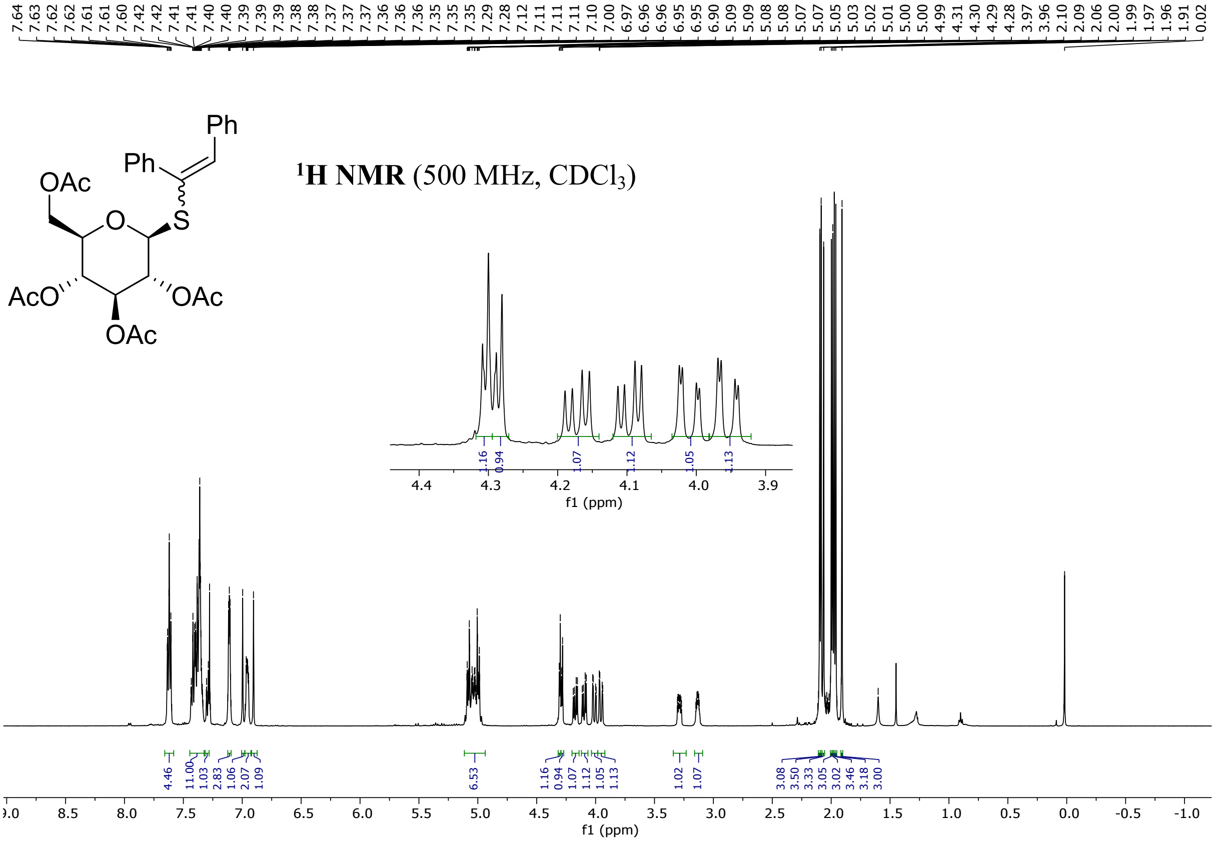

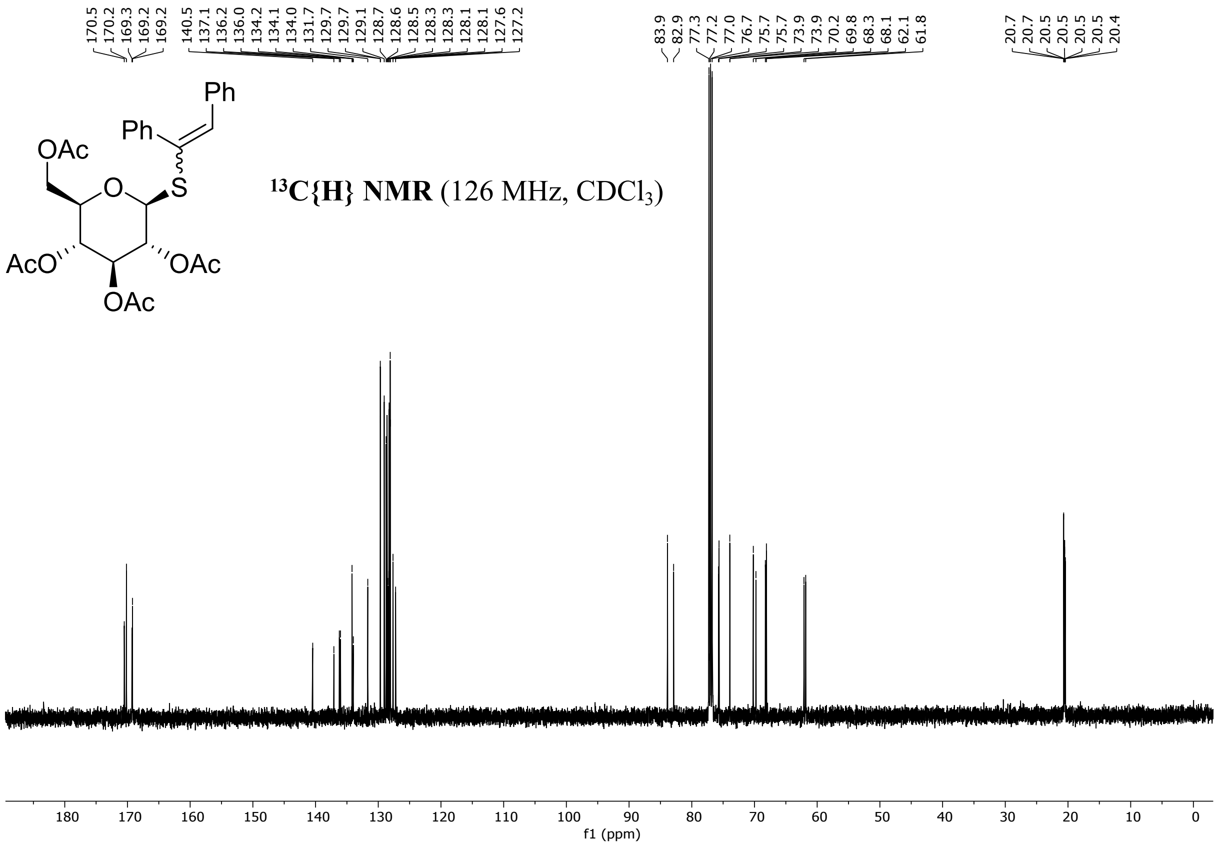

5.7. (2*R*,3*R*,4*S*,5*R*,6*S*)-2-(acetoxymethyl)-6-(oct-1-en-1-ylthio)tetrahydro-2*H*-pyran-3,4,5-triyl triacetate (17k) (two diastereoisomers)

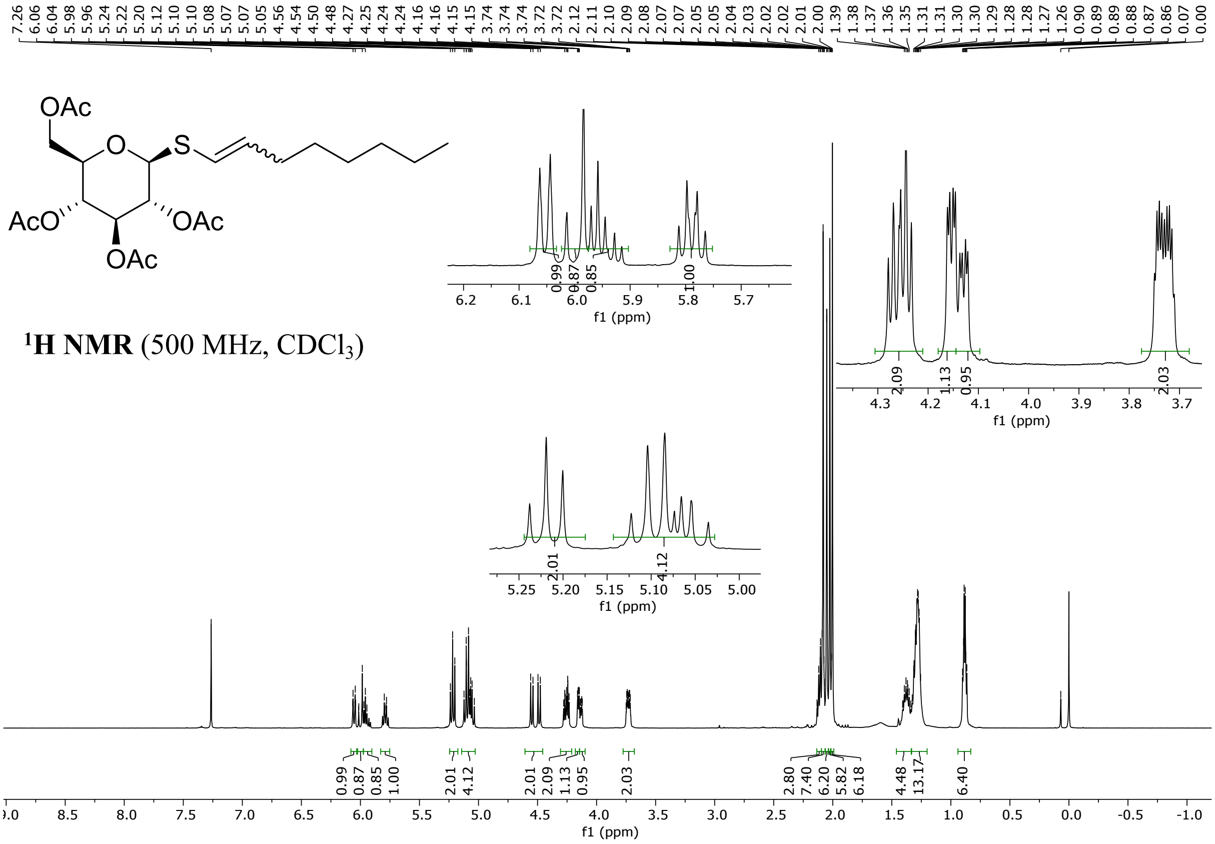

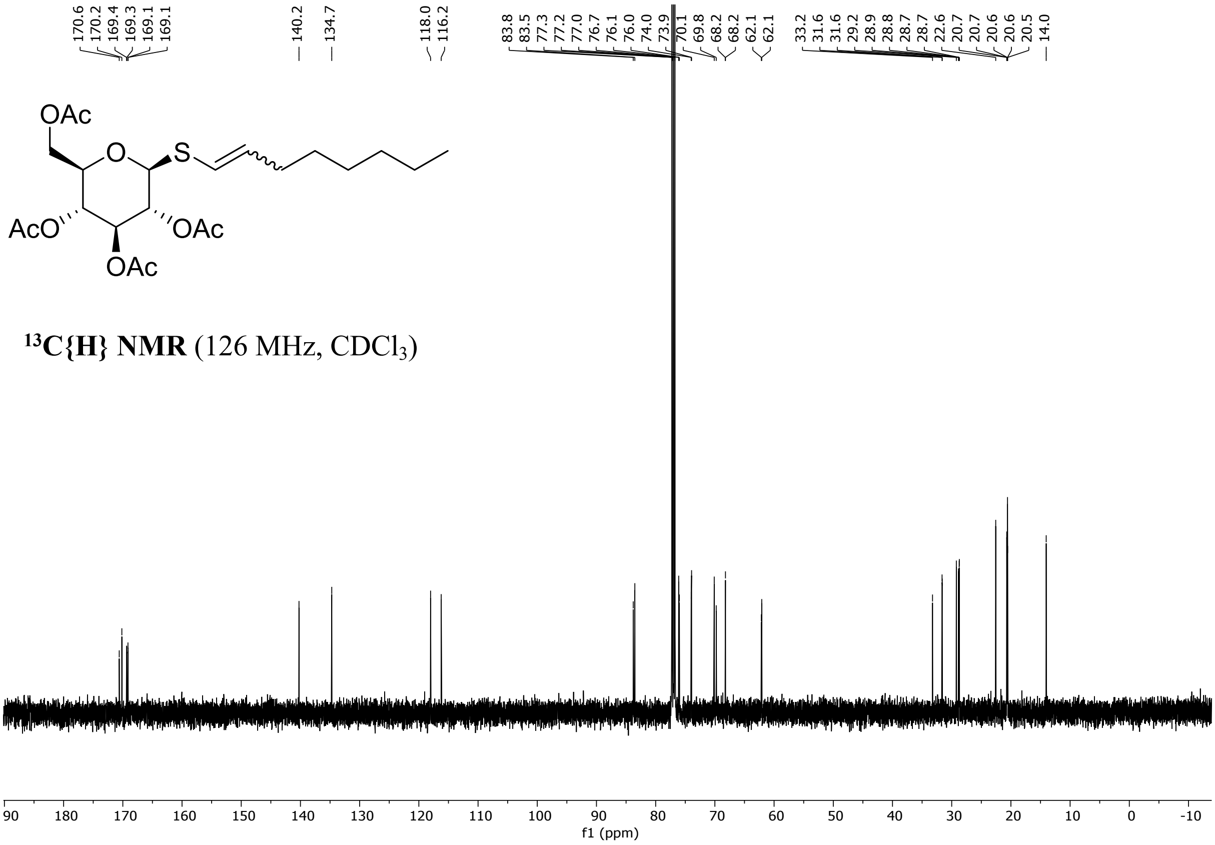

5.8. (2*R*,3*R*,4*S*,5*R*,6*S*)-2-(acetoxymethyl)-6-((3-(benzoyloxy)prop-1-en-1-yl)thio)tetrahydro-2*H*-pyran-3,4,5-triyl triacetate (17l) (two diastereoisomers)

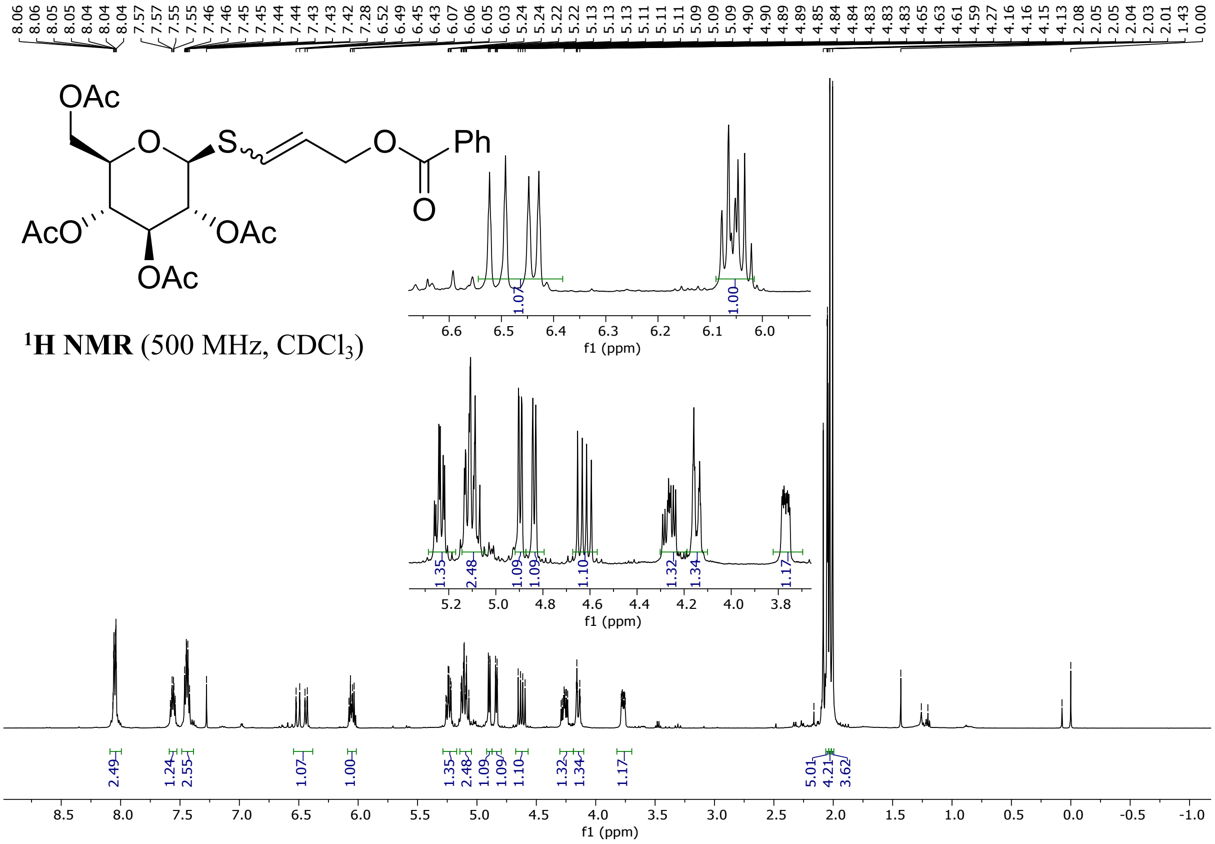

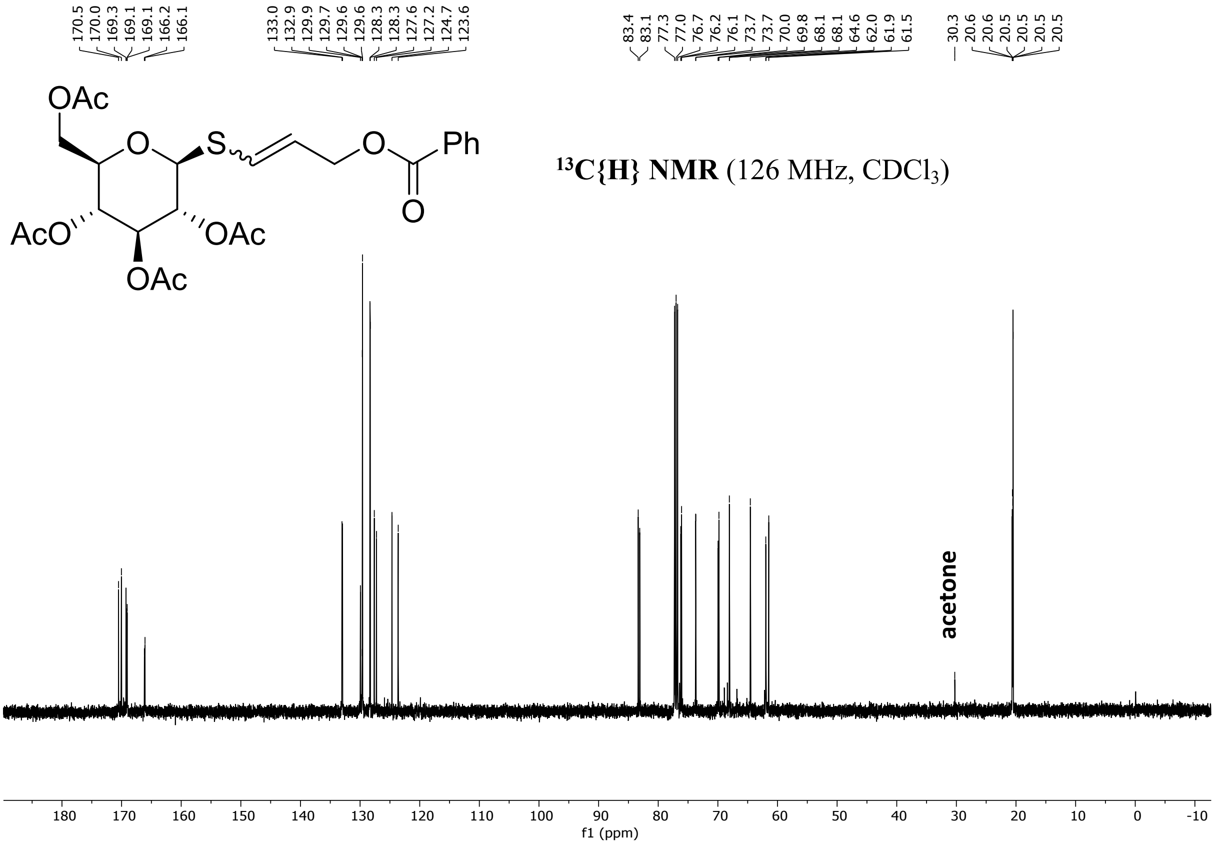

5.9. 1-benzyl 4-(3-(((2*S*,3*R*,4*S*,5*R*,6*R*)-3,4,5-triacetoxy-6-(acetoxymethyl)tetrahydro-2*H*-pyran-2-yl)thio)allyl) (*tert*-butoxycarbonyl)-*L*-aspartate (17n) (two diastereoisomers)

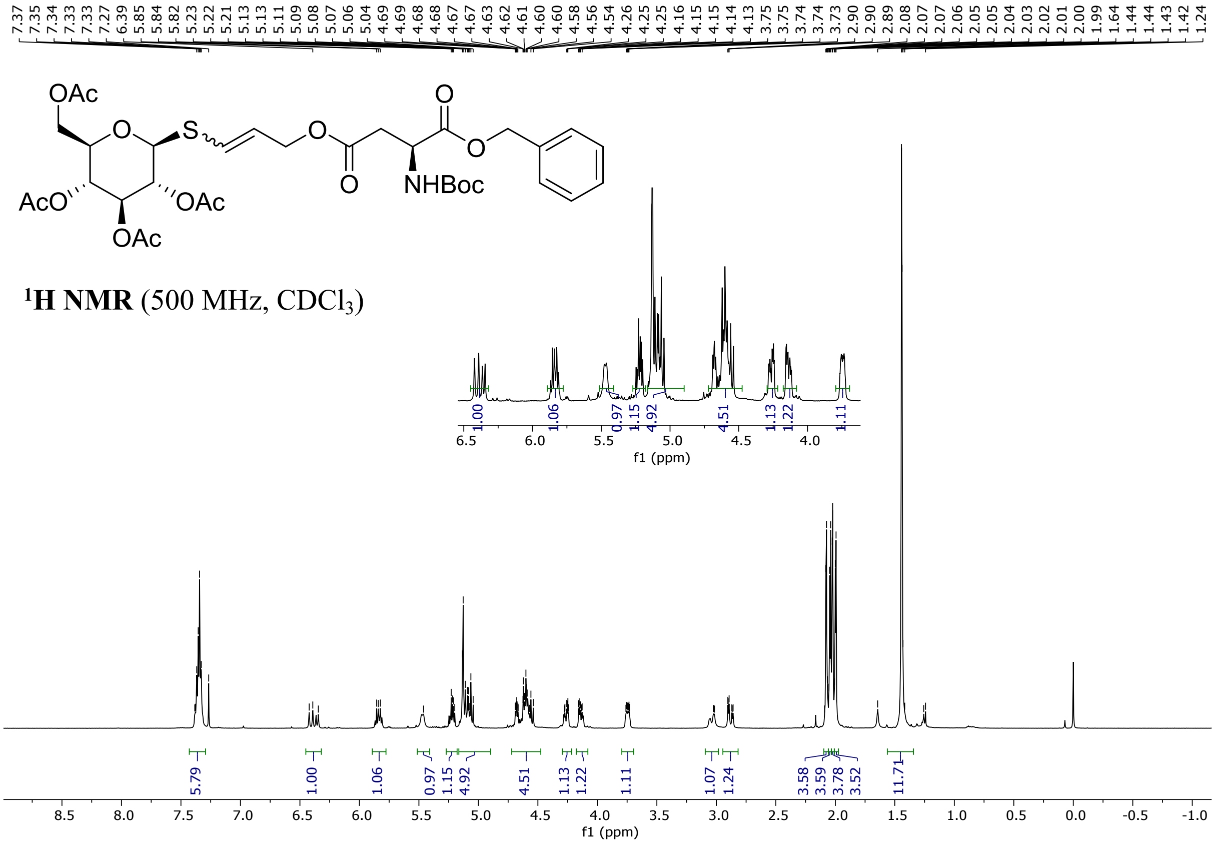

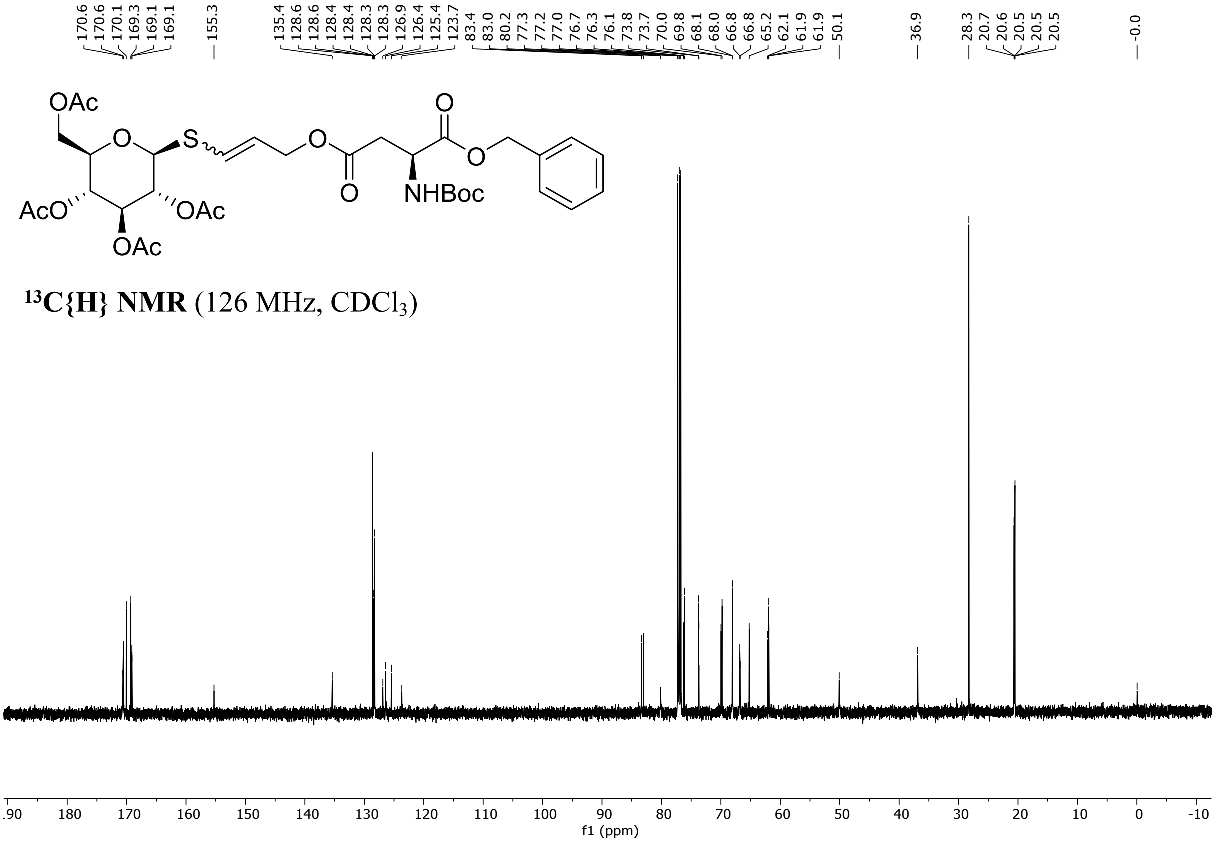

5.10. 1-(4-phenylbut-3-yn-1-yl)-4-(trifluoromethyl)benzene (20c)

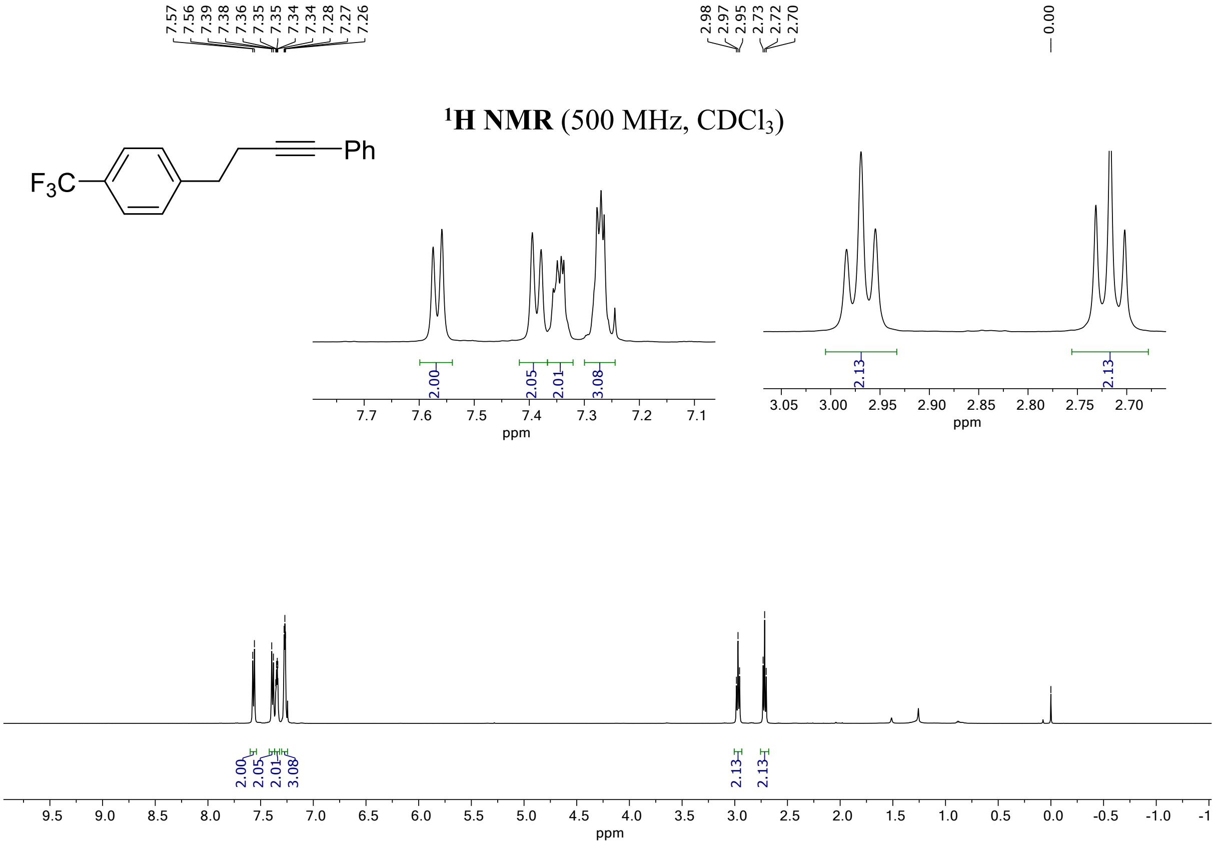

7.57
7.56
7.39
7.38
7.36
7.35
7.35
7.34
7.34
7.28
7.27
7.26
2.98
2.97
2.95
2.73
2.72
2.70
0.00
¹H NMR (500 MHz, CDCl₃)
F₃C
Ph
2.00
2.05
2.01
3.08
2.13
2.13
ppm

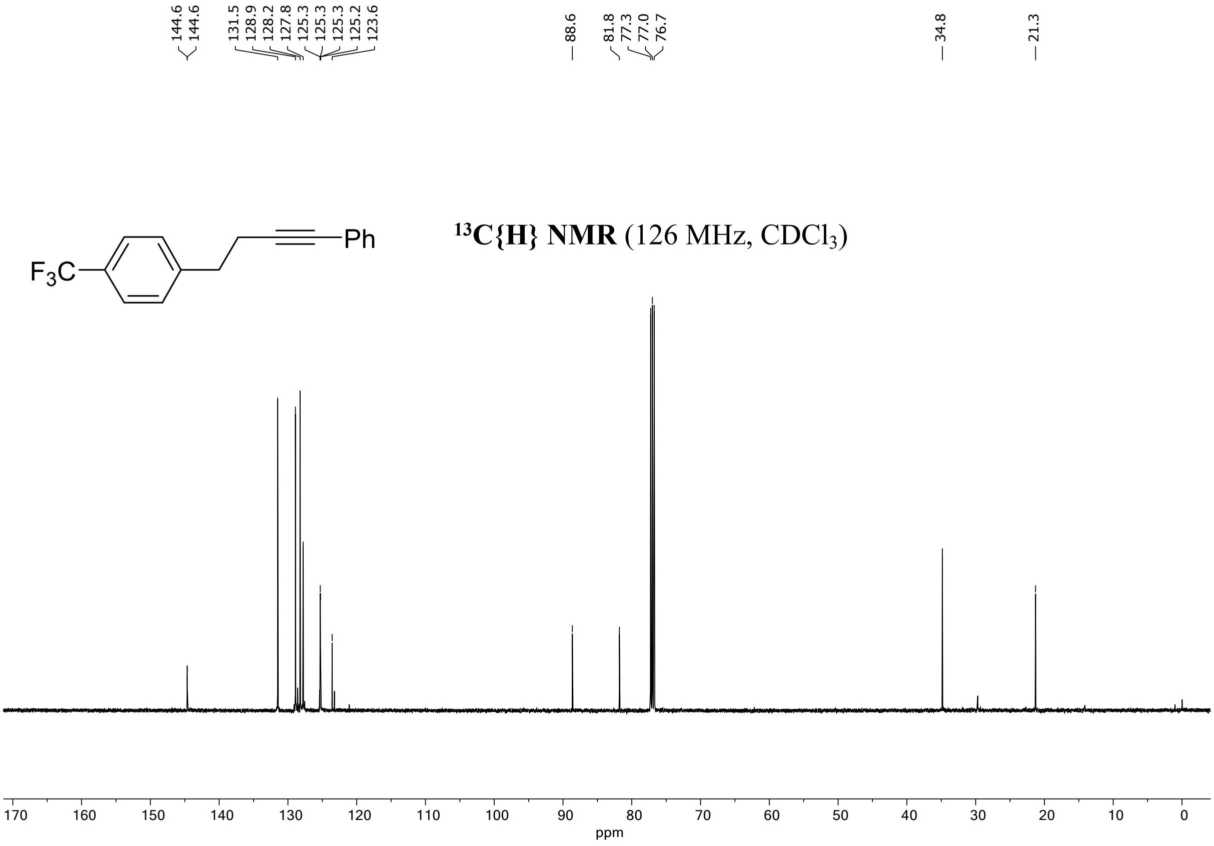

144.6
144.6
131.5
128.9
128.2
127.8
125.3
125.3
125.3
125.2
123.6
88.6
81.8
77.3
77.0
76.7
34.8
21.3
¹³C{H} NMR (126 MHz, CDCl₃)
F₃C
Ph
ppm

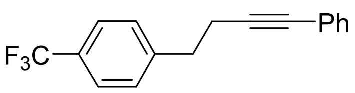

19F NMR (376 MHz, $CDCl_3$)

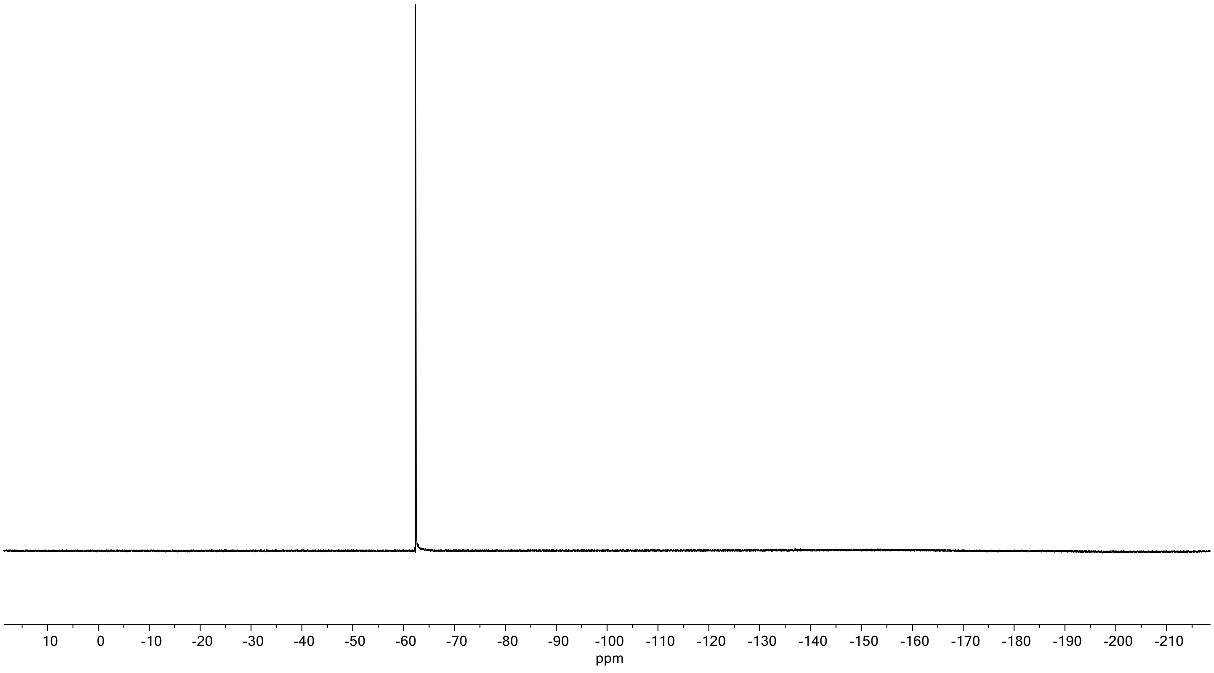

5.11. 1-(5-phenylpent-1-yn-1-yl)naphthalene (20f)

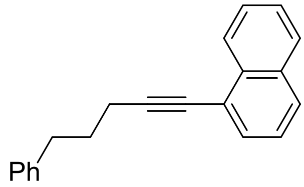

^{1}H NMR (500 MHz, $CDCl_3$)

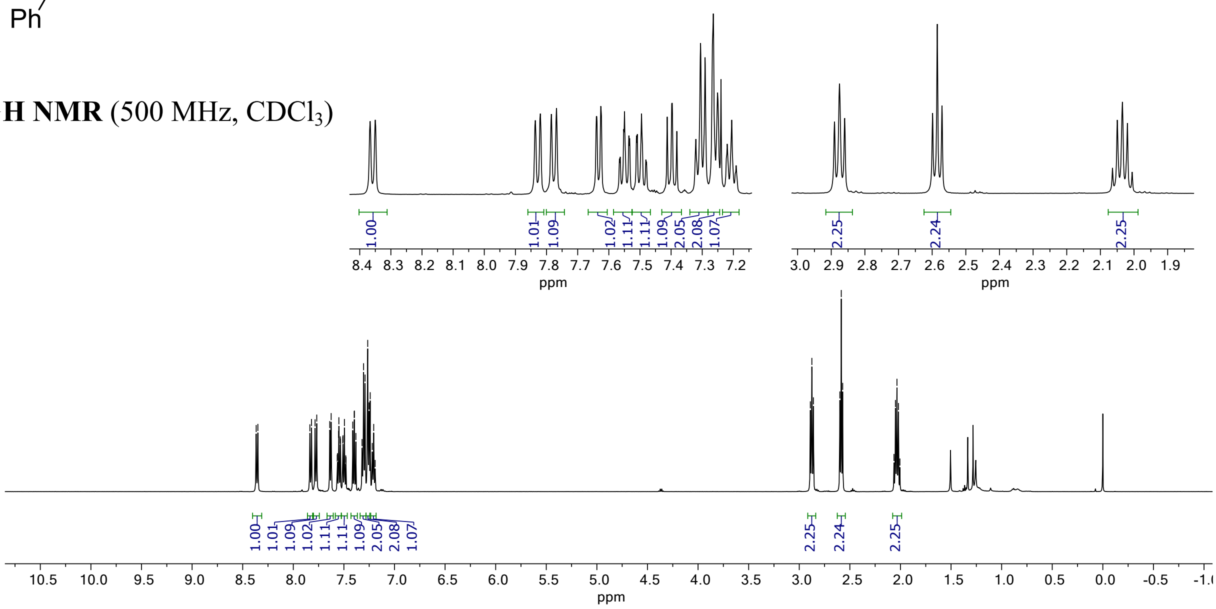

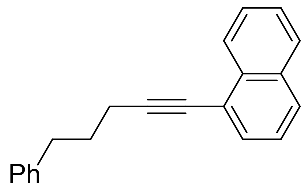

^{13}C{H} NMR (126 MHz, $CDCl_3$)

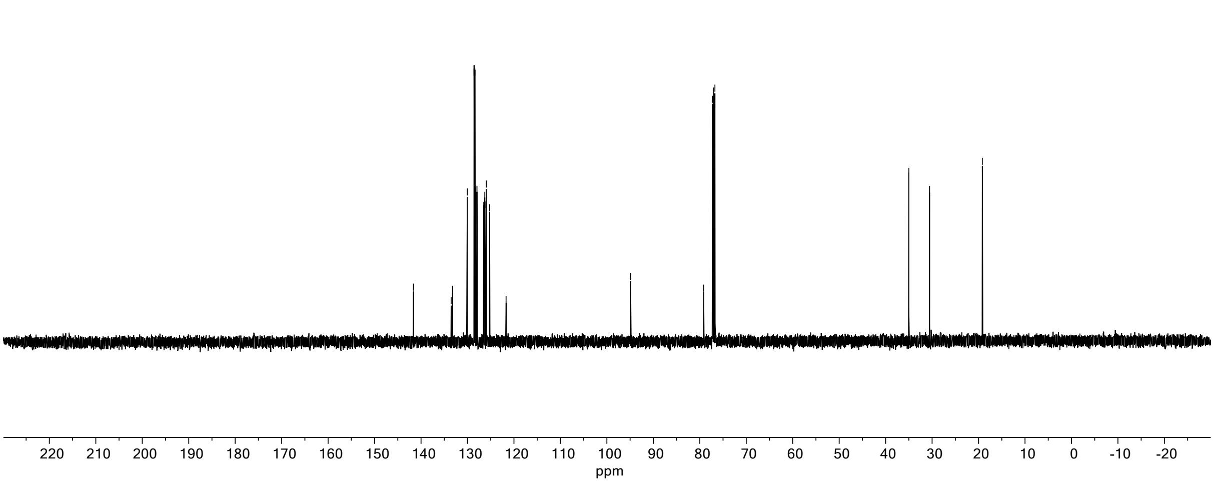

5.12. *tert*-butyldimethyl(3-(4-(4-(trifluoromethyl)phenyl)but-1-yn-1-yl)phenoxy)silane (20h)

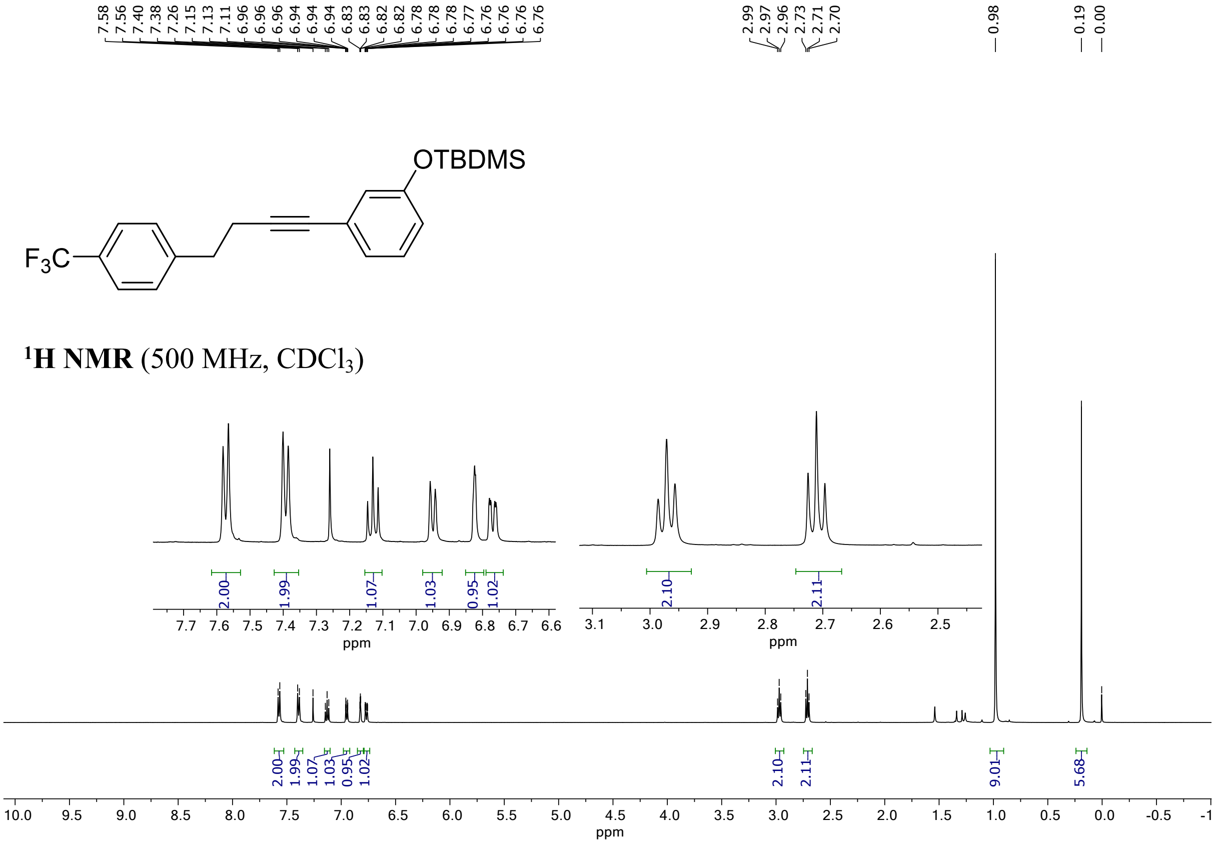

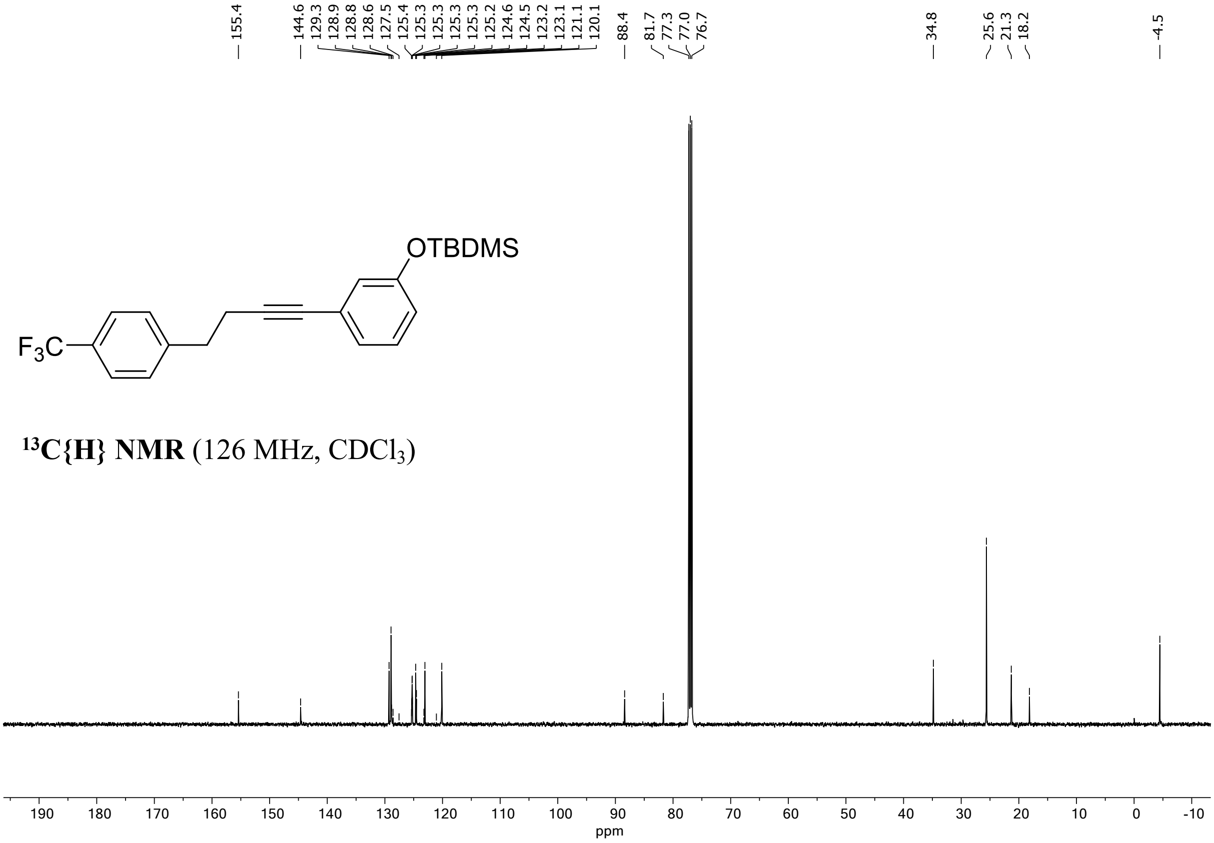

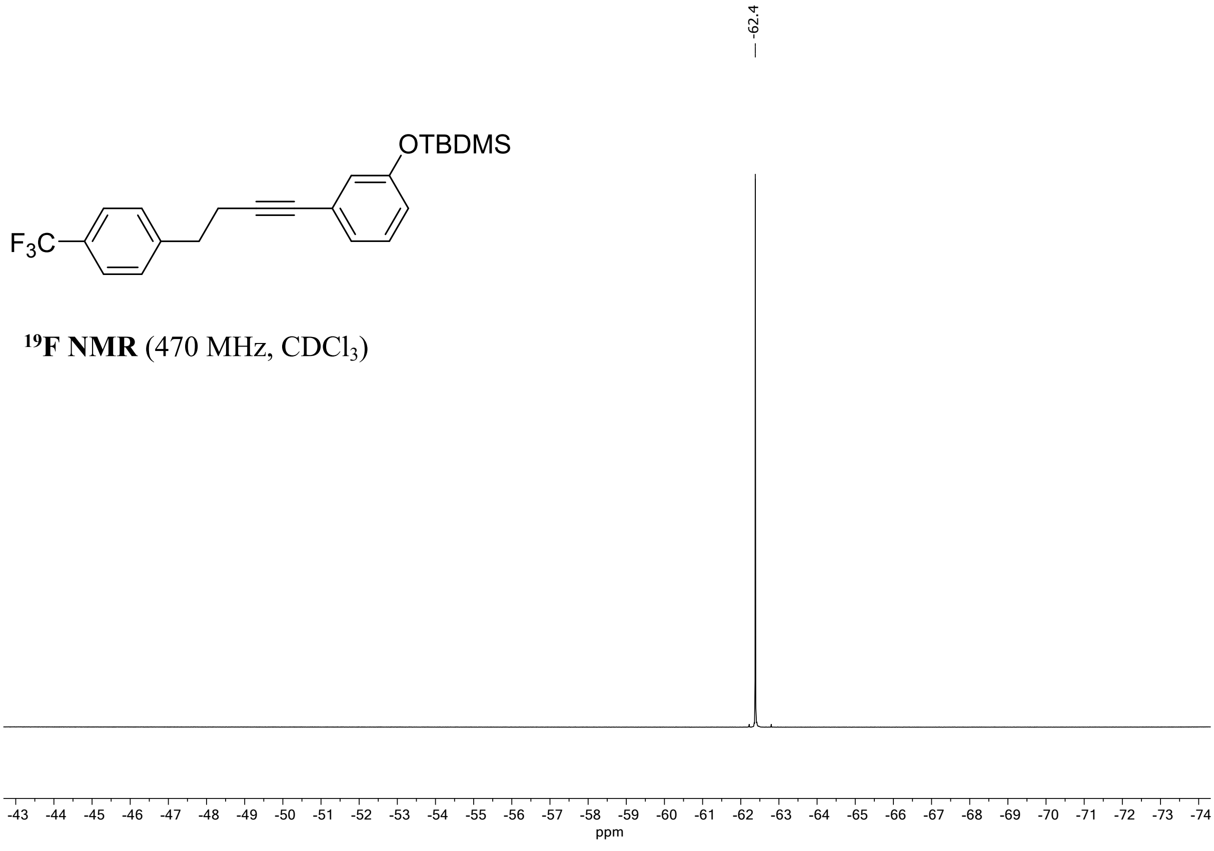

^{19}F NMR (470 MHz, $CDCl_3$)

5.13. 3-(5-phenylpent-1-yn-1-yl)phenyl (4*R*)-4-((5*S*,9*S*,10*S*,13*R*,14*R*,17*R*)-10,13-dimethyl-3,7,12-trioxohexadecahydro-1*H*-cyclopenta[a]phenanthren-17-yl)pentanoate (20l)

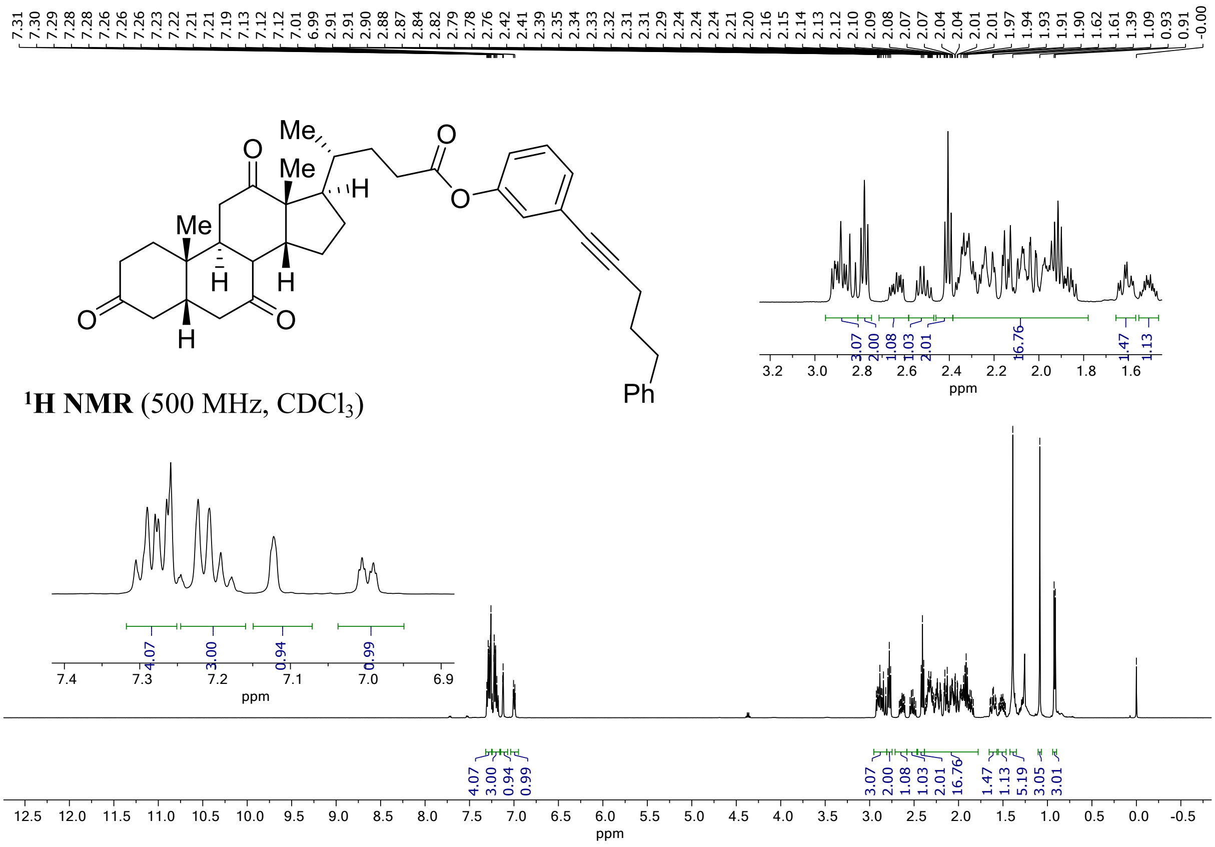

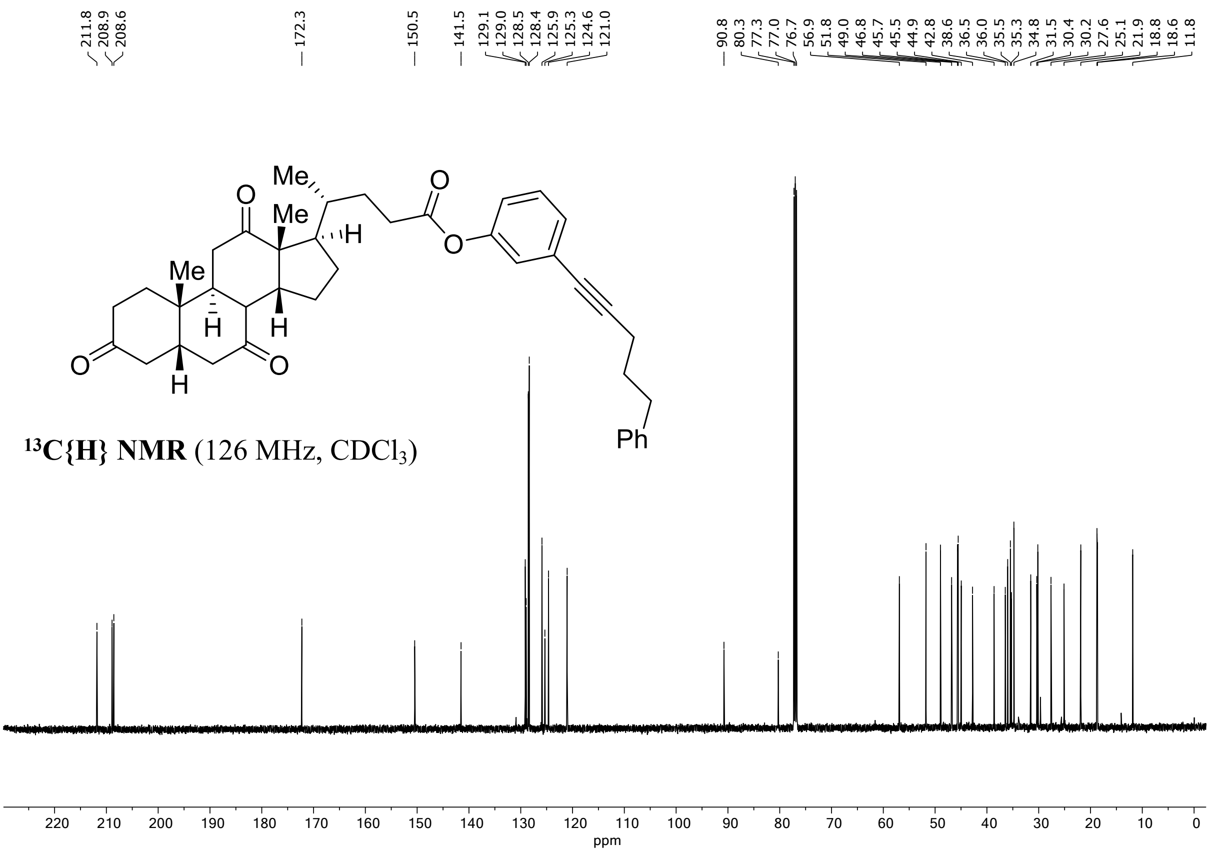

5.14. 3-(5-phenylpent-1-yn-1-yl)phenyl 2-(1-(4-chlorobenzoyl)-5-methoxy-2-methyl-1*H*-indol-3-yl)acetate (20n)

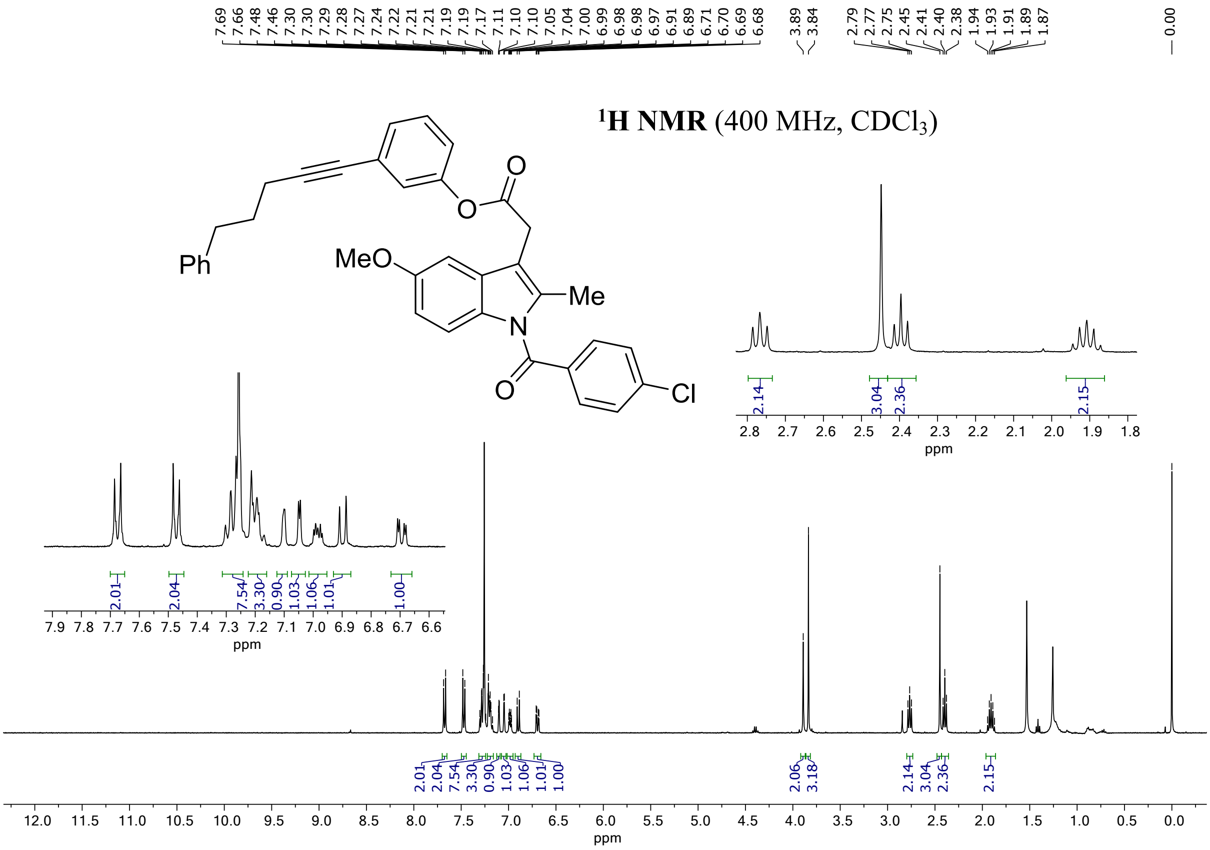

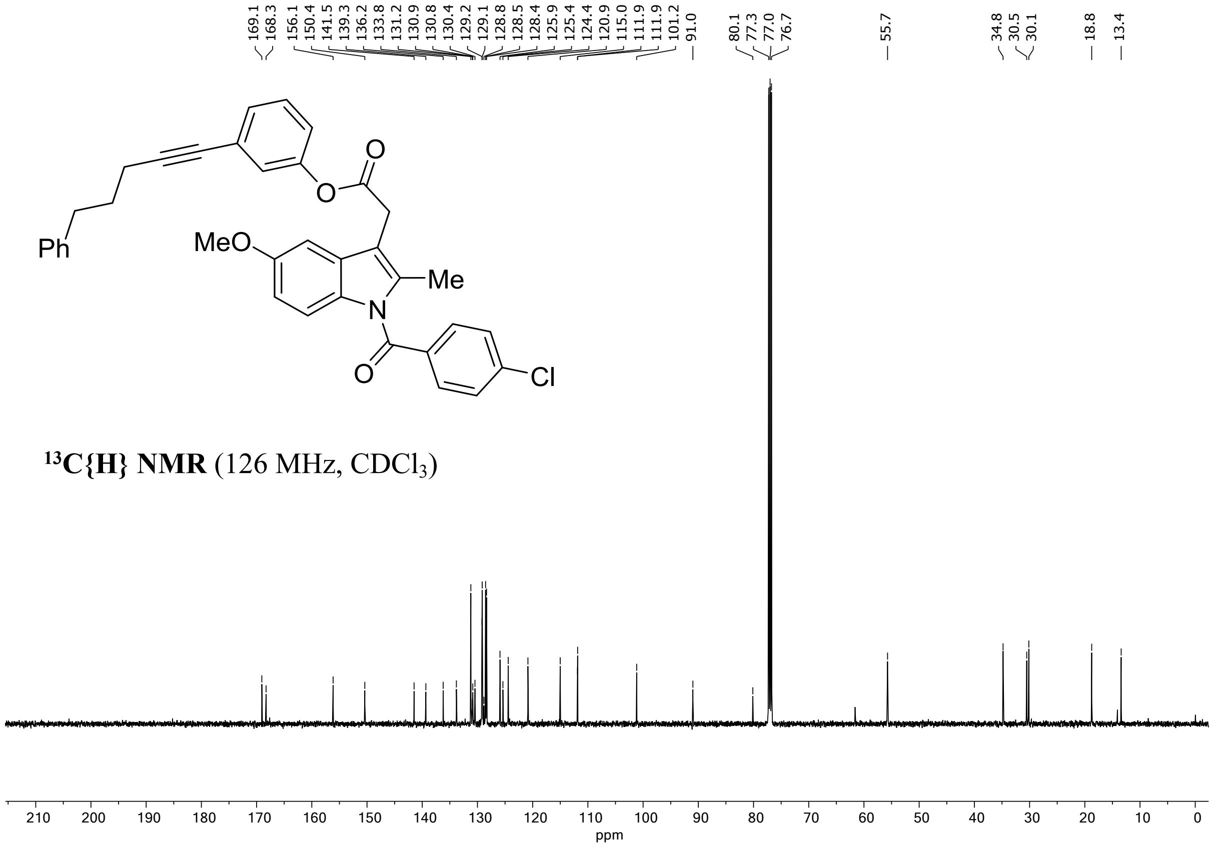

^{13}C{H} NMR (126 MHz, $CDCl_3$)
